# Promoter Analysis Reveals Globally Differential Regulation of Human Long Non-Coding RNA and Protein-Coding Genes

**DOI:** 10.1371/journal.pone.0109443

**Published:** 2014-10-02

**Authors:** Tanvir Alam, Yulia A. Medvedeva, Hui Jia, James B. Brown, Leonard Lipovich, Vladimir B. Bajic

**Affiliations:** 1 King Abdullah University of Science and Technology (KAUST), Computational Bioscience Research Center (CBRC), Computer, Electrical and Mathematical Sciences and Engineering Division (CEMSE), Thuwal, Saudi Arabia; 2 Center for Molecular Medicine and Genetics, Wayne State University, Detroit, Michigan, United States of America; 3 Department of Genome Dynamics, Life Sciences Division, Lawrence Berkeley National Laboratory, Berkeley, California, United States of America; 4 Department of Neurology, School of Medicine, Wayne State University, Detroit, Michigan, United States of America; Università degli Studi di Milano, Italy

## Abstract

Transcriptional regulation of protein-coding genes is increasingly well-understood on a global scale, yet no comparable information exists for long non-coding RNA (lncRNA) genes, which were recently recognized to be as numerous as protein-coding genes in mammalian genomes. We performed a genome-wide comparative analysis of the promoters of human lncRNA and protein-coding genes, finding global differences in specific genetic and epigenetic features relevant to transcriptional regulation. These two groups of genes are hence subject to separate transcriptional regulatory programs, including distinct transcription factor (TF) proteins that significantly favor lncRNA, rather than coding-gene, promoters. We report a specific signature of promoter-proximal transcriptional regulation of lncRNA genes, including several distinct transcription factor binding sites (TFBS). Experimental DNase I hypersensitive site profiles are consistent with active configurations of these lncRNA TFBS sets in diverse human cell types. TFBS ChIP-seq datasets confirm the binding events that we predicted using computational approaches for a subset of factors. For several TFs known to be directly regulated by lncRNAs, we find that their putative TFBSs are enriched at lncRNA promoters, suggesting that the TFs and the lncRNAs may participate in a bidirectional feedback loop regulatory network. Accordingly, cells may be able to modulate lncRNA expression levels independently of mRNA levels via distinct regulatory pathways. Our results also raise the possibility that, given the historical reliance on protein-coding gene catalogs to define the chromatin states of active promoters, a revision of these chromatin signature profiles to incorporate expressed lncRNA genes is warranted in the future.

## Introduction

Evidence for important, including essential, cellular and organismal roles of lncRNA in mammalian systems began to emerge prior to the advent of high-throughput genome and transcriptome sequencing. These early examples included the demonstration that the lncRNA XIST [1] was necessary and sufficient for X-chromosome silencing, as well as the discovery of SRA [2], an lncRNA that directly regulates the estrogen receptor α, one of the nuclear hormone receptors. Other essential functional ncRNAs in eukaryotic cells, such as ribosomal, transfer, and spliceosomal RNAs, have been well-known for an even longer time. Although the human genome project [3] initially focused almost exclusively on protein-coding genes in the human gene count, the ubiquity, in addition to the existence and the functional significance, of mammalian lncRNAs has been a key revelation of transcriptome sequencing projects [4].

Many lncRNA transcripts, similarly to mRNAs, are 5′-capped, polyadenylated, frequently spliced with conventional GT-AG intron excision, and readily evident in cytoplasmic polyA + RNA preparations; thousands of lncRNAs have been discovered from cDNA libraries [5], although abundant nuclear and polyA- lncRNAs have also been identified [6]. Up to one-third of polyA + lncRNAs encoded in the human genome may not be evolutionary conserved beyond primates [4]. In contrast, the majority of human protein-coding genes have pan-mammalian, and usually pan-vertebrate, conservation, many with homologs identifiable throughout metazoa. It has been suggested that non-conserved lncRNAs comprise a part of the molecular basis of species phenotypic uniqueness, distinguishing closely related species from one another by providing substrates for exaptation as well as adaptive evolution [7]. Despite their frequent lack of conservation, overwhelming evidence of lncRNA functions has emerged: they are characterized by diverse, positive and negative, nuclear and cytoplasmic, epigenetic and post-transcriptional regulatory modalities. Documented lncRNA functions include: positive regulation of sense mRNA translation by an antisense lncRNA [8], trans-repression of mRNAs by repeat-containing lncRNAs through the Stauffen-1 mRNA decay pathway [9]; epigenetic regulation of protein-coding targets by lncRNAs that recruit PRC2 to gene promoters [10], and direct RNA-protein interactions between lncRNAs and TFs: the Evf-2 lncRNA directly interacts with distal-less homeobox proteins to regulate mouse hippocampal development [11]. The Gas5 lncRNA contains a precise ribomimic of the genomic DNA binding site of the human glucocorticoid receptor, therefore titrating out bioavailable glucocorticoid receptor molecules and preventing them from binding their cognate sites in gene promoters along genomic DNA [12]. More generally, endogenous riboregulation of DNA-binding NHRs through direct interactions with lncRNAs [2], [12], [13] is an emerging leitmotif of post-genomic lncRNA biology.

These diverse functional mechanisms summarily indicate that jointly with TFs, lncRNAs are key regulators of protein-coding genes – including those that encode TFs. A prerequisite toward understanding the biology of lncRNAs is their assignment into tractable gene regulatory networks. We previously showed [14] that TFs – in particular, Oct4 and Nanog, which are essential for stem cell pluripotency [15] – bind directly at the promoters or within gene bodies of hundreds of lncRNA genes. ChIP-qPCR validation of TF binding to lncRNA gene promoters has elucidated numerous targets of key TFs, including non-conserved lncRNAs repressed by REST/NRSF in the human DiGeorge Syndrome critical region and in mouse [16]. We have used forward and reverse genetics to validate the regulation of lncRNAs by these TFs, uncovering feedback loops in the network that also use the lncRNAs to regulate these TFs during cell lineage specification [14]. More recently, we have assigned lncRNAs into deterministic regulatory networks, using reverse genetic approaches to show that a primate-specific antisense lncRNA regulates neuronal activity-dependent epileptogenesis in the *in vivo* human brain [17]. However, despite this progress, a genome-wide understanding of the lncRNA regulatory network – including the characterization of TF/lncRNA interactions – has to date remained elusive.

In this study, our goal was to computationally test the hypothesis that the global transcriptional regulatory programs of lncRNA genes and protein-coding genes are different. We set this problem within the framework of machine learning classification of promoters of these two broad gene classes. Previous studies [18]–[21] used support vector machines to distinguish non-coding RNAs (ncRNAs) from mRNAs, whereas experimental approaches including RiboSeq [22] and mass spectrometry [23] have documented that lncRNAs possess a low affinity for ribosomes and are rarely translated, but no comparable efforts have been devoted to comparing lncRNA and protein-coding gene promoters. Recently Lv et al. [24] used chromatin modification and genomic features to distinguish lncRNAs from protein-coding genes, while a statistical approach [25] singled out H3R2me1 as a distinctive histone mark between protein-coding genes and lncRNAs. Here, we interrogated multiple computational and empirical sources of regulatory information at promoters on a genome-wide scale. We found genetic and epigenetic signatures unique to protein-coding and lncRNA genes, respectively. These divergent promoter grammars may help to explain the observed differential and highly tissue- and condition-specific transcriptional regulation of lncRNA genes compared to their protein-coding counterparts in the same pathways. To our knowledge, this is the first demonstration that human lncRNA and protein-coding gene promoters contain sufficiently dissimilar information to be consistently distinguished with high accuracy. Our results summarily suggest the existence of distinct regulatory programs for these two gene groups.

## Results

### DNA sequence patterns at the promoters of protein-coding and lncRNA genes

**Figure 1:**
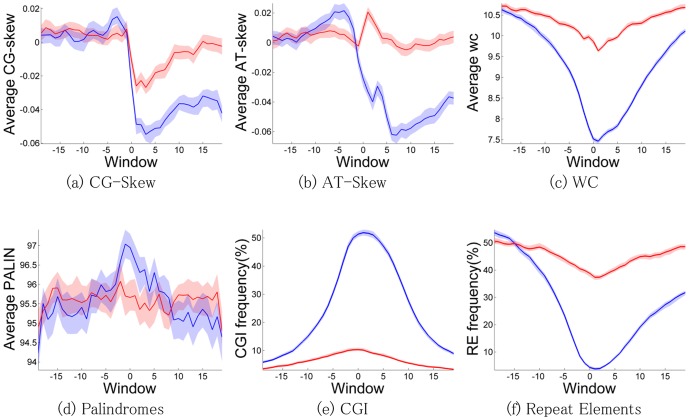
DNA feature distributions in the promoters of lncRNA genes and protein-coding genes. DNA feature distributions in a sliding window of 100 bp with a step of 50 bp in the promoters of protein-coding and lncRNAs. Blue line corresponds to promoters of protein-coding genes; red line corresponds to lncRNAs gene promoters. Figure 1a–d shows distribution of the feature in a sliding window of 100 bp with a step of 50 bp, resulting in 39 windows on the plot. Figure 1e–f show the percentage of promoters where features were found. Transparent regions correspond to 5–95% bootstrap confidence interval of the statistics. WC: word commonality, PALIN: palindromes, CGI: CpG Islands, RE: repetitive elements, all types of repeats except “simple repeats”, “low complexity regions” and “satellite repeats”. The enrichment score was calculated using right-sided exact Fisher's test (Table S3).

We compared DNA sequence promoter patterns of protein-coding and lncRNA genes. We found that A/T-rich mono-, di- and tri-nucleotide patterns are enriched at the promoters of lncRNA genes, relative to the promoters of protein-coding genes (“differentially enriched at lncRNA promoters”) (Table S1). CpG-derived mono-, di- and tri-nucleotide patterns are overrepresented in promoters of protein-coding genes. This result is broadly consistent with the observation that AT-rich promoters demonstrate lower expression but higher tissue specificity [26], properties known to define lncRNA promoters [4]. CG-skew, a feature of protein-coding gene promoters, is significantly reduced in lncRNA gene promoters, while AT-skew is almost depleted (Figure 1a–b). Figure 1c shows that word commonality score (Text S1 Methods section) is decreased around the transcriptional start sites (TSS) of lncRNA genes, although this depletion is stronger around TSSs of protein-coding genes, suggesting that lncRNA gene regulation, in contrast to protein-coding genes, is less driven by unique recognition sequences.

Palindromes, widespread regulatory elements in the promoters of protein coding genes [27], are less frequent around TSSs of lncRNA genes (Figure 1d). CpG islands (CGIs) are known to overlap with about two-thirds of protein-coding gene promoters [28]. Although CGIs are also hosting numerous non-coding transcripts [29], [30], an observation independent of the method of CGI detection [31], such ncRNAs are most likely short and unprocessed. On the contrary, we find that lncRNA promoters quite rarely overlap with CGIs (Figure 1e). LncRNA exons and splice junctions have been reported as enriched in repetitive elements [32]. We show that repetitive elements are also enriched at lncRNA promoters (Figure 1f, Figure S1). DNA sequence properties of non-zero similarly expressed protein-coding and lncRNA genes show feature patterns similar to those of the whole promoter sets without considering any expression levels (Figure S1).

### Known TFBSs and novel motif families distinguish the promoters of lncRNA genes

We *in silico* predicted the incidence of known transcription factor binding sequences (TFBSs) at the promoters of both gene types, using the HOCOMOCO [33] human TFBS models database. We found 74 TFBSs overrepresented in protein-coding gene promoters and 140 TFBSs overrepresented in lncRNA gene promoters (“differentially enriched in lncRNA promoters”) (Table S2).

Several TFs regulated by specific lncRNAs emerge as potential global regulators of lncRNA transcriptome in our analysis. A representative example is PGR (progesterone receptor), a nuclear hormone receptor (NHR), whose predicted TFBSs are differentially enriched at lncRNA promoters. The human PGR gene itself is *cis*-regulated by two lncRNAs: an lncRNA containing primate-specific repetitive elements provides transcriptional regulation [34], [35] and another *cis*-antisense transcript acts post-transcriptionally [36]. Here, we show widespread genome-wide association of lncRNA promoters with the same TF families that have been previously implicated as regulatory targets of lncRNAs. The human NHR superfamily provides the most abundant evidence of preferential involvement in genome-wide lncRNA cis-regulatory programs: the TFBSs of 13 (27%) of the 48 total known human NHRs (PGR, NR1I2, NR1I3, NR2C2, NR2E3, NR5A2, RARG, ESR2, PPARG, HNF4A, RXRB, ERR1, and ERR2) were differentially enriched at lncRNA promoters.

We additionally found that 14 FOX-family TFs, 6 SOX-family TFs, 3 members of the HOXD homeobox family, 3 members of the CEBP family, 3 NKX-family TFs, and 2 PPAR TFs (Table S2) demonstrate similar patterns of differential TFBS enrichment at lncRNA promoters. Several of these same TFs have been previously reported as regulatory targets of lncRNAs as well. NKX2-2 is endogenously regulated by a *cis*-antisense lncRNA at its own locus [37]. Similarly, the HOXD cluster is regulated in *cis* and in *trans* by multiple lncRNAs [38]–[40]. CEBPA is cis-regulated by an lncRNA as well [41]. Our *in silico* predicted binding site results for homeobox TFs at the promoters of lncRNA genes are consistent with a recent evolutionary study [42]. Summarily, the TF families that are characterized by TFBS enrichments at lncRNA promoters in our analysis include TFs that are known to be direct targets of lncRNAs from prior mechanistic studies.

The human proteome harbors approximately 1500 TFs [43], although TFBS models are available through HOCOMOCO for only 401 TFs. To compensate for this and to allow the detection of TFBSs whose motifs remain unknown, we applied *ab initio* motif discovery to genome-wide promoters, in order to complement the HOCOMOCO results. *Ab initio* identified motif families (MFs) generated by the Dragon Motif Finder [44], suggest multiple levels of sequence complexity specific to lncRNA promoters. These include reverse-complement motifs (palindromes) unique to lncRNA promoters, long motifs (20 bps), and polyA/polyT-rich regions (Figure S2a–d).

Condition-specific binding preferences are an important biological property of certain TFs [45]. Polymorphisms and *de novo* mutations may also alter a sequence of a particular binding site complicating known-TFBS discovery [46], [47]. Hence, we reasoned that certain *ab initio* MFs might reflect condition-driven, or protein complex-dependent, deviations from known TFBS models. We therefore compared the *ab initio* identified MFs to those already associated with known TFs. We confirmed five models and added one new TFBS model (NKX3-2) to our roster of lncRNA-promoter-enriched TFBSs (Table S2, Figure S2e–f).

### Chromatin configuration of lncRNA and protein-coding gene promoters

To test whether lncRNA and protein-coding gene promoters possess different epigenetic signatures, we compared the genomic overlap of the two promoter types with defined chromatin states (CSs) in eight human cell lines [48].

Protein-coding gene promoters more often overlapped CSs associated with active, weak or inactive/poised promoters, and were also more strongly enriched for Polycomb-repressed regions. Relative to protein-coding gene promoters, those of lncRNA genes more often overlapped CSs associated with insulators, regions of transcriptional transition (regions located between the initiation and elongation histone marks), elongation, weak transcription and heterochromatin (Figure S3, Table S3). After the subsets of lncRNA and protein-coding genes with similar expression levels in different cell lines were selected (see Methods), the same tendency remains but the difference between the promoters of protein-coding and lncRNA genes becomes less pronounced (Figure S5, Table S6). The role for enhancer-associated lncRNAs in regulating protein-coding genes over large genomic distances was recently reported [49]. Our data shows that in genes with non-zero similar expression levels most of the enhancer states are overrepresented in lncRNAs vs protein-coding genes (Figure S5), while in six out of eight studied cell types for all (independent of the expression levels) promoters only one out of four enhancer-associated CSs (weak enhancers) displays significant overrepresentation at lncRNA versus protein-coding gene promoters (Text S1 Results section).

**Figure 2:**
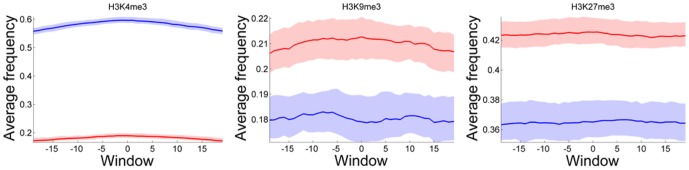
Distribution of histone modification marks in the GM12878 cell line across lncRNA and protein-coding gene promoters. Figure demonstrates fraction of all promoters covered by chromatin a particular mark. Blue line corresponds to promoters of protein-coding genes; red line corresponds to lncRNA gene promoters. Transparent regions correspond to 5–95% bootstrap confidence interval of the statistics.

To understand the biological context of the heterochromatin CS enrichment at lncRNA gene promoters, we analyzed histone modification marks (HMs) in the ENCODE Tier 1 cell line GM12878. LncRNA gene promoters were significantly depleted of almost all histone modification marks, except for H3K27me3 and H3K9me3 (Figure 2, Figure S4). H3K27me3 contributes to maintenance of ‘bivalent domains’, transcriptionally-poised regions combining activating and repressing histone marks [50], [51], suggesting that lncRNA promoters are not permanently repressed and could be subject to activation under specific conditions. H3K9me3 marks transcriptional repression [52] but is also found in certain transcribed regions [53], and may be involved in elongation [54]. After the subsets of lncRNA and protein-coding genes with non-zero and similar expression levels in different cell lines were selected, lncRNA gene promoters demonstrated enrichment for H3K9me3 and surprisingly for H3K36me3 in all tested cell types. H3K36me3 is a mark of transcriptional elongation [55], [56]. Interestingly, lncRNA gene promoters demonstrate a decreased level of H3K27me3 and, in H1-hESC, an increased level of H3K27ac, a mark of active promoters and enhancers [57]. Taken together, these results support active chromatin organization of lncRNA promoters, yet distinct from the one of protein-coding genes.

### Distinguishing promoters of protein-coding and lncRNA genes through an ensemble of decision trees model

**Figure 3:**
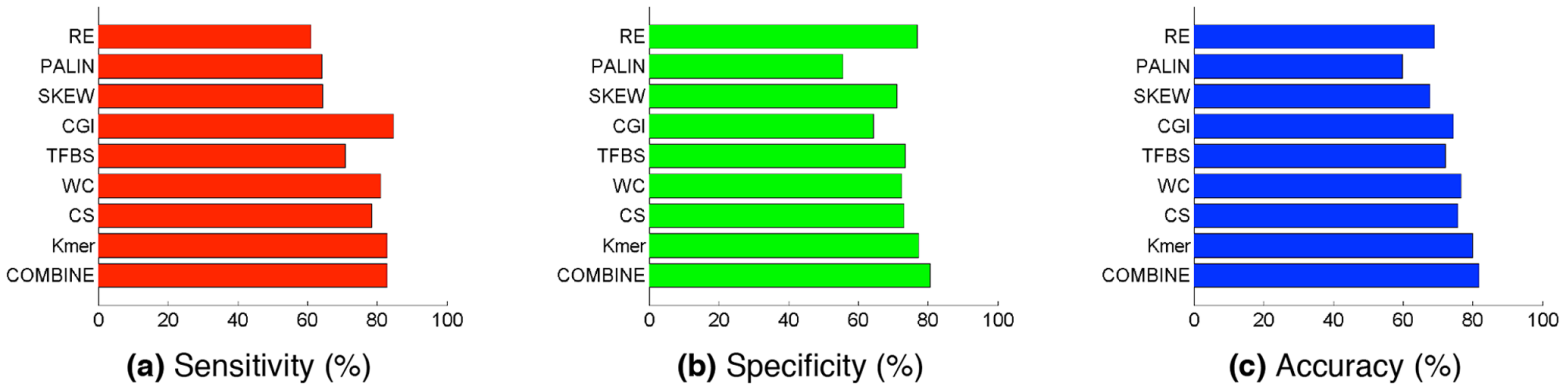
Performance of the prediction model. Quality of the models based on the complete feature set and several combinations of features. RE: repetitive elements, PALIN: palindromes, SKEW: A/T and C/G skews, CGI: CpG Islands, TFBS: transcription factor binding sites, WC: word commonality, CS: chromatin states, k-mer: mono-, di-,tri-nucleotide frequencies, COMBINE: combination of all types of features for complete promoter set (CPS).

Several lines of evidence indicate that the transcriptional regulation of lncRNAs may differ substantially from that of protein-coding genes. To computationally test for any evidence of this phenomenon, we leveraged recent advances in machine learning to fit an integrative model based on the information from all analyzed data types to distinguish the promoters of protein-coding genes from those of lncRNAs. Our fitted ensemble model correctly classified the promoters (lncRNA or protein-coding) with more than 80% accuracy. Hence, across the majority of the genome sequence space, genetic and epigenetic information is sufficient to confidently separate these two classes of promoters (Table 1, Table S4). Interrogation of our fitted models revealed that the strongest effects accounting for this predictive power are DNA k-mers and CSs. These were more discriminative than TFBSs, although most feature types, including TFBSs, had significant discrimination power (Figure 3).

**Table 1:** Summary of the results for separation of promoters of protein-coding and lncRNA gene promoters using different combinations of features.

		Considering [−1000, +1000] bp			Considering [−1000,0] bp		
Features	#Feature	Sensitivity (%)	Specificity (%)	Accuracy (%)	Sensitivity (%)	Specificity (%)	Accuracy (%)
K-mer	168	82.750	77.208	79.957	76.032	70.788	73.389
CS	105	78.428	73.013	75.699	77.216	72.992	75.087
WC	39	80.862	72.476	76.635	76.995	67.573	72.246
TFBS	426	70.790	73.476	72.144	67.853	71.97	69.928
CGI	39	84.513	64.278	74.315	82.063	62.852	72.38
SKEW	78	64.261	70.948	67.632	54.849	59.791	57.34
PALIN	39	64.132	55.554	59.808	52.886	54.841	53.871
RE	39	60.891	76.867	68.943	43.685	69.601	56.747
COMBINE	933	82.788	80.609	81.690	79.672	73.732	76.678
CS + CGI	144	82.582	72.508	77.504	77.774	72.886	75.31
All except K-mer, WC	726	81.219	79.438	80.321	78.699	73.923	76.292
All except	687	81.371	79.555	80.456	78.628	73.977	76.284
K-mer, WC, PALIN							

K-mer: mono-, di-,tri-nucleotide frequencies, CS: chromatin states, WC: word commonality, TFBS: transcription factor binding sites, CGI: CpG Islands, SKEW: A/T and C/G skews, PALIN: palindromes, RE: repetitive elements, COMBINE: combination of all types of features.

Since we had originally considered the regions of [−1000, +1000] bp around the TSS (Dataset S2) as a putative promoter region, for protein-coding genes we might have included some coding exonic sequences, therefore introducing coding sequence bias. To avoid this, we also performed the analysis (Text S1 Methods section) using only upstream promoter regions ([−1000, 0] bp upstream of the TSS). Using this promoter set, we were able to distinguish between lncRNA and protein-coding gene promoters with 77% accuracy (Table 1, Table S5). Moreover, to avoid a bias caused by the more abundant presence of CGIs at protein-coding gene promoters, we built another model for the upstream promoter regions ([−1000, 0] bp, Dataset S2) having no overlap with CGIs (Text S1 Methods section). Although the performance of the model decreased, we were still able to distinguish between lncRNA and protein coding gene promoters with 71% accuracy (Table S5).

### Distinguishing promoters of protein-coding and lncRNA genes with similar expression levels

LncRNAs show lower expression in almost all cell types as compared to mRNAs [49]. Low and highly expressed promoters tend to have distinct epigenetic features [58], [59]. Sequence specific differences of high and low expressed genes have been discussed for years [60]. To avoid a potential bias of differentiating low and highly expressed promoters rather than lncRNA and protein-coding promoters, we compared only the lncRNAs and protein-coding genes with similar expression level in several cell types. Our model achieves over 81%, 80%, 81% and 80% accuracy for Gm12878, H1-hESC, K562 and HUVEC, respectively (Table 2) when expression levels were controlled for. If we exclude the CGIs and downstream regions from the consideration, the models still demonstrate over 76% accuracy. Importantly, the performance of the model has been increased as compared to the model with the same set of features, but without controlling for the expression levels (71%, see previous section, vs 76%). These results suggest that expression bias is very unlikely to contribute to the accuracy of the models.

**Table 2:** Summary of the results for separation of promoters from protein-coding and lncRNA genes having similar expression pattern in different cell lines.

	Features	Considering [−1000, +1000] bp			Considering [−1000,0] bp		
Cell line		Sensitivity (%)	Specificity (%)	Accuracy (%)	Sensitivity (%)	Specificity (%)	Accuracy (%)
GM12878	COMBINE	81.709	80.532	81.120	79.397	73.895	76.646
H1-hESC	COMBINE	80.851	80.562	80.706	79.726	73.237	76.482
K562	COMBINE	81.484	81.326	81.405	79.144	74.796	76.970
HUVEC	COMBINE	80.260	80.728	80.494	79.763	73.124	76.443
GM12878	All except CGI	80.74	81.225	80.983	78.745	74.086	76.416
H1-hESC	All except CGI	80.035	80.737	80.386	78.713	74.385	76.549
K562	All except CGI	80.975	81.472	81.224	78.555	74.743	76.649
HUVEC	All except CGI	79.628	80.134	79.881	78.427	74.494	76.461

CGI: CpG Islands, COMBINE: combination of all types of features.

### Open chromatin and specific regulatory programs at lncRNA-enriched TFBSs

We aimed to assess the extent of experimental support for transcriptionally permissive chromatin configurations across all TFBSs enriched at lncRNA promoters. We reasoned that binding sites should have an open chromatin configuration in the cell or tissue types where binding occurs. We leveraged the empirical genome-wide catalog of DNase I hypersensitive sites, indicative of open chromatin, derived for 125 human cell types by the ENCODE Consortium [61]. We found that 67 of the 140 lncRNA-TSS-enriched TFBSs were significantly associated with hypersensitive sites in one or more cell types. This presence of DNase I hypersensitivity sites in lncRNA promoters supports the regulatory potential of such regions in at least one of the 125 studied cell types, despite the repressed chromatin conditions at their promoters in the eight cell types with available data in the CS analysis above.

In parallel, we overlapped lncRNA-promoter-enriched TFBSs with ENCODE ChIP-seq experimental evidence for the corresponding TFs across all ENCODE ChIP-seq datasets [62]. A moderate FDR approach (Benjamini-Hochberg procedure) identified three TFs – GATA3, ARID3A, and MEF2A – as being dually supported by HOCOMOCO computational evidence of their TFBS overrepresentation at lncRNA promoters and by ENCODE ChIP-seq experimental evidence for their binding at lncRNA promoters genome-wide (Table S2). This intersection of TFBS overrepresentation at lncRNA promoters and empirical ChIP-seq support for the binding of these same TFs at those promoters provides important evidence that these three TFs may direct genome-wide lncRNA transcriptional programs in the ENCODE ChIP-seq-profiled cell types.

## Discussion

We present the first genome-wide demonstration of a significant difference in sequence characteristics between the promoters of human lncRNA and protein-coding genes, suggesting distinct regulation of the two gene groups. In view of the frequent bidirectionality of human promoters that simultaneously give rise to protein-coding and lncRNA genes [63], the distinctions we find are all the more remarkable, since bidirectional promoters are counted by our approach as both protein-coding and lncRNA. We speculate that specific TFs may function as network nodes that not only accept directional edges from regulatory lncRNAs, but also serve as network hubs that extend multiple new directional edges toward other lncRNA genes whose promoters contain their cognate TFBSs. In particular, our study, for the first time, suggests that specific NHRs - members of the nuclear receptor family, which are already known to be targeted by lncRNA-protein interactions that join lncRNAs and NHRs in ribonucleoprotein complexes – in turn, may regulate lncRNA transcription through promoter binding. Among the other TFs we considered, GATA3, ARID3A, and MEF2A have the singular distinction of being significantly supported by all three lines of evidence: TFBS motif enrichment at our 18,000 lncRNA promoters, DNase I hypersensitive site overrepresentation at their TFBS-containing lncRNA promoters, and ChIP-seq experimental evidence of enriched binding at these promoters genome-wide, across the ENCODE DNase I- and ChIP-seq-profiled cell and tissue types. GATA3, one of our most-enriched TFs at lncRNA promoters and an essential regulator of type 2 helper T-cell (Th2) cytokine production, is itself cis-regulated by an antisense lncRNA (GATA3-AS1), which is increased in patients with allergic rhinitis, a Th2-associated disease [64]. More recently, evidence for large-scale GATA3 regulation of lncRNAs associated with Th2 functions has emerged, and an lncRNA was assigned into a GATA3-containing regulatory network in Th2 cells [65]. Our results support large-scale regulation of lncRNA transcription programs by GATA3, and enhance the list of lncRNAs whose promoters may comprise GATA3 targets.

Our observation that lncRNAs might be selectively regulated by a distinct set of TFs has substantial implications for systems biology: cells are potentially capable of harnessing a defined subset of regulatory switches to toggle the expression levels of lncRNAs without altering mRNA levels. Most of the disease-associated sequence variants in the human genome are non-coding [66], necessitating an integration of lncRNA TSS and exon locations with the increasingly abundant common-variant Genome Wide Association Studies (GWAS), as well as throughout whole-exome and whole-genome resequencing datasets designed to capture rare, large-effect disease-associated variants. Our results empower the GWAS community to re-annotate cryptic disease-associated variants at *in silico* predicted TFBSs that we have linked to global catalogs of lncRNA promoters and to lncRNA regulatory programs modulated by specific TFs. By virtue of their TFBS localization, such variants may emerge as direct functional candidates.

Our lncRNA gene collection is a composite of three previously published lncRNA sources – Gencode [4], the Broad Institute [67], and our own [5] – and three additional public lncRNA collections (see Methods). The methods used in the compilation of these lncRNA collections rely on a combination of full-length cDNAs, deep-coverage RNAseq, targeted RTPCR validation, and extensive manual curation. Therefore, the lncRNA genes that we used are largely as reliable in terms of their underlying evidence and annotation quality as protein-coding genes, and the differences we have uncovered relative to their protein-coding counterparts are not likely to be due to annotation disparities.

Until recently, only protein-coding gene sets were used in characterizing general promoter features. Therefore, some widely accepted promoter features and chromatin state signatures may be biased as a consequence of having been inferred from protein-coding genes. In this context, it is hardly surprising that certain sequence and epigenetic features, more specific for protein-coding genes, are less pronounced at lncRNA promoters, while the chromatin states associated with lncRNA promoters are predominantly labeled as inactive promoters. However, these labels were based on manual annotation by biologists, predominantly of protein-coding and intergenic regions [68]. Hence, while it is true that genomic regions with these state-labels tend to be transcriptionally less active than protein coding regions on average in cell lines and tissues explored to date, this does not exclude the possibility that there may exist novel chromatin states associated with lncRNA promoters that have yet to be identified by genome-wide Hidden Markov Model (HMM) based chromatin studies. One of the reasons for this may be a low level of all histone modification signals in lncRNA gene promoters corresponding to low expression of lncRNAs, making these promoters appear more heterochromatin-like than protein-coding gene promoters. Another possibility is that due to high tissue-specificity of lncRNA expression, most lncRNA genes are repressed in each cell type from the limited repertoire of cells that we analyzed. LncRNAs can impact regulatory outcomes despite their low expression levels; for instance, the XIST lncRNA, expressed as a single genomically-tethered copy, recruits repressive histone modifiers to the allele from which it was expressed, leading to the inactivation of nearly an entire X-chromosome [69]. Therefore, the promoter characteristics of low-abundance, but functional, lncRNAs merit inclusion in future global definitions of human promoterome properties. A growing number of lncRNAs has recently been shown to exert diverse regulatory functions. Our characterization of the global selective regulation of lncRNA genes places three known human transcription factors at the nexus of empirical and computational evidence for their role in such regulation, enhancing our understanding of how the relationship of TFs and their lncRNA gene targets impacts the transcriptional and post-transcriptional regulatory networks that govern human gene expression.

## Methods

### A non-redundant set of promoters for human protein-coding and lncRNA genes

We used RefSeq transcripts from the UCSC Genome Database (http://hgdownload.cse.ucsc.edu/goldenPath/hg19/database/refGene.txt.gz, download date: 14 January, 2013) for the human genome (version hg19). Out of the 44,140 transcripts, we considered only the 34,475 that were clearly protein-coding (i.e. having an NM RefSeqID) and that were located on chromosomes 1–22, X, and Y. To construct a non-redundant (a single reference transcript per gene) set, we considered at least 1 bp overlap in the entire genomic span (including exons and introns along hg19 coordinates) among all transcripts located on the same strand in the same locus, and we randomly selected one transcript per locus. Through these filtering steps, we ultimately arrived at 18,789 protein-coding non-redundant representative transcripts conforming to our one-transcript-per-gene data structure (Dataset S1a).

We also assembled 18,498 (Dataset S1b) experimentally supported (with full-length cDNA, Broad Institute RNAseq, or Gencode-curated cDNA and/or expressed sequence tag, i.e. EST, evidence), non-redundant (with respect to genomic position and orientation) lncRNA genes from six published sources: 1) our manually annotated list of human lncRNAs that are supported by full-length cDNA clones from 5′cap-trapped, dT-primed libraries [5]; 2) the Broad Institute lincRNA resource consisting of transcript assemblies inferred from exhaustive RNAseq of a human tissue collection [67]; 3) the ENCODE Consortium's official Gencode catalog [70] of human genes (www.gencodegenes.org), a manually curated list of coding and non-coding genes that are supported by full-length cDNA, EST, RNAseq, and targeted-RTPCR evidence from public sources as well as from Gencode's ongoing validation efforts. We enhanced this collection with non-redundant lncRNAs from three additional sources: 4) NCBI RefSeq (NR identifier) non-coding transcripts that do not host any known small RNAs according to the UCSC Genome Database sno/miRNA repository, 5) human ESTs from the dbEST division of Genbank (NCBI) [71] that were submitted by RIKEN (Japan) and that mapped beyond 10 kb from any protein-coding gene, and 6) manually annotated lncRNAs from human sense-antisense pairs [72], [73]. The majority of validated, literature-supported, non-hypothetical (Genbank identifier series: NM) RefSeq protein-coding genes are supported by full-length cDNAs. Gencode applies a unified set of manual annotation and targeted validation standards to uniformly assign biotypes to all transcripts and genes throughout its coding and non-coding gene collections, precluding lncRNA-specific quality control bias.

For each gene's representative transcript, we considered the [−1000, +1000] bp around the TSS as the putative promoter region, except in the specific analyses listed under Results where an alternate [−1000, 0] bp TSS set was used. We chose relatively large promoter regions with the purpose of incorporating alternative TSS, which in turn allowed us focus on gene-based rather than transcript-based analysis, since alternative promoter usage is a widespread phenomenon in human transcriptome [74], [75]. Although such promoters may incorporate some exonic sequence, it was shown that downstream elements also regulate transcription [76], and therefore including the first kilobase of gene bodies – provided that protein-coding gene properties such as codon bias are controlled for – can provide valuable regulatory information in addition to that residing in the region upstream of the TSS. We obtained the promoter sequences using Galaxy (www.galaxyproject.org/).

### Computational model to discriminate the promoters of protein-coding and lncRNA genes

To identify the regulatory patterns which may facilitate the computational discrimination between the promoters of protein-coding genes and lncRNA genes, we extracted features from several broad categories. These include various frequency-based properties of the promoters such as k-mers, word commonality, skew, palindromes; regulatory elements such as CpG islands, repetitive elements, TFBS found within the promoter regions; epigenetic features such as chromatin states and separate histone modification marks (see Text S1 Methods section). We used an ensemble of decision trees [77] to generate a classification model and estimate its accuracy with 20-fold cross-validation.

### Transcription factor binding sites (TFBSs) enrichment

We predicted TFBSs using 426 position weight matrices (PWMs) for 401 human TFs from the HOCOMOCO [33] database (v.8) (http://www.cbrc.kaust.edu.sa/hocomoco/Download.php) in the promoters of both protein-coding and lncRNA genes. Since the extent to which the original nucleotide composition of promoters is a cause or a consequence of the possible TFBS repertoires present in these promoters is unclear, we used the same strategy for both protein-coding and lncRNA promoters. For each PWM the threshold was set in the following way: for a random word generated by a background model (independent nucleotide distribution with nucleotide frequency of hg19) there was a fixed probability of 0.0005 to obtain the PWM score no less than the threshold. We generated 426 features using the binary value 0 or 1 (zero or non-zero hits above the threshold in a given promoter sequence in both strands). We selected significantly overrepresented TFBSs in promoters of protein-coding vs. promoters of lncRNA (and vice versa) gene sets (p-value < = 0.05, right sided Fisher's exact test with Benjamini-Hochberg multiple testing for controlling false discovery rate (FDR) [78]) (See Text S1 Methods section).

### Expression analysis using RNA-seq data

We used RNA-seq data from Gm12878, H1-hESC, K562 and HUVEC cell lines to check the model performance, when expression levels of lncRNAs and protein-coding genes are similar. We used the mappings, provided by ENCODE (http://hgdownload.cse.ucsc.edu/goldenPath/hg19/encodeDCC/wgEncodeCshlLongRnaSeq/) and we quantified the expression levels as RPKM (read per kilobase of exon per million mapped reads) [79] using FluxCapacitor [80]. We excluded all the transcripts having RPKM  = 0. To identify the lncRNA and protein-coding genes with similar expression distribution, for each lncRNA we selected a protein-coding gene with the nearest expression value (but not differing more than 1% of its expression level) (Text S1 Methods section). In this way we secured a one-to-one correspondence between lncRNA genes and protein-coding genes matching based on their expression level, thus avoiding any kind of possible expression bias between lncRNA and protein-coding genes (Figure S6, Dataset S3).

### Compilation of a uniform list of synonymous human transcription factor names

We used the UniProt database (www.uniprot.org) and the GeneCards resource (www.genecards.org) to compile a comprehensive list of human transcription factors that accounts for all name multiplicity, synonymity, and redundancy between the abbreviated transcription factor names used by HOCOMOCO (since Uniprot naming is in one-to-one relationship with the HOCOMOCO naming system) and ENCODE. We manually curated this list. We identified 106 (DataSet S1i) transcription factors common to both lists, and all searches for overlaps between HOCOMOCO computational TFBSs and ChIP-seq empirical TFBSs were performed using this list.

See Text S1 Methods section for additional information.

## Supporting Information

Figure S1 **DNA feature distributions in a sliding window of 100 bp with a step of 50 bp in the promoters of protein-coding and lncRNAs for complete promoter set (CPS).** Green line corresponds to promoters of protein-coding genes; black line corresponds to lncRNA gene promoters. Sub-figure. a-d show distribution of the feature in a sliding window of 100 bp with a step of 50 bp, resulted in 39 windows on the plot. Sub-figure. e–f show the percentage of promoters where features were found. Transparent regions correspond to 5–95% bootstrap confidence interval of the statistics. WC: word commonality, PALIN: palindromes, CGI: CpG Islands, RE: repetitive elements. The enrichment score was calculated using right-sided exact Fisher's test (Table S3). Figure I considers all protein-coding and lncRNA genes in CPS and Figure II–V shows the distribution for non-zero similarly expressed genes in cell specific manner.
(PDF)

Figure S2 **Logos for over-represented** ***ab initio*** **identified motif families (MFs) from promoters of a) protein-coding genes in CPS, b) lncRNA genes in CPS, c) protein-coding genes in REFPS and d) lncRNA genes in REFPS.** Logos for *ab initio* motif families (MFs), corresponding reverse complement (RC) MF and known TFBS match by TOMTOM system from promoters of e) lncRNA genes in CPS, f) lncRNA genes in REFPS.
(PDF)

Figure S3 **Distribution of chromatin states in cell lines with normal karyotypes across promoters of protein-coding and lncRNA genes.** Blue bar corresponds to promoters of coding genes from repeat-filtered promoter set (REFPS), green bar corresponds to promoters of coding genes from complete promoter set (CPS), red bar corresponds to promoters of lncRNAs from REFPS, and black bar corresponds to promoters of lncRNAs from CPS. This figure demonstrates fraction of all promoters overlapping with chromatin states. At the end of each bar 5–95% bootstrap confidence interval of the statistic is shown. AP: Active Promoter, WP: Weak Promoter, IP: Inactive Promoter, SE: Strong Enhancer, WE: Weak Enhancer, I: Insulator, TT: Transcriptional Transition, TE: Transcriptional Elongation, WT: Weakly Transcribed, PR: Polycomb Repressed, HC: Heterochromatin low signal, RP: Repetitive/Copy number variation.
(PDF)

Figure S4 **Distribution of histone modification marks, modified histone H2AZ, CTCF, and the Polycomb-group protein (PRC2 complex component) EZH2 in cell lines across lncRNA and protein-coding gene promoters.**
(PDF)

Figure S5 **Distribution of chromatin states in cell lines with normal karyotypes across promoters of protein-coding and lncRNA genes with similar expression.** Green bar corresponds to promoters of coding genes from complete promoter set (CPS), black bar corresponds to promoters of lncRNAs from CPS. This figure demonstrates percentage of all promoters overlapping with chromatin states. At the end of each bar 5–95% bootstrap confidence interval of the statistic is shown. AP: Active Promoter, WP: Weak Promoter, IP: Inactive Promoter, SE: Strong Enhancer, WE: Weak Enhancer, I: Insulator, TT: Transcriptional Transition, TE: Transcriptional Elongation, WT: Weakly Transcribed, PR: Polycomb Repressed, HC: Heterochromatin low signal, RP: Repetitive/Copy number variation.
(PDF)

Figure S6 **Boxplot and Quartile-Quartile plot for expression value of protein-coding genes and lncRNA genes from complete promoter set (CPS) in different cell lines.**
(PDF)

Table S1 **Mono-, di-, and tri-nucleotides frequency and observed/expected ratio for both complete promoter set (CPS) and repeat-filtered promoter set (REFPS).**
(PDF)

Table S2 **Transcription factor binding sites overrepresented in promoters of protein-coding and lncRNA genes for complete promoter set (CPS) and repeat-filtered promoter set (REFPS) and support provided by DNAseI and ChIP-seq peaks.**
(PDF)

Table S3 **P-values of overrepresentation for chromatin states, CpG islands, repetitive elements and palindromes for complete promoter set (CPS) and repeat-filtered promoter set (REFPS).**
(PDF)

Table S4 a. Summary of the results for separation of promoters of protein-coding and lncRNA genes using different combinations of features for the complete promoter set (CPS) and repeat-filtered promoter set (REFPS). For REFPS, we used all types of repeats except “simple repeats”, “low complexity regions” and “satellite repeats”. k-mer: mono-, di-,tri-nucleotide frequencies, CS: chromatin states, WC: word commonality, TFBS: transcription factor binding sites, CGI: CpG Islands, SKEW: A/T and C/G skews, PALIN: palindromes, RE: repetitive elements, COMBINE: combination of all types of features. b. Summary of the cross validation (CV) results for separation of promoters of protein-coding from lncRNA genes using all features (COMBINE) for completer promoter set (CPS) and repeat-filtered promoter set (REFPS).
(PDF)

Table S5 **Results from execution of the computational model from promoters considering only upstream ([−1000, 0]) of TSS, as well as from promoters considering only upstream ([−1000, 0]) of TSS having no overlap with CGI.**
(PDF)

Table S6 **P-values of overrepresentation for chromatin states for similarly expressed genes promoter in complete promoter set (CPS).**
(PDF)

Text S1 **Supporting information for the methods applied and results obtained.** The details of methods are described under Methods section. The details of results are described under Results section.
(DOCX)

Dataset S1 Set of a) RefSeq and b) lncRNA transcripts with hg19 human genome assembly coordinates in BED format for complete promoter set (CPS). Set of c) RefSeq and d) lncRNA transcripts with co-ordinates from hg19 in bed format for repeat-filtered promoter set (REFPS). Set of e) RefSeq and f) lncRNA promoters ([−250…+250]) with co-ordinates from hg19 in bed format for complete promoter set (CPS). Set of g) RefSeq and h) lncRNA promoters ([−250…+250]) with co-ordinates from hg19 in bed format for repeat-filtered promoter set (REFPS). i) “ENCODE_HOCOMOCO_mapping” - excel sheet contains mapping of ENCODE transcription factor name and HOCOMOCO V.8 motif name. Excel sheet “track_MEF2A_chip”, “track_GATA3_chip”, “track_ARI3A_chip” contains the track information for ENCODE ChIP-seq supported TFBS in CPS for MEF2A,GATA3 and ARI3A respectively.
(ZIP)

Dataset S2 Promoters, considering only upstream ([−1000, 0] bp), of a) RefSeq and b) lncRNA transcripts with hg19 coordinates in BED format for complete promoter set (CPS). Promoters, considering only upstream ([−1000, 0] bp), of c) RefSeq and d) lncRNA transcripts, having no overlap with CpG islands, with hg19 coordinates in BED format for CPS.
(ZIP)

Dataset S3 **RNA-seq expression value in RPKM (read per kilobase of exon per million mapped reads) for lncRNA genes and protein-coding genes in complete promoter set (CPS).**
(ZIP)
