# Supplementary material for: Promoter Analysis Reveals Globally Differential Regulation of Human Long Non-Coding RNA and Protein-Coding Genes
Source: PLoS One. 2014 Oct 2;9(10):e109443. doi: 10.1371/journal.pone.0109443 (PMC4183604; doi:10.1371/journal.pone.0109443)
Supplement: Figure S1 — DNA feature distributions in a sliding window of 100 bp with a step of 50 bp in the promoters of protein-coding and lncRNAs for complete promoter set (CPS). Green line corresponds to promoters of protein-coding genes; black line corresponds to lncRNA gene promoters. Sub-figure. a-d show distribution of the feature in a sliding window of 100 bp with a step of 50 bp, resulted in 39 windows on the plot. Sub-figure. e–f show the percentage of promoters where features were found. Transparent regions correspond to 5–95% bootstrap confidence interval of the statistics. WC: word commonality, PALIN: palindromes, CGI: CpG Islands, RE: repetitive elements. The enrichment score was calculated using right-sided exact Fisher's test (Table S3). Figure I considers all protein-coding and lncRNA genes in CPS and Figure II–V shows the distribution for non-zero similarly expressed genes in cell specific manner. (PDF) [file pone.0109443.s001.pdf]

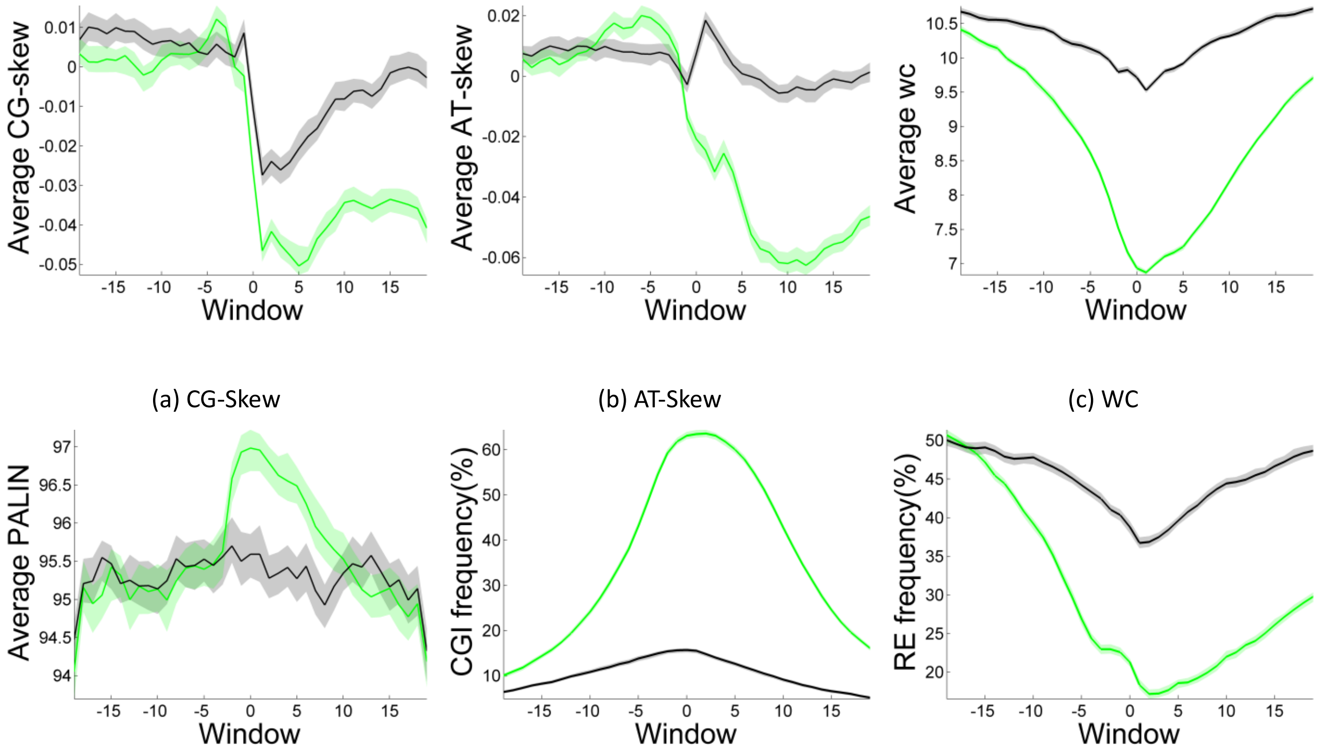

(d) Palindromes (e) CGI (f) Repeat Elements

I). DNA feature distributions in a sliding window of 100 bp with a step of 50 bp in the promoters of protein-coding and lncRNAs for complete promoter set (CPS). Green line corresponds to promoters of protein-coding genes; black line corresponds to lncRNAs gene promoters. Transparent regions correspond to 5-95% bootstrap confidence interval.

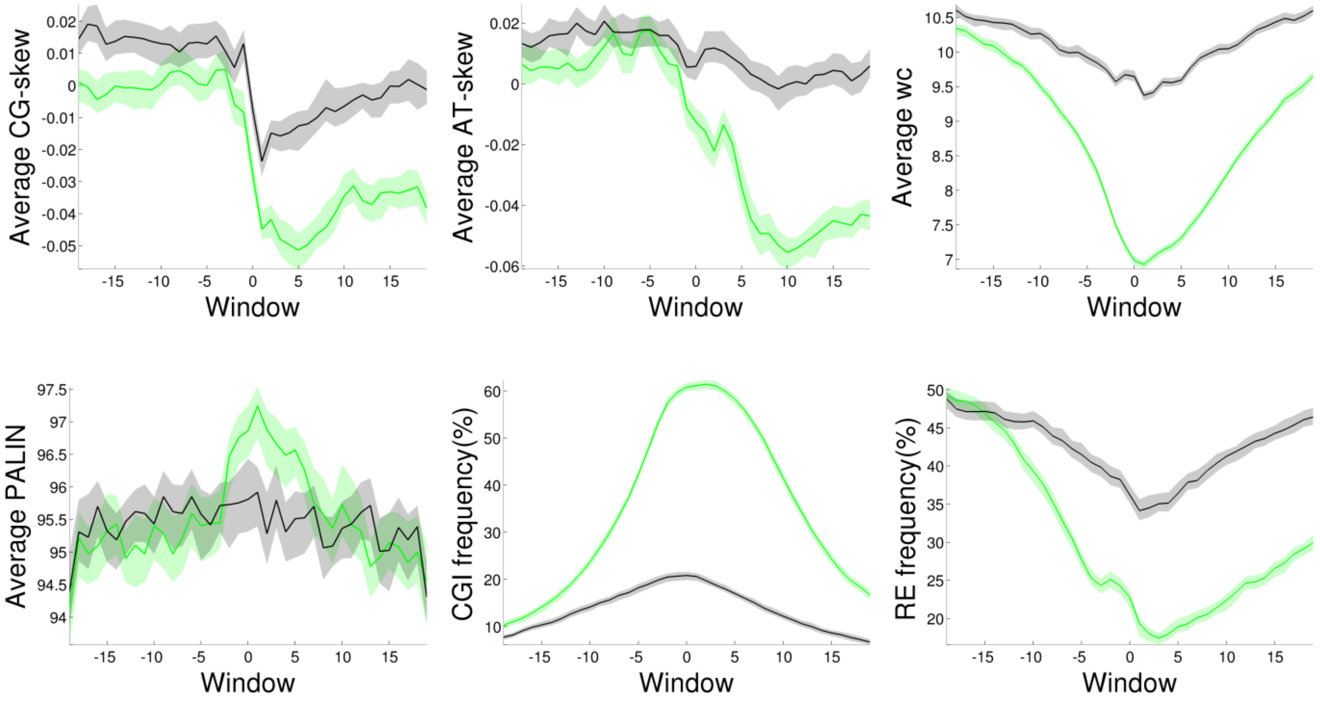

II) DNA feature distributions in a sliding window of 100 bp with a step of 50 bp in the promoters of non-zero similarly expressed protein-coding genes and lncRNA genes in cell line Gm12878.

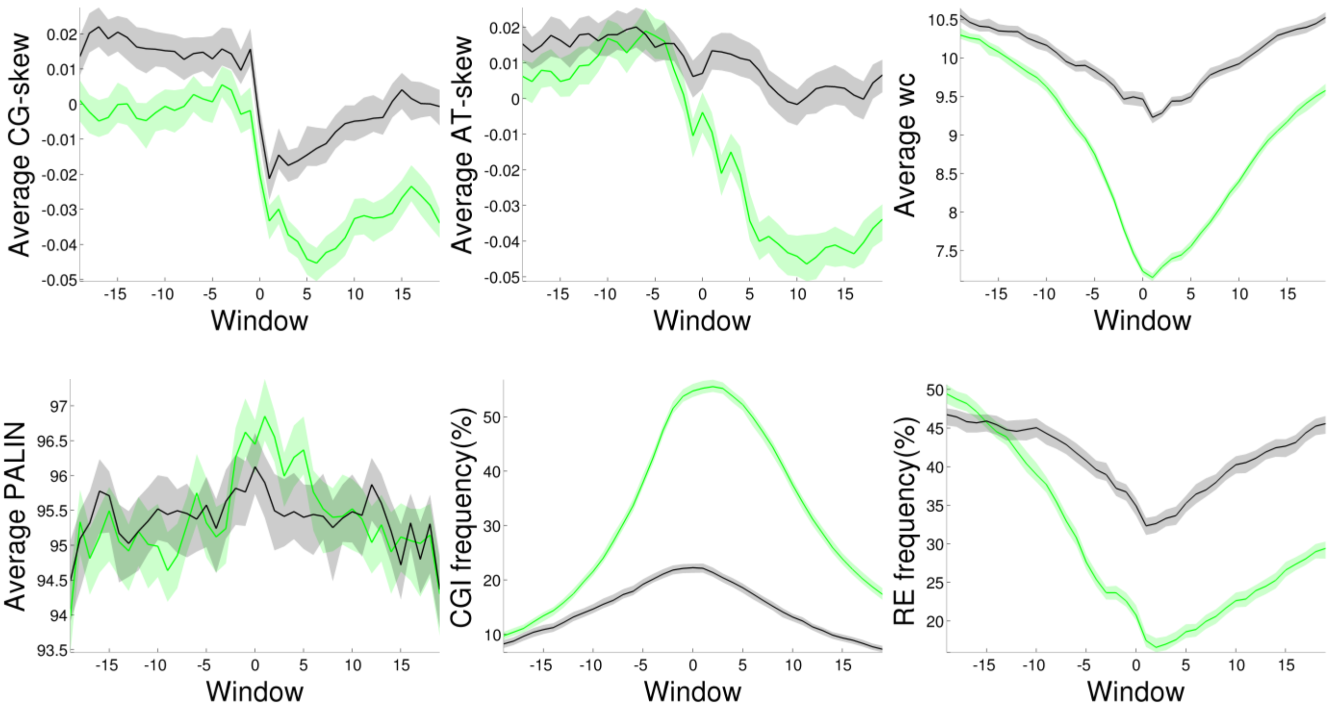

III) DNA feature distributions in a sliding window of 100 bp with a step of 50 bp in the promoters of non-zero similarly expressed protein-coding genes and lncRNA genes in cell line H1-hESC

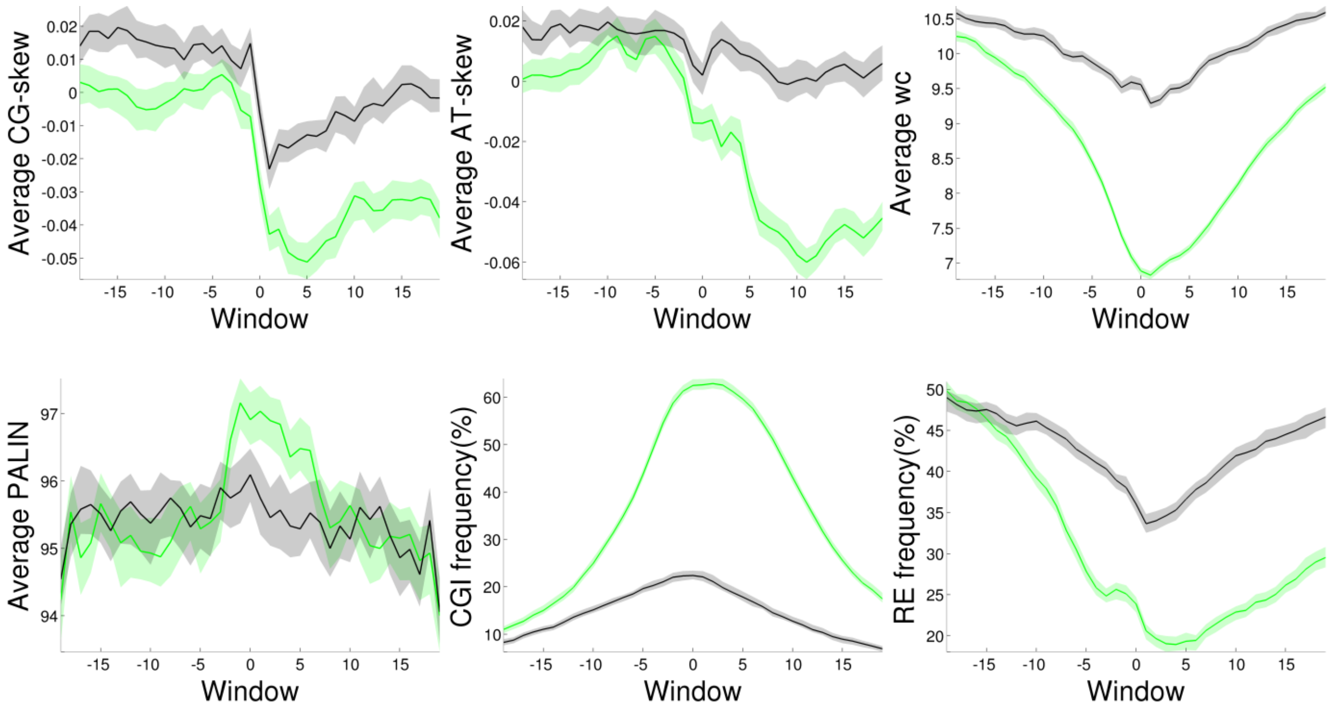

IV) DNA feature distributions in a sliding window of 100 bp with a step of 50 bp in the promoters of non-zero similarly expressed protein-coding genes and lncRNA genes in cell line K562

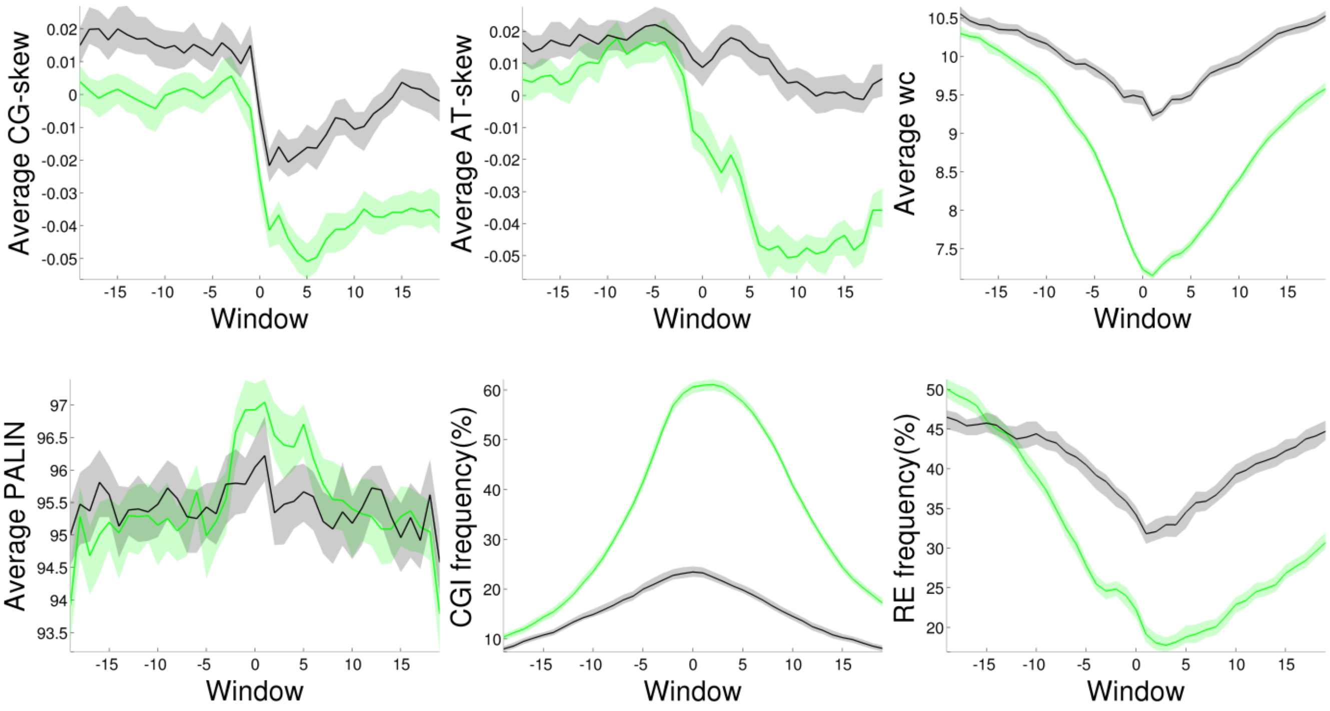

V) DNA feature distributions in a sliding window of 100 bp with a step of 50 bp in the promoters of non-zero similarly expressed protein-coding genes and lncRNA genes in cell line HUVEC

Fig. S1. DNA feature distributions in a sliding window of 100 bp with a step of 50 bp in the promoters of protein-coding and lncRNAs for complete promoter set (CPS). Green line corresponds to promoters of protein-coding genes; black line corresponds to lncRNAs gene promoters. Transparent regions correspond to 5-95% bootstrap confidence interval. Figure I considers all protein-coding and lncRNA genes in CPS and Figure II- V shows the distribution for non-zero similarly expressed genes in cell specific manner.
