# Supplementary material for: Promoter Analysis Reveals Globally Differential Regulation of Human Long Non-Coding RNA and Protein-Coding Genes
Source: PLoS One. 2014 Oct 2;9(10):e109443. doi: 10.1371/journal.pone.0109443 (PMC4183604; doi:10.1371/journal.pone.0109443)
Supplement: Figure S2 — Logos for over-represented ab initio identified motif families (MFs) from promoters of a) protein-coding genes in CPS, b) lncRNA genes in CPS, c) protein-coding genes in REFPS and d) lncRNA genes in REFPS. Logos for ab initio motif families (MFs), corresponding reverse complement (RC) MF and known TFBS match by TOMTOM system from promoters of e) lncRNA genes in CPS, f) lncRNA genes in REFPS. (PDF) [file pone.0109443.s002.pdf]

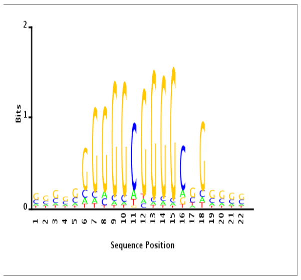

DMF191

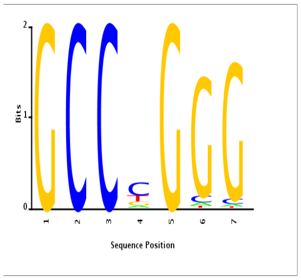

DMF840

a: Logos for over-represented *ab initio* motifs from promoters of protein-coding genes in complete promoter set(CPS)

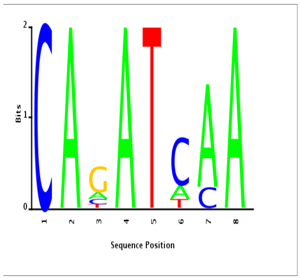

DMF150

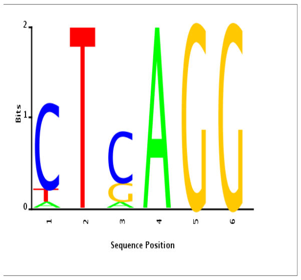

DMF267

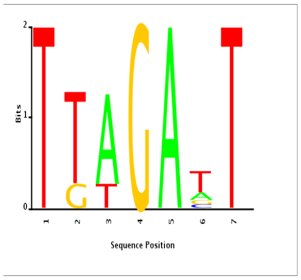

DMF187

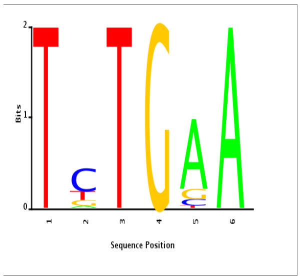

DMF274

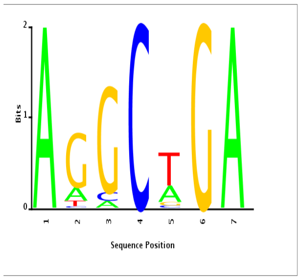

DMF217

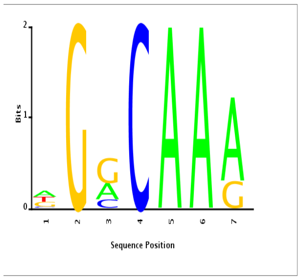

DMF266

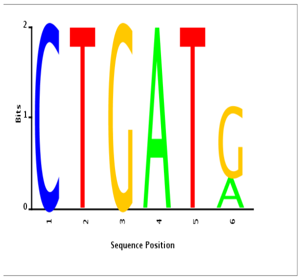

DMF251

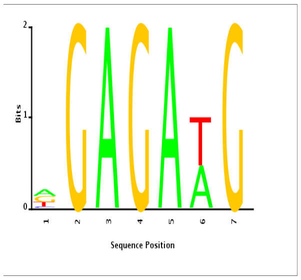

DMF253

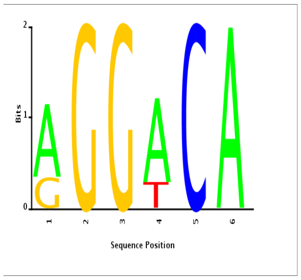

DMF255

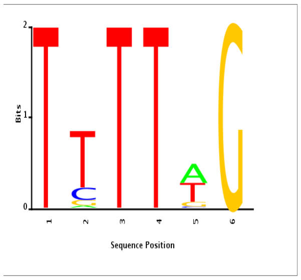

DMF273

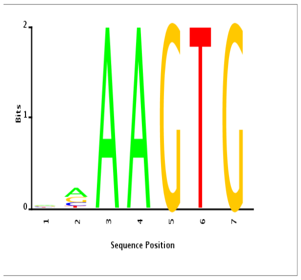

DMF204

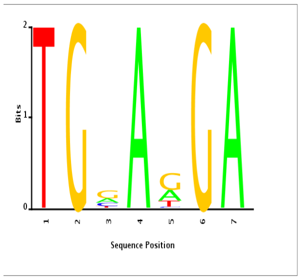

DMF220

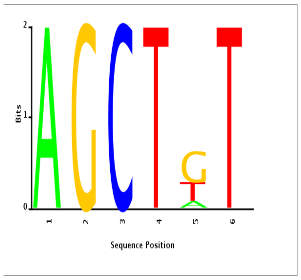

DMF256

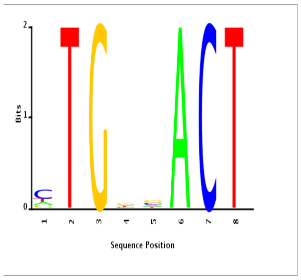

DMF163

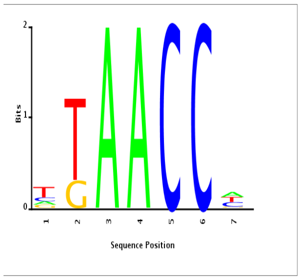

DMF257

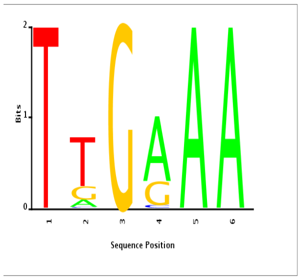

DMF263

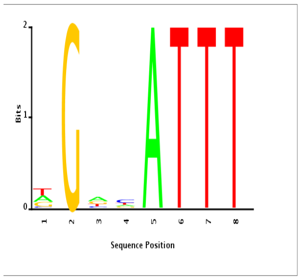

DMF222

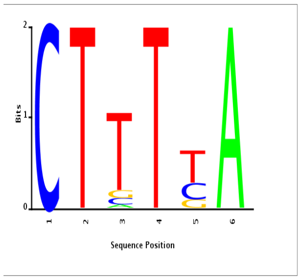

DMF282

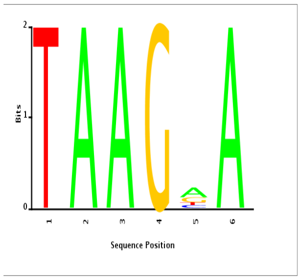

DMF235

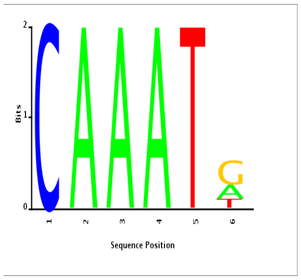

DMF250

b: Logos for over-represented *ab initio* motifs from promoters of lncRNA genes in complete promoter set(CPS) (cont..)

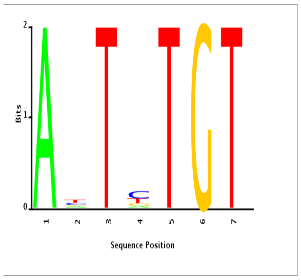

DMF226

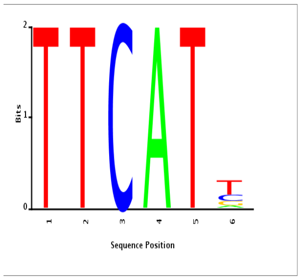

DMF243

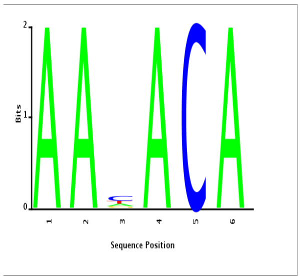

DMF265

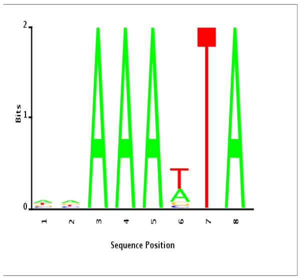

DMF275

b: Logos for over-represented *ab initio* motifs from promoters of lncRNA genes in complete promoter set(CPS)

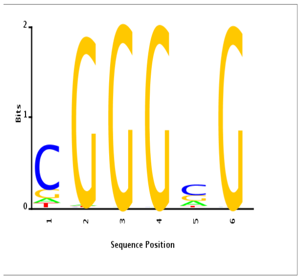

DMF980

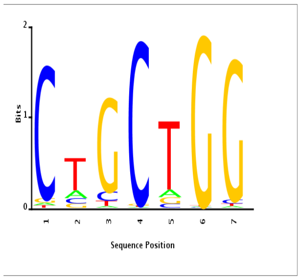

DMF887

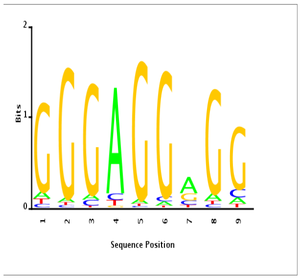

DMF677

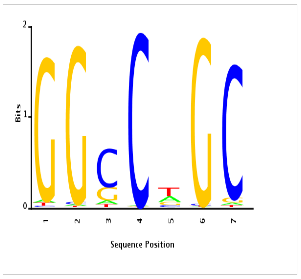

DMF841

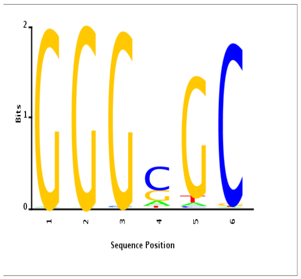

DMF964

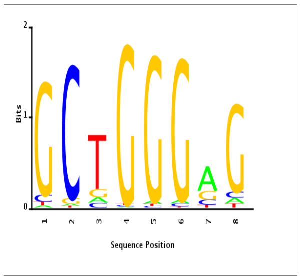

DMF745

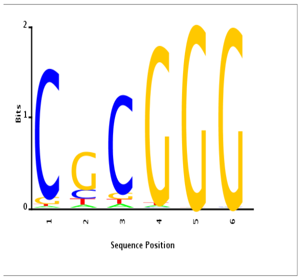

DMF945

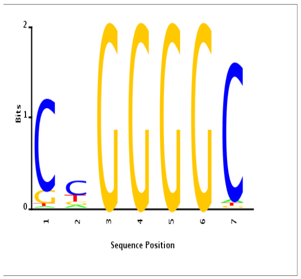

DMF834

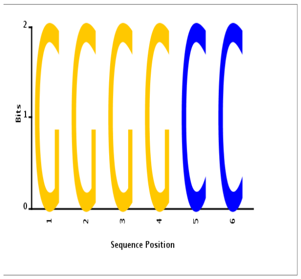

DMF891

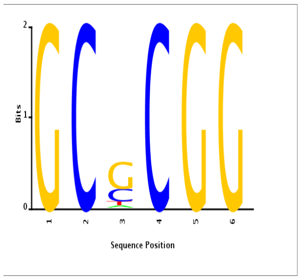

DMF932

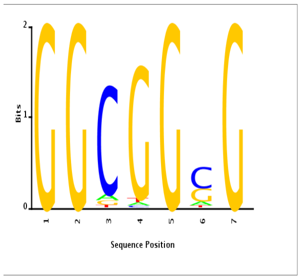

DMF816

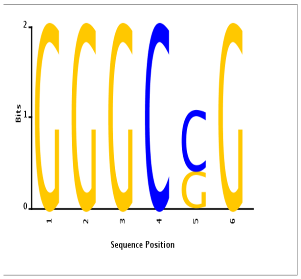

DMF907

c: Logos for over-represented *ab initio* motifs from promoters of protein-coding genes in repeat-filtered promoter set(REFPS)

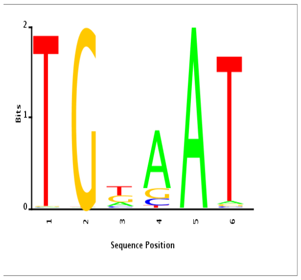

DMF985

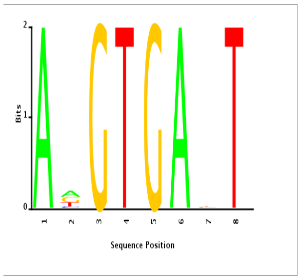

DMF710

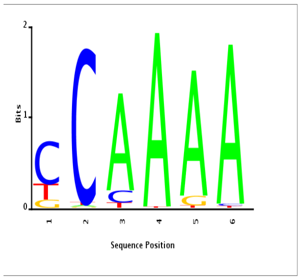

DMF953

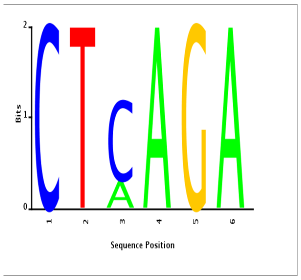

DMF923

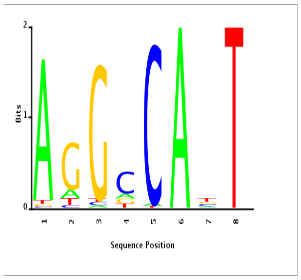

DMF730

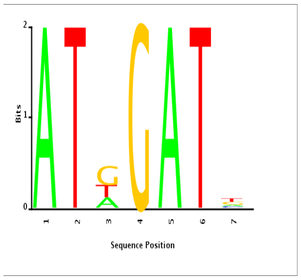

DMF808

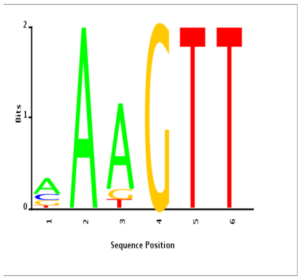

DMF942

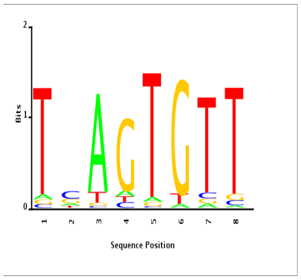

DMF760

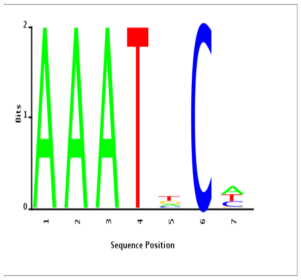

DMF940

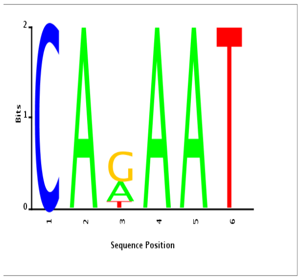

DMF909

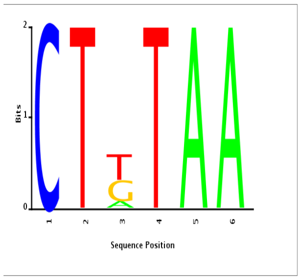

DMF914

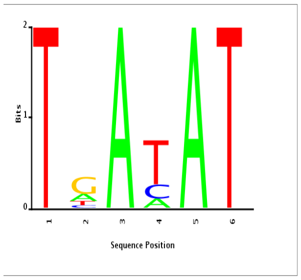

DMF943

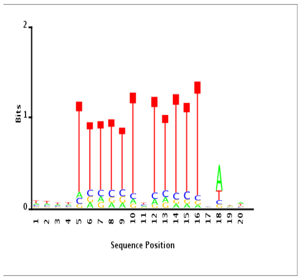

DMF194

d: Logos for over-represented *ab initio* motifs from promoters of lncRNA genes in repeat-filtered promoter set(REFPS)

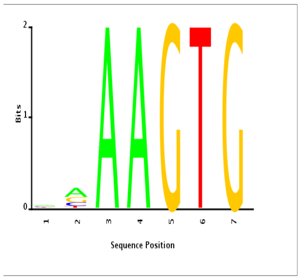

DMF204

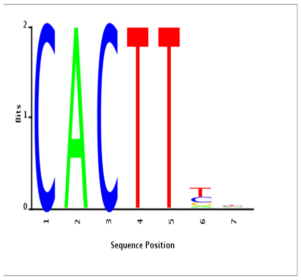

DMF204_RC

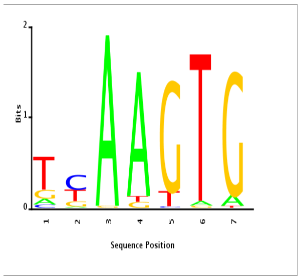

NKX25_HUMAN

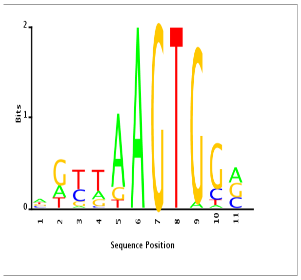

NKX32_HUMAN

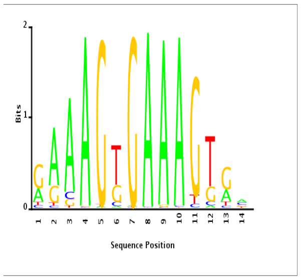

PRDM1_HUMAN

e: Known TFBS match with *ab initio* motifs from promoters of long non-coding RNA(lncRNA) genes in complete promoter set(CPS)

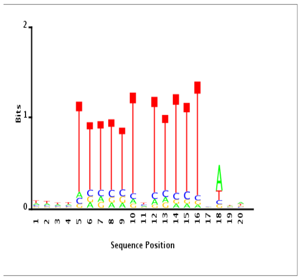

DMF194

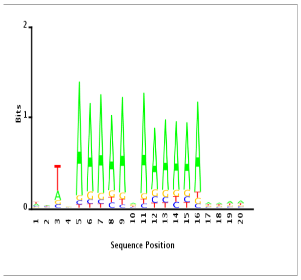

DMF194_RC

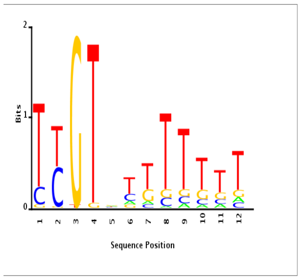

FUBP1_HUMAN

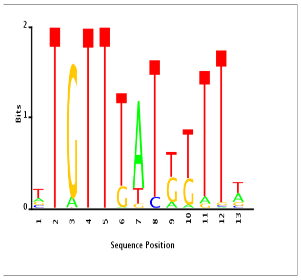

FOXJ3_HUMAN

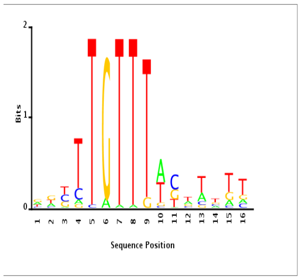

FOXO1_HUMAN

---

f: Known TFBS match with *ab initio* motifs from promoters of long non-coding RNA(lncRNA) genes in repeat-filtered promoter set(REFPS)

Fig. S2. Logos for over-represented *ab initio* motif families (MFs) from promoters of: a) protein-coding genes in CPS b) lncRNA genes in CPS c) protein-coding genes in REFPS d) lncRNA genes in REFPS. Logos for *ab initio* motif families (MFs), corresponding reverse complement(RC) MF and known TFBS match by TOMTOM system from promoters of: e) lncRNA genes in CPS f) lncRNA genes in REFPS.
