# Supplementary material for: Promoter Analysis Reveals Globally Differential Regulation of Human Long Non-Coding RNA and Protein-Coding Genes
Source: PLoS One. 2014 Oct 2;9(10):e109443. doi: 10.1371/journal.pone.0109443 (PMC4183604; doi:10.1371/journal.pone.0109443)
Supplement: Figure S3 — Distribution of chromatin states in cell lines with normal karyotypes across promoters of protein-coding and lncRNA genes. Blue bar corresponds to promoters of coding genes from repeat-filtered promoter set (REFPS), green bar corresponds to promoters of coding genes from complete promoter set (CPS), red bar corresponds to promoters of lncRNAs from REFPS, and black bar corresponds to promoters of lncRNAs from CPS. This figure demonstrates fraction of all promoters overlapping with chromatin states. At the end of each bar 5–95% bootstrap confidence interval of the statistic is shown. AP: Active Promoter, WP: Weak Promoter, IP: Inactive Promoter, SE: Strong Enhancer, WE: Weak Enhancer, I: Insulator, TT: Transcriptional Transition, TE: Transcriptional Elongation, WT: Weakly Transcribed, PR: Polycomb Repressed, HC: Heterochromatin low signal, RP: Repetitive/Copy number variation. (PDF) [file pone.0109443.s003.pdf]

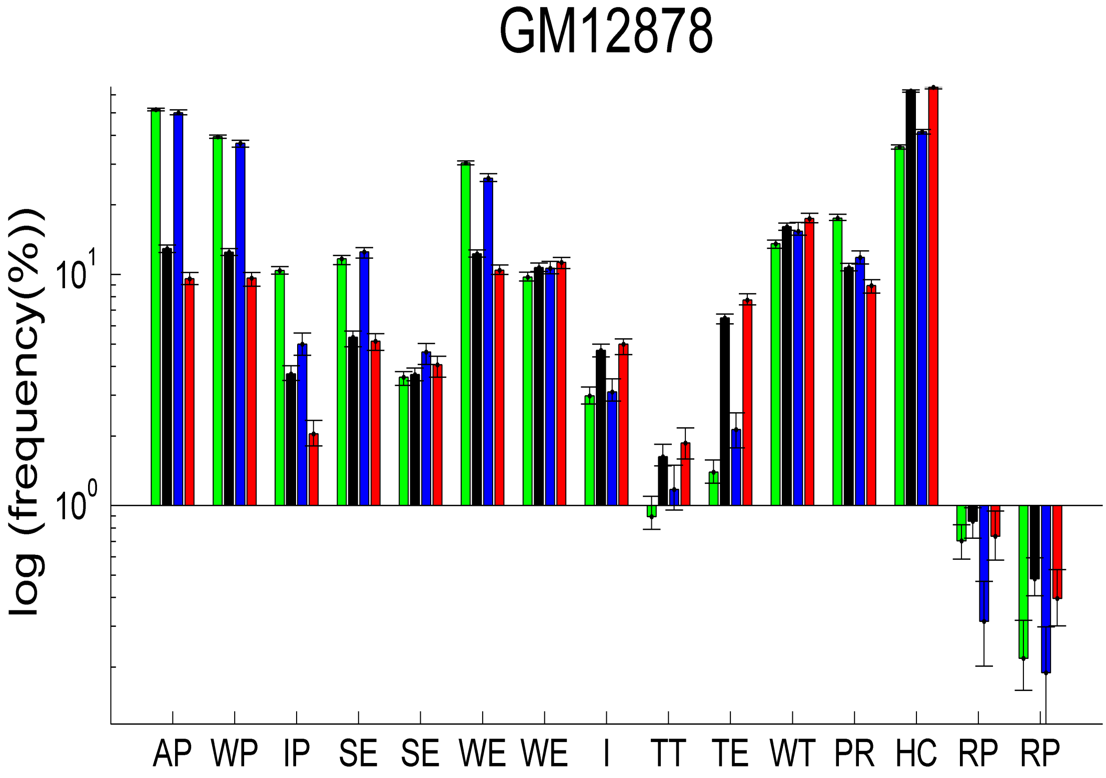

(a) Distribution of chromatin states in GM12878 with normal karyotypes across promoters of protein-coding and lncRNA genes.

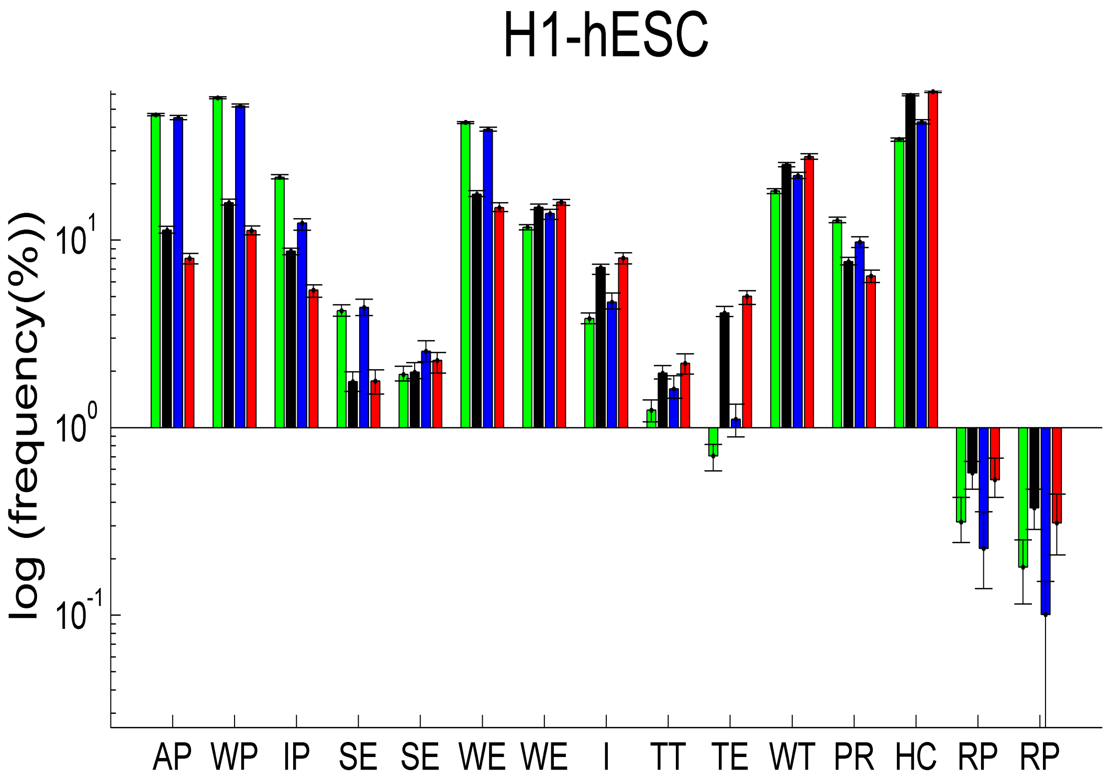

(b) Distribution of chromatin states in H1-hESC with normal karyotypes across promoters of protein-coding and lncRNA genes.

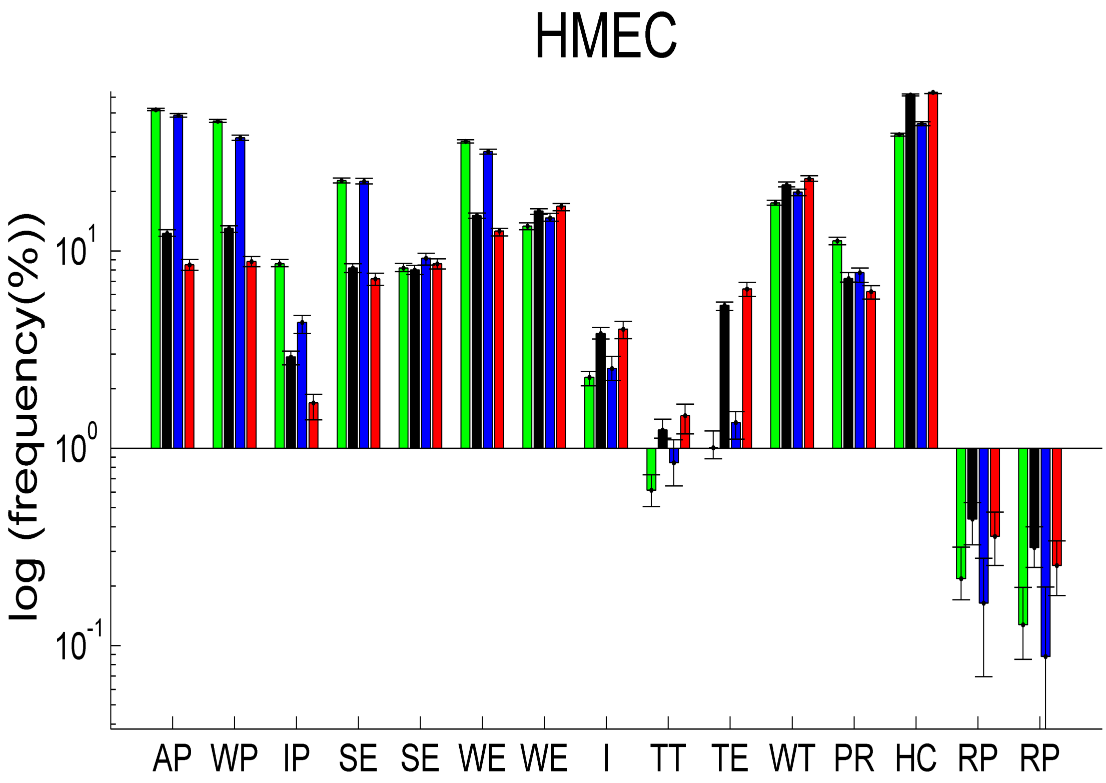

(c) Distribution of chromatin states in HMEC with normal karyotypes across promoters of protein-coding and lncRNA genes.

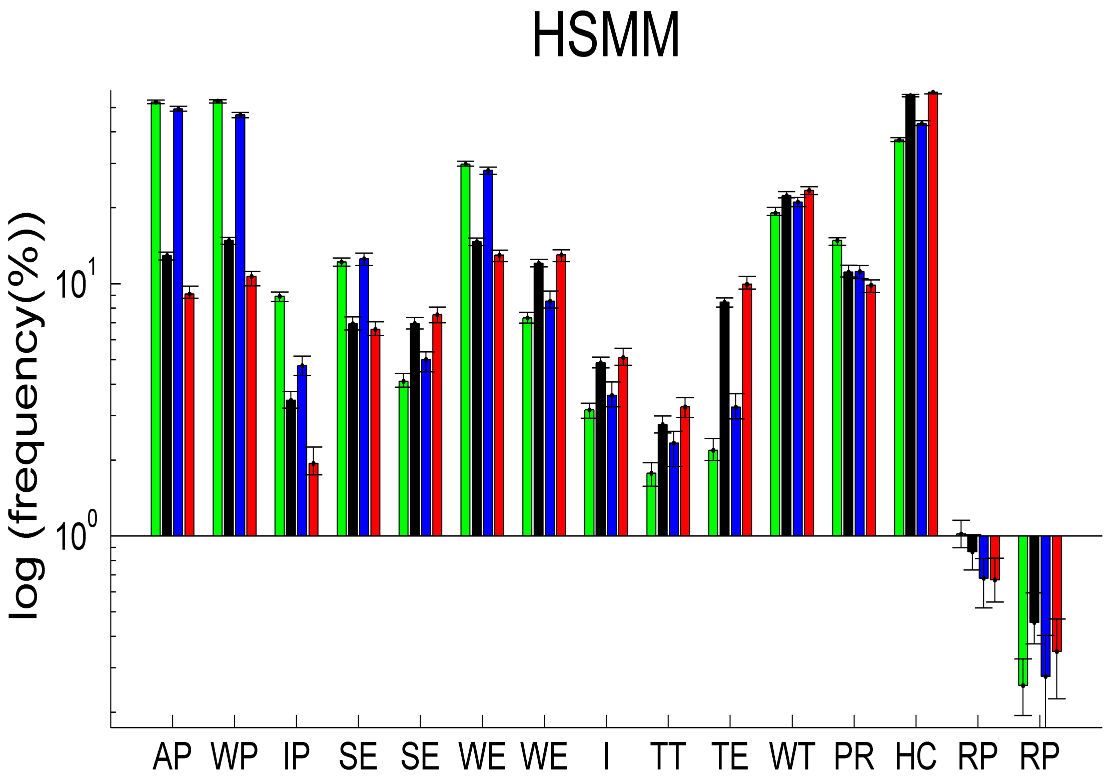

(d) Distribution of chromatin states in HSMM with normal karyotypes across promoters of protein-coding and lncRNA genes.

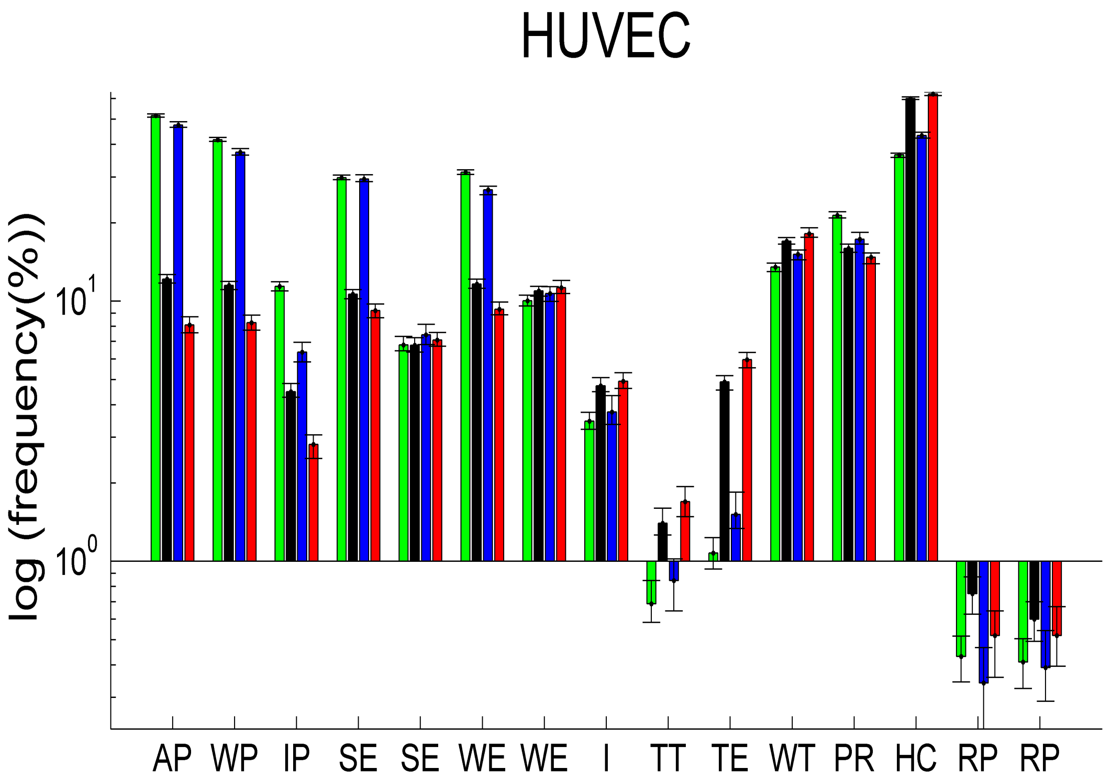

(e) Distribution of chromatin states in HUVEC with normal karyotypes across promoters of protein-coding and lncRNA genes.

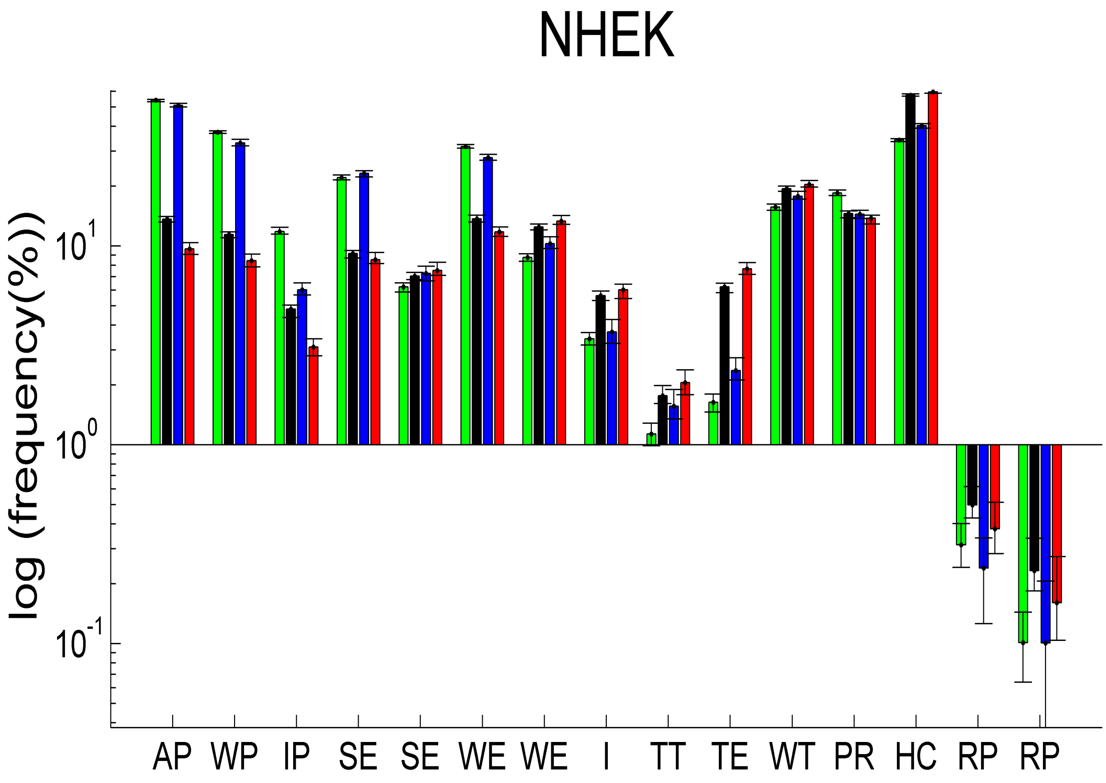

(f) Distribution of chromatin states in NHEK with normal karyotypes across promoters of protein-coding and lncRNA genes.

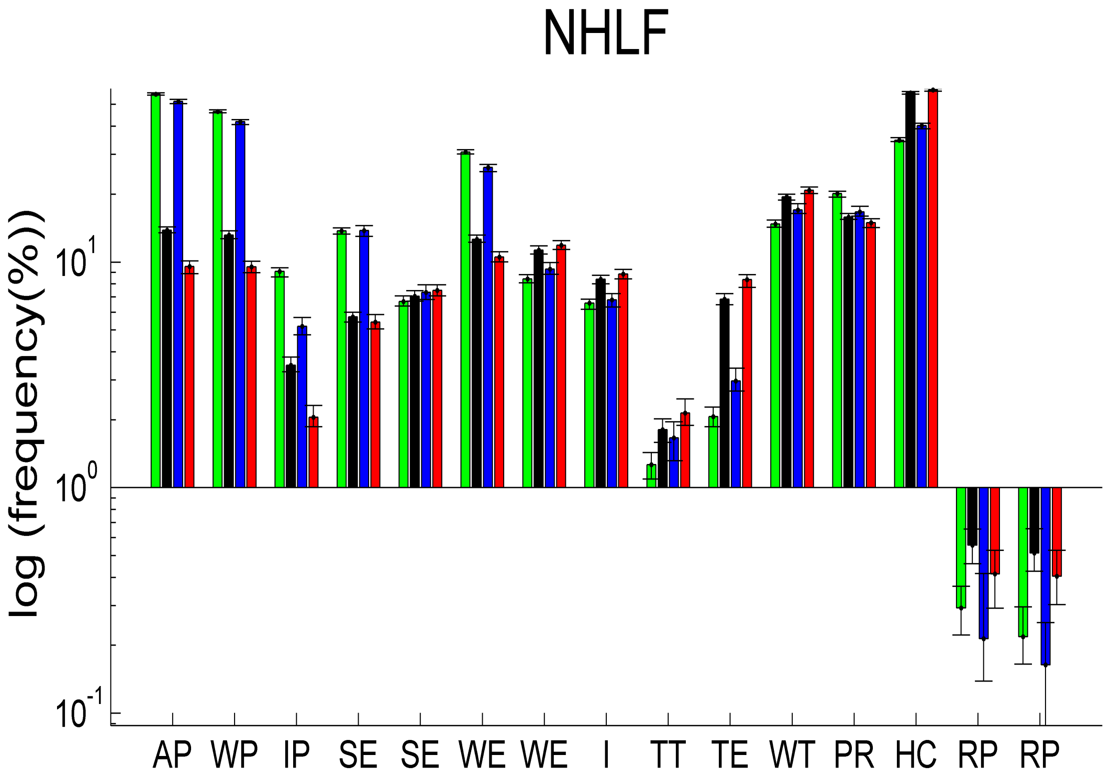

(g) Distribution of chromatin states in NHLF with normal karyotypes across promoters of protein-coding and lncRNA genes.

Fig. S3. Distribution of chromatin states in cell lines with normal karyotypes across promoters of protein-coding and lncRNA genes. Blue bar corresponds to promoters of coding genes from repeat-filtered promoter set (REFPS), green bar corresponds to promoters of coding genes from complete promoter set (CPS) , red bar corresponds to promoters of lncRNAs from REFPS, black bar corresponds to promoters of lncRNAs from CPS. This figure demonstrates percentage of all promoters overlapping with chromatin states. At the end of each bar 5-95% bootstrap confidence interval of the statistic is shown. AP: Active Promoter, WP: Weak Promoter, IP: Inactive Promoter,SE: Strong Enhancer, WE: Weak Enhancer, I: Insulator, TT: Transcriptional Transition, TE: Transcriptional Elongation, WT: Weakly Transcribed, PR: Polycomb Repressed, HC: Heterochromatin low signal, RP: Repetitive/Copy number variation.Details of fifteen states and the variation in two SE, WE, RP states are described in SI [11].
