# Supplementary material for: Promoter Analysis Reveals Globally Differential Regulation of Human Long Non-Coding RNA and Protein-Coding Genes
Source: PLoS One. 2014 Oct 2;9(10):e109443. doi: 10.1371/journal.pone.0109443 (PMC4183604; doi:10.1371/journal.pone.0109443)
Supplement: Figure S4 — Distribution of histone modification marks, modified histone H2AZ, CTCF, and the Polycomb-group protein (PRC2 complex component) EZH2 in cell lines across lncRNA and protein-coding gene promoters. (PDF) [file pone.0109443.s004.pdf]

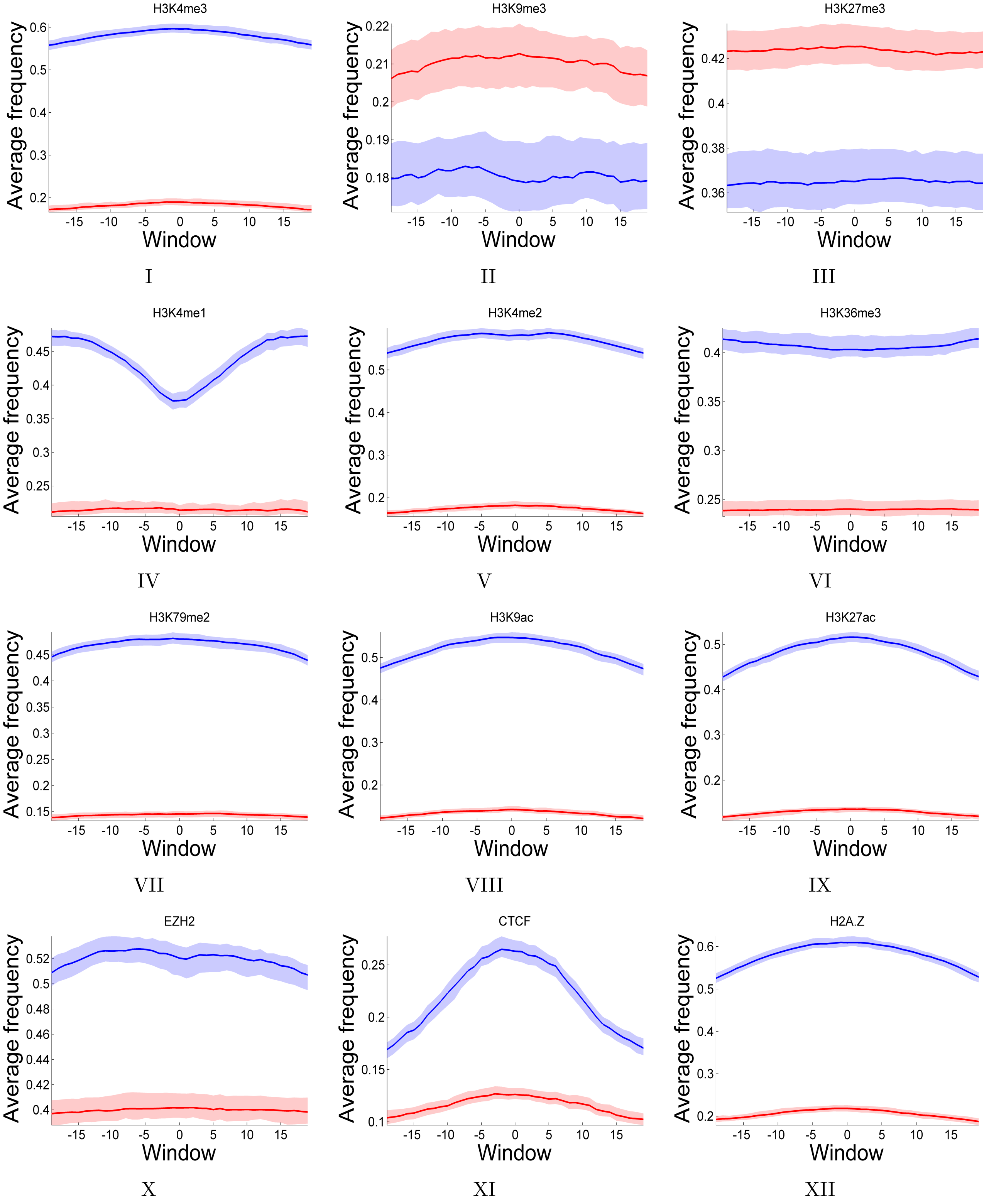

a) Distribution of histone modification marks, modified histone H2A.Z, CTCF, and the Polycomb-group protein (PRC2 complex component) EZH2 in GM12878 cell line across lncRNA and protein-coding gene promoters. Figure demonstrates fraction of all promoters overlapping with chromatin at particular mark. Blue line corresponds to promoters of coding genes from repeat-filtered promoter set (REFPS), red line corresponds to promoters of lncRNAs from REFPS. Transparent regions correspond to 5-95% bootstrap confidence interval of the statistic.

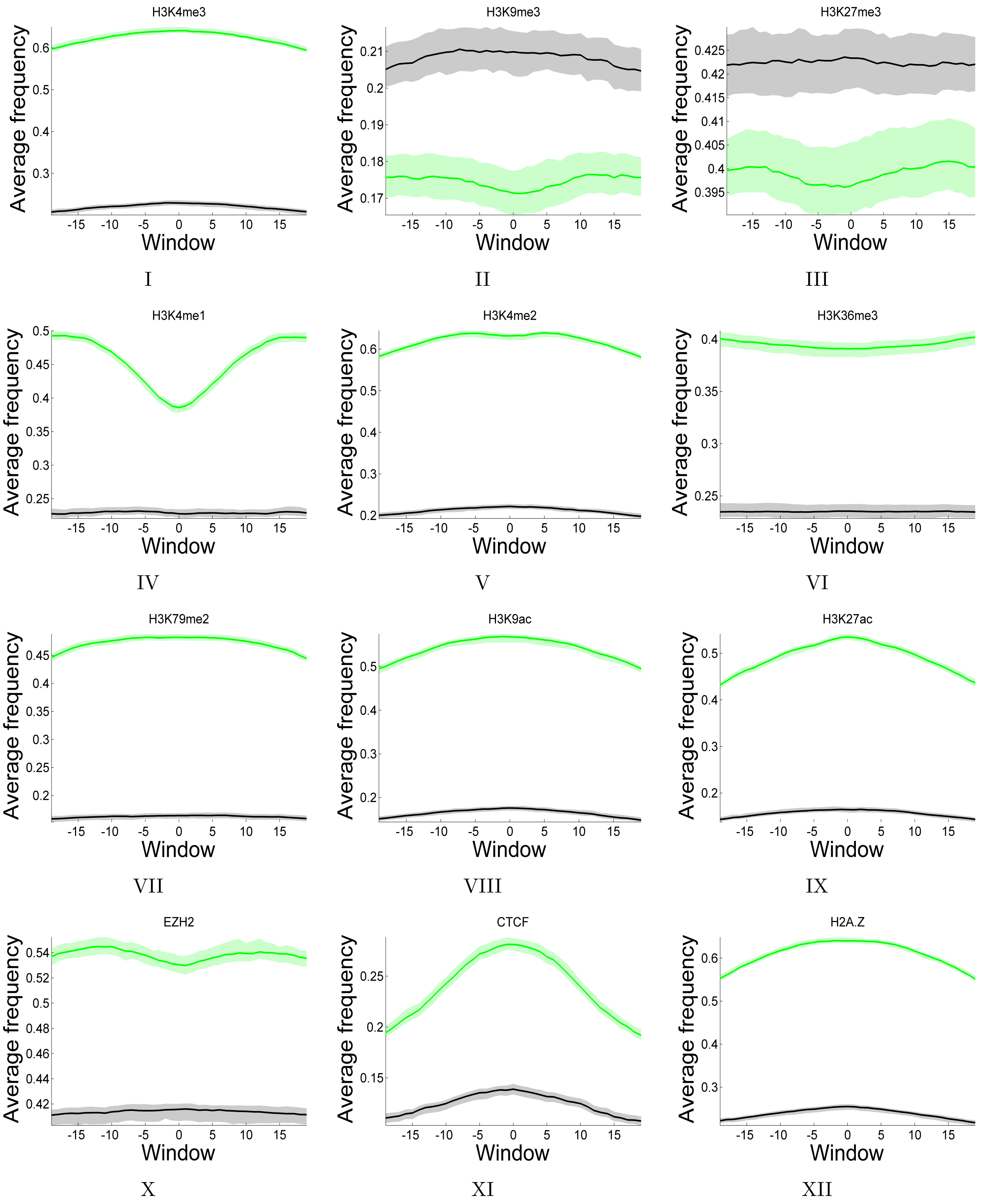

b) Distribution of histone modification marks, modified histone H2A.Z, CTCF, and the Polycomb-group protein (PRC2 complex component) EZH2 in GM12878 cell line across lncRNA and protein-coding gene promoters. Figure demonstrates fraction of all promoters overlapping with chromatin at particular mark. Green line corresponds to promoters of coding genes from complete promoter set (CPS), black line corresponds to promoters of lncRNAs from CPS. Transparent regions correspond to 5-95% bootstrap confidence interval of the statistics.

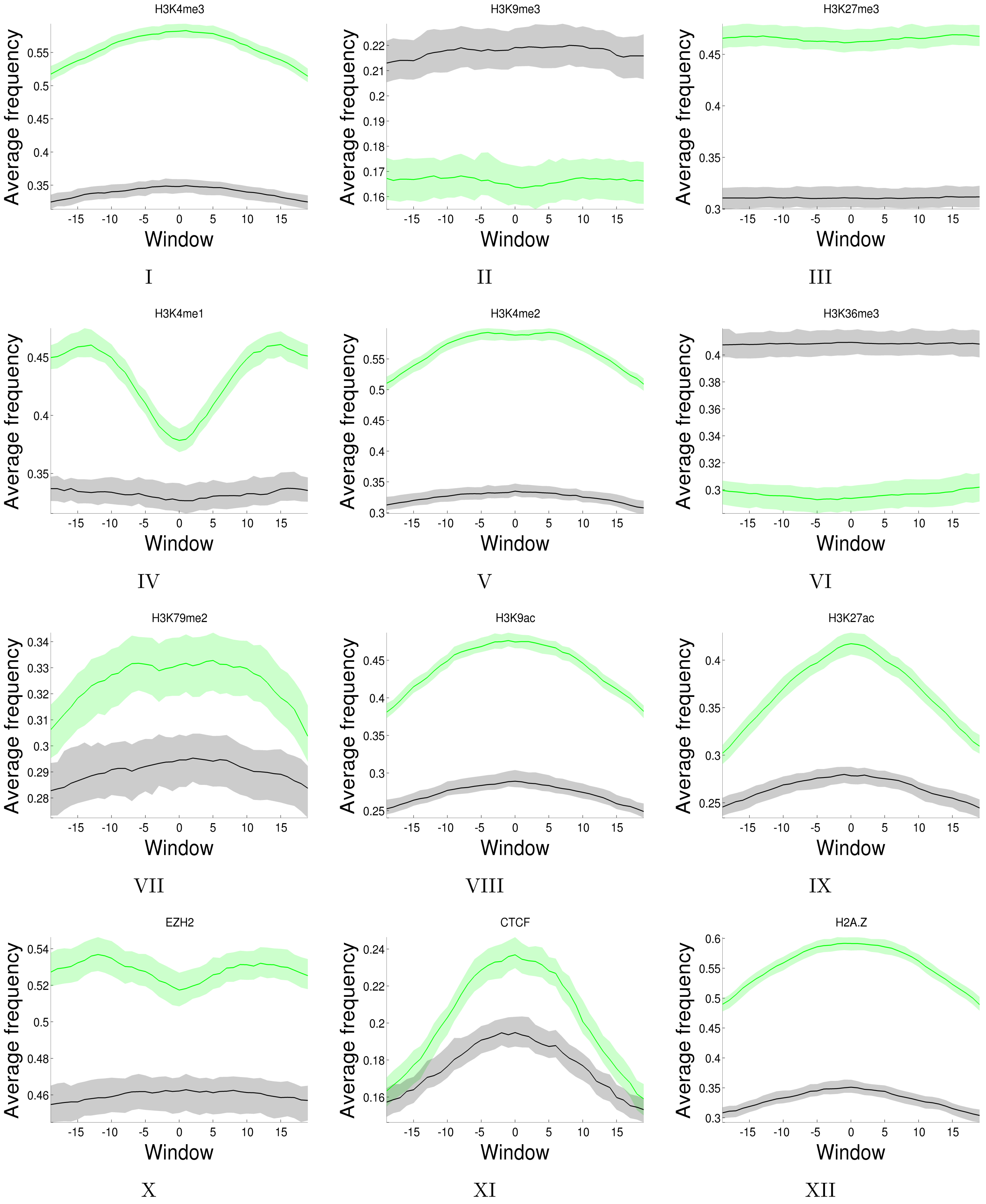

c) Distribution of histone modification marks, modified histone H2A.Z, CTCF, and the Polycomb-group protein (PRC2 complex component) EZH2 in GM12878 cell line across lncRNA and protein-coding gene promoters with similar expression. Figure demonstrates fraction of all promoters overlapping with chromatin at particular mark.

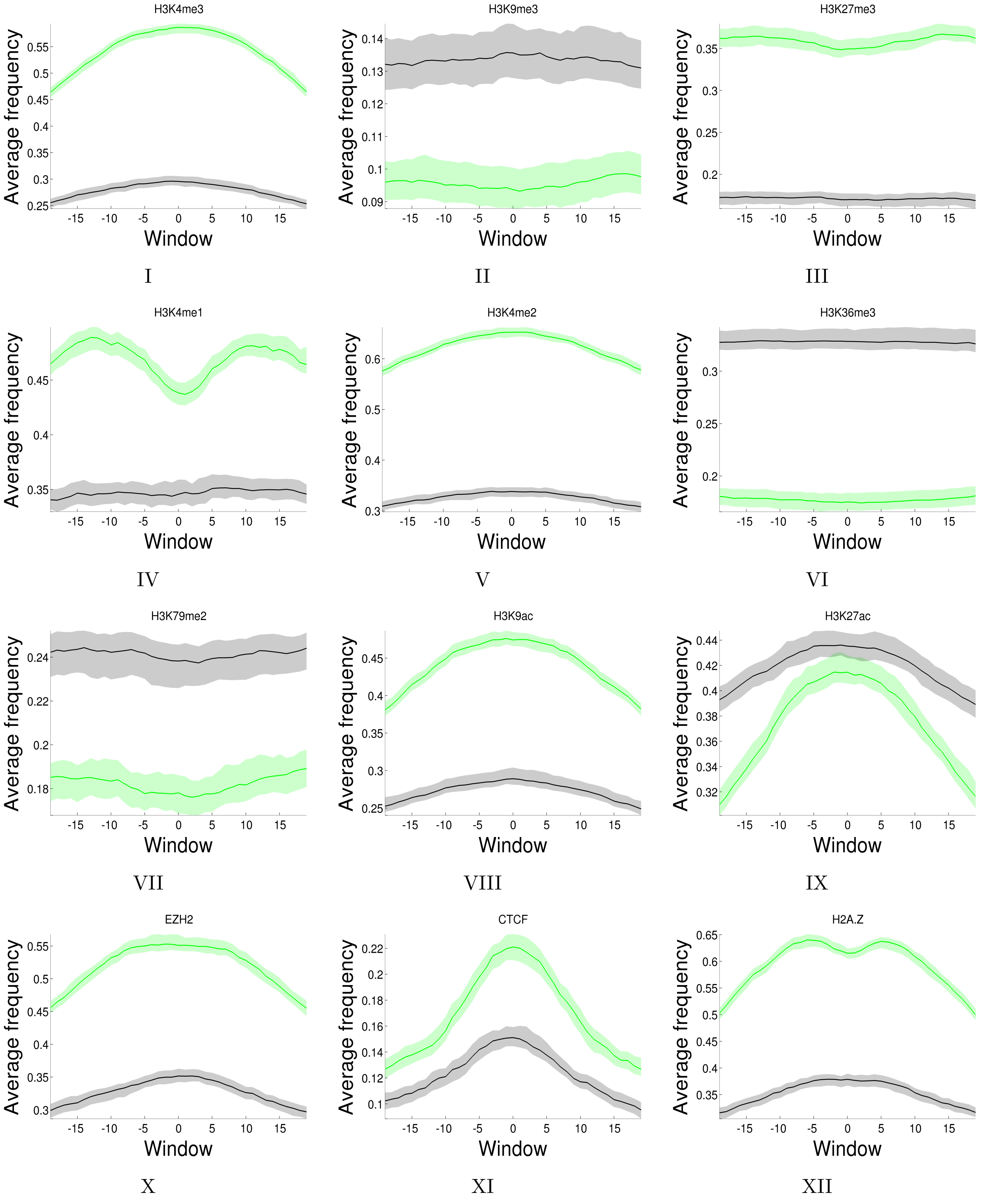

d) Distribution of histone modification marks, modified histone H2A.Z, CTCF, and the Polycomb-group protein (PRC2 complex component) EZH2 in H1HESC cell line across lncRNA and protein-coding gene promoters with similar expression. Figure demonstrates fraction of all promoters overlapping with chromatin at particular mark.

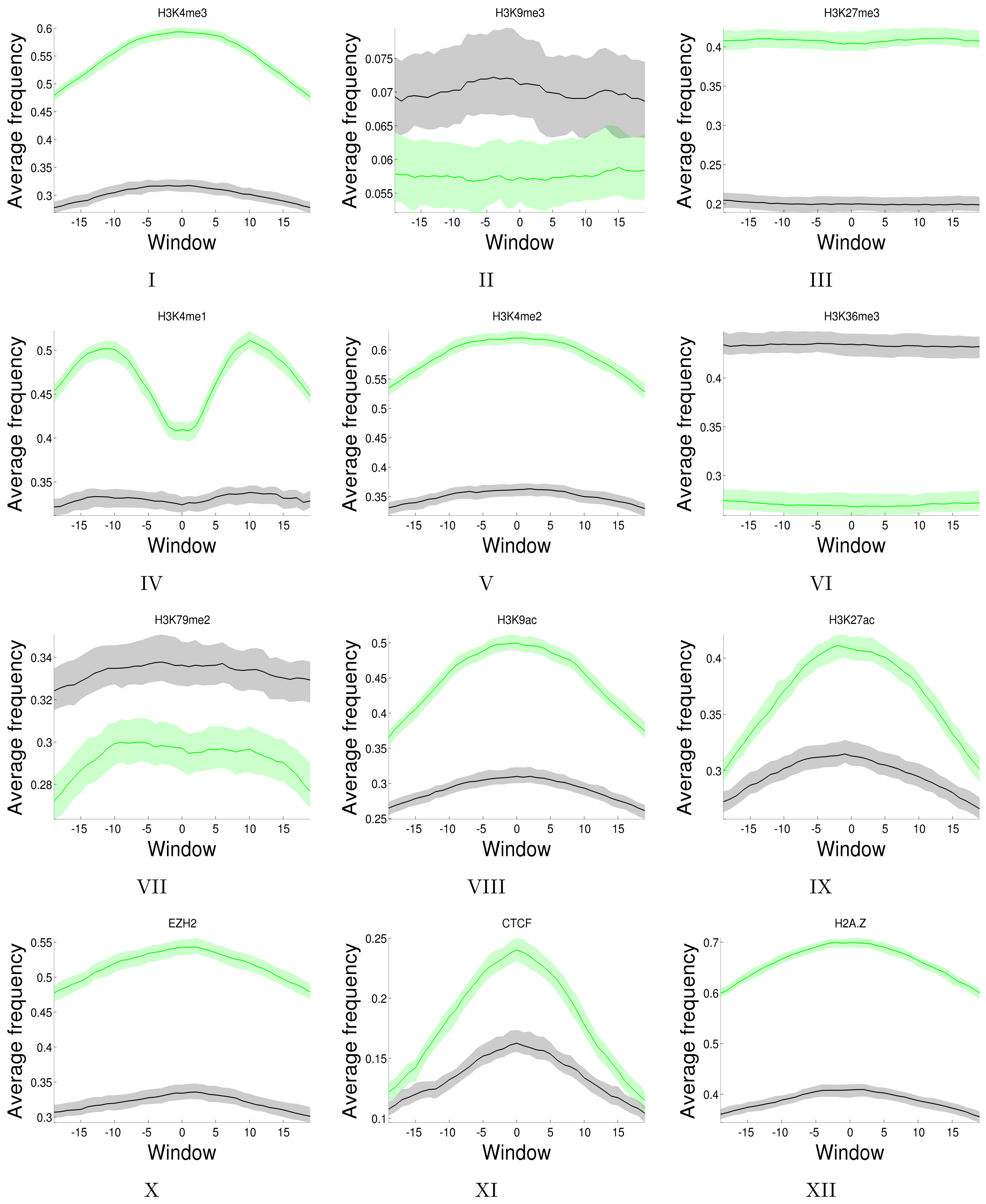

e) Distribution of histone modification marks, modified histone H2A.Z, CTCF, and the Polycomb-group protein (PRC2 complex component) EZH2 in HUVEC cell line across lncRNA and protein-coding gene promoters with similar expression. Figure demonstrates fraction of all promoters overlapping with chromatin at particular mark.

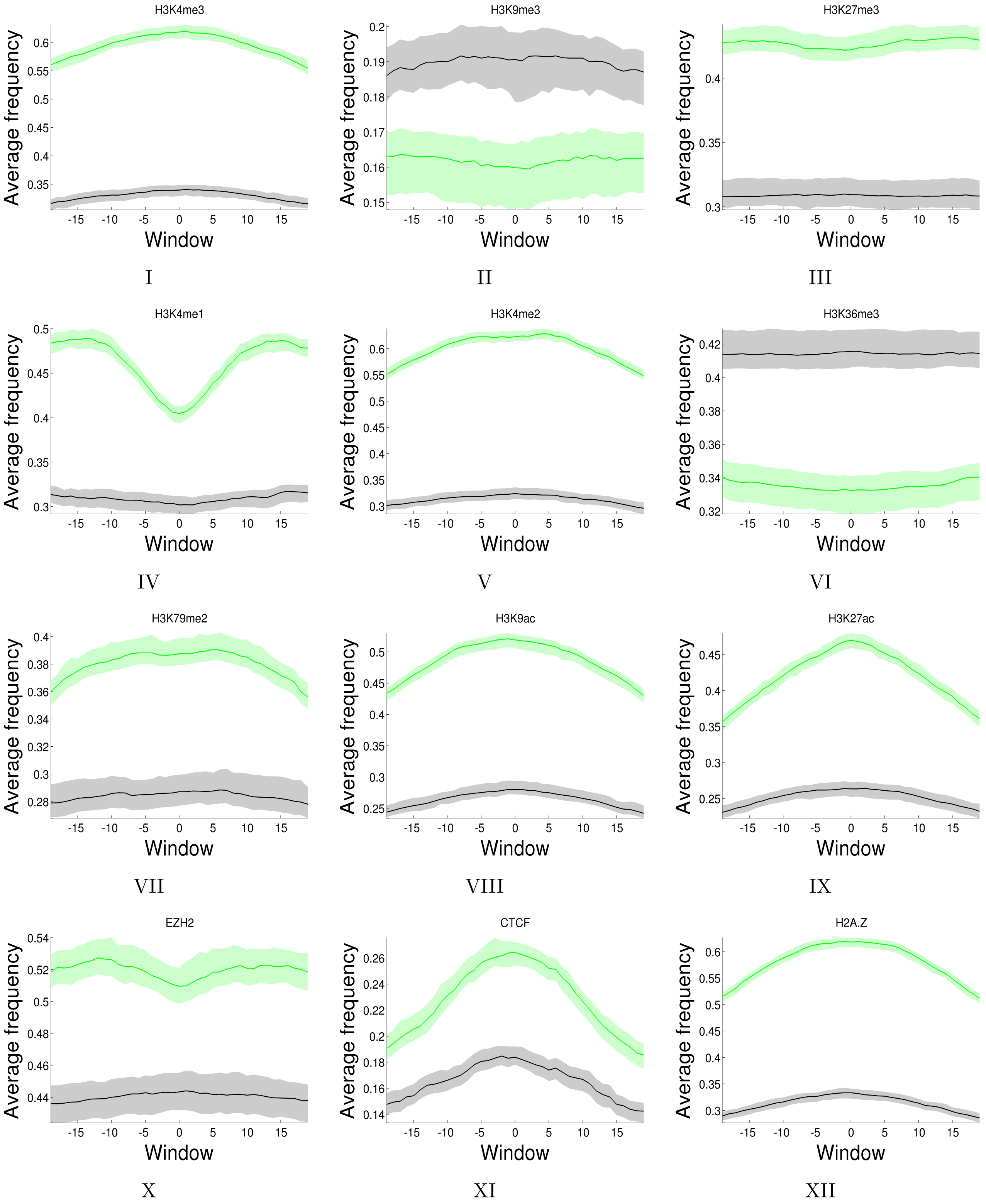

f) Distribution of histone modification marks, modified histone H2A.Z, CTCF, and the Polycomb-group protein (PRC2 complex component) EZH2 in K562 cell line across lncRNA and protein-coding gene promoters with similar expression. Figure demonstrates fraction of all promoters overlapping with chromatin at particular mark.

Fig. S4 Distribution of histone modification marks, modified histone H2A.Z, CTCF, and the Polycomb-group protein (PRC2 complex component) EZH2 in cell lines across lncRNA and protein-coding gene promoters.
