# Supplementary material for: Promoter Analysis Reveals Globally Differential Regulation of Human Long Non-Coding RNA and Protein-Coding Genes
Source: PLoS One. 2014 Oct 2;9(10):e109443. doi: 10.1371/journal.pone.0109443 (PMC4183604; doi:10.1371/journal.pone.0109443)
Supplement: Figure S5 — Distribution of chromatin states in cell lines with normal karyotypes across promoters of protein-coding and lncRNA genes with similar expression. Green bar corresponds to promoters of coding genes from complete promoter set (CPS), black bar corresponds to promoters of lncRNAs from CPS. This figure demonstrates percentage of all promoters overlapping with chromatin states. At the end of each bar 5–95% bootstrap confidence interval of the statistic is shown. AP: Active Promoter, WP: Weak Promoter, IP: Inactive Promoter, SE: Strong Enhancer, WE: Weak Enhancer, I: Insulator, TT: Transcriptional Transition, TE: Transcriptional Elongation, WT: Weakly Transcribed, PR: Polycomb Repressed, HC: Heterochromatin low signal, RP: Repetitive/Copy number variation. (PDF) [file pone.0109443.s005.pdf]

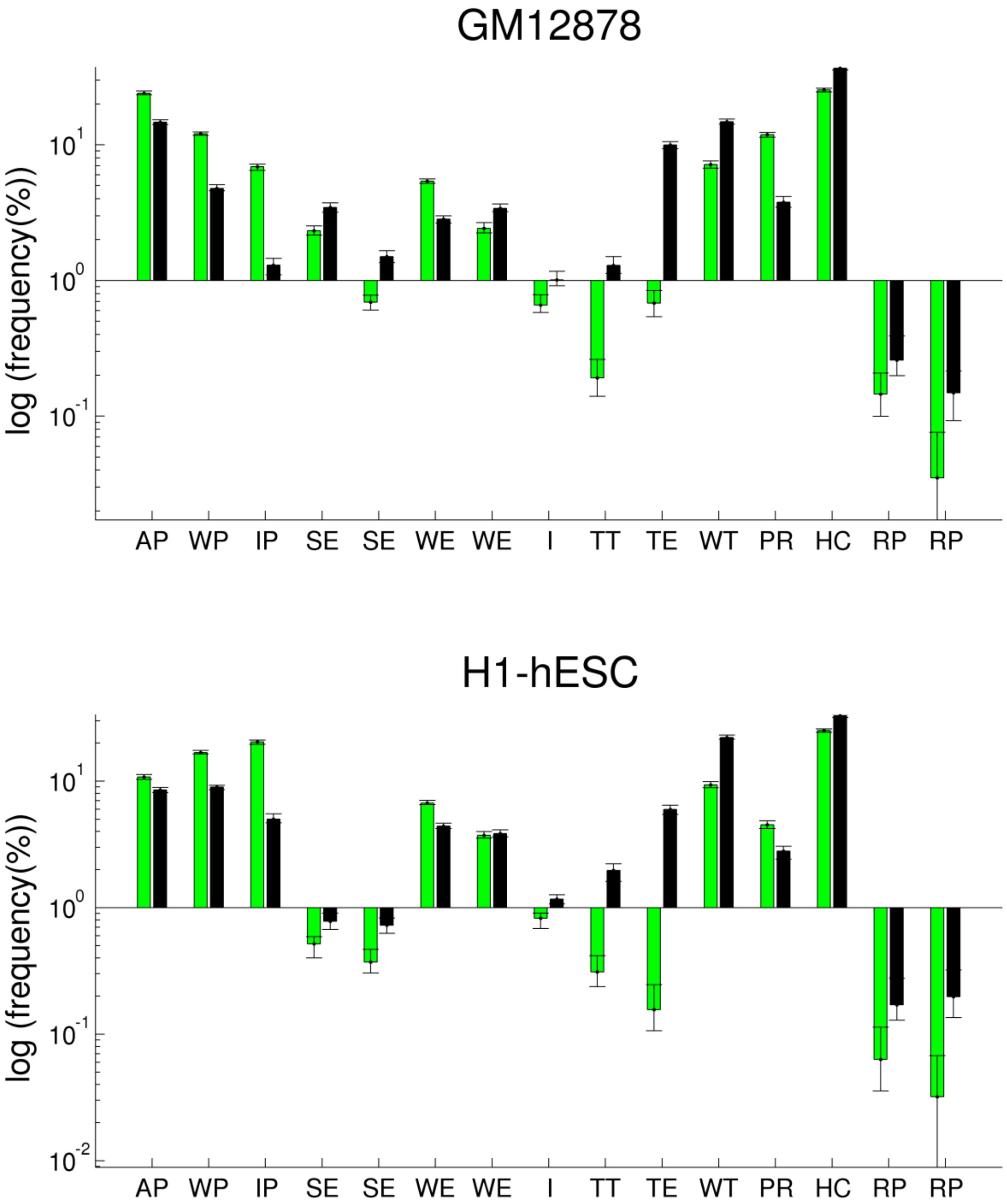
GM12878
log (frequency(%))
10^1
10^0
10^{-1}
AP WP IP SE SE WE WE I TT TE WT PR HC RP RP
H1-hESC
log (frequency(%))
10^1
10^0
10^{-1}
10^{-2}
AP WP IP SE SE WE WE I TT TE WT PR HC RP RP

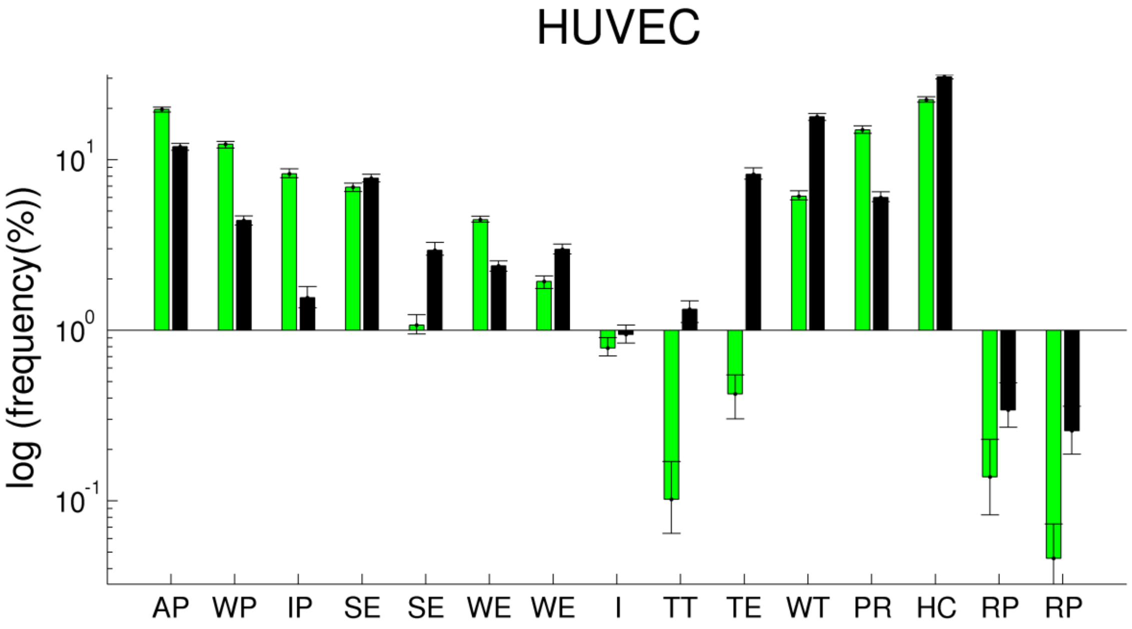

Fig. S5. Distribution of chromatin states in cell lines with normal karyotypes across promoters of protein-coding and lncRNA genes with similar expression. Green bar corresponds to promoters of coding genes from complete promoter set (CPS), black bar corresponds to promoters of lncRNAs from CPS. This figure demonstrates percentage of all promoters overlapping with chromatin states. At the end of each bar 5-95% bootstrap confidence interval of the statistic is shown. AP: Active Promoter, WP: Weak Promoter, IP: Inactive Promoter, SE: Strong Enhancer, WE: Weak Enhancer, I: Insulator, TT: Transcriptional Transition, TE: Transcriptional Elongation, WT: Weakly Transcribed, PR: Polycomb Repressed, HC: Heterochromatin low signal, RP: Repetitive/Copy number variation.
