# Supplementary material for: Promoter Analysis Reveals Globally Differential Regulation of Human Long Non-Coding RNA and Protein-Coding Genes
Source: PLoS One. 2014 Oct 2;9(10):e109443. doi: 10.1371/journal.pone.0109443 (PMC4183604; doi:10.1371/journal.pone.0109443)
Supplement: Figure S6 — Boxplot and Quartile-Quartile plot for expression value of protein-coding genes and lncRNA genes from complete promoter set (CPS) in different cell lines. (PDF) [file pone.0109443.s006.pdf]

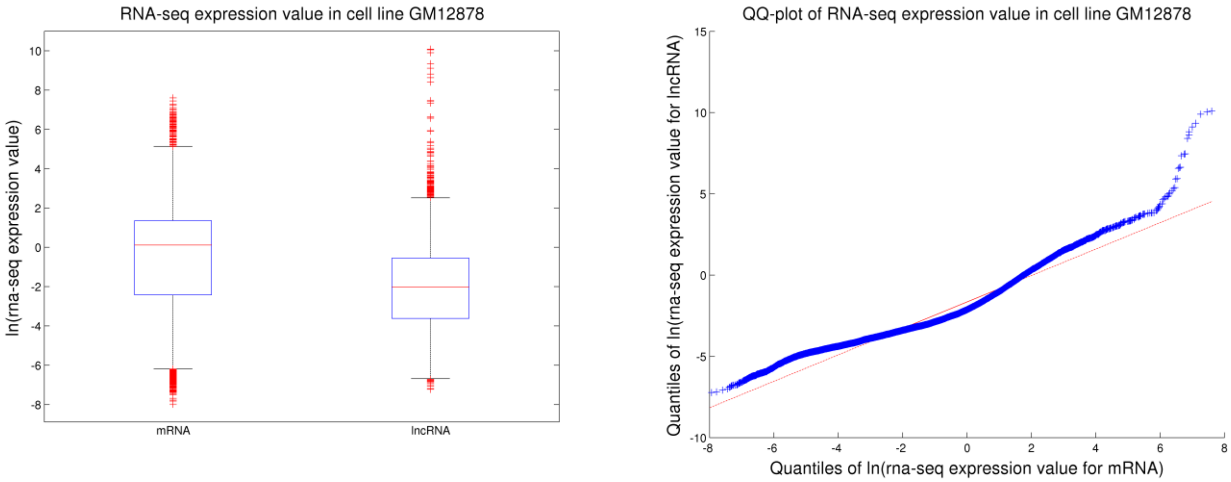

All transcripts having non-zero expression values in cell line Gm12878

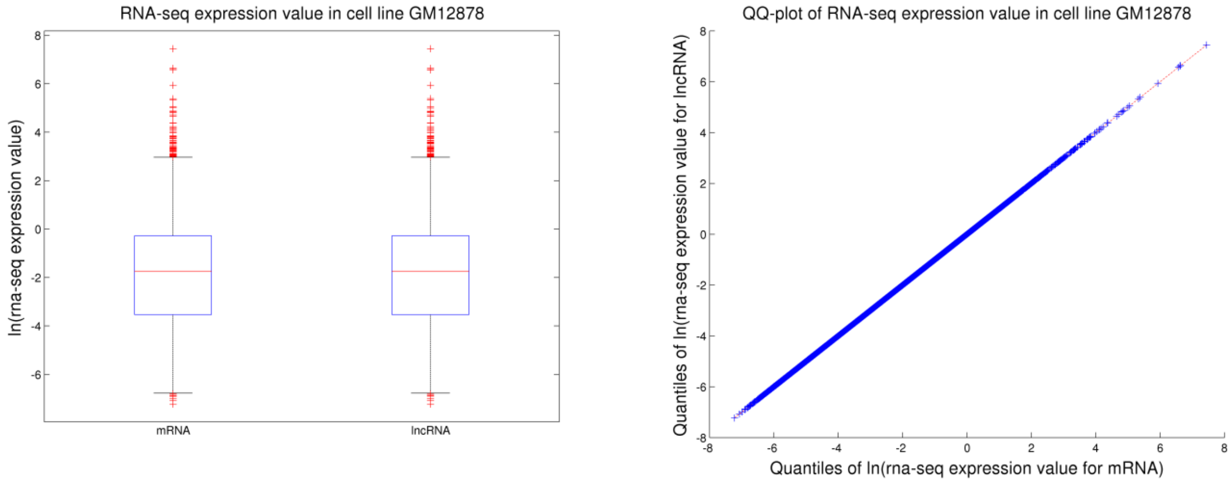

All transcripts having non-zero and similar expression values in cell line Gm12878

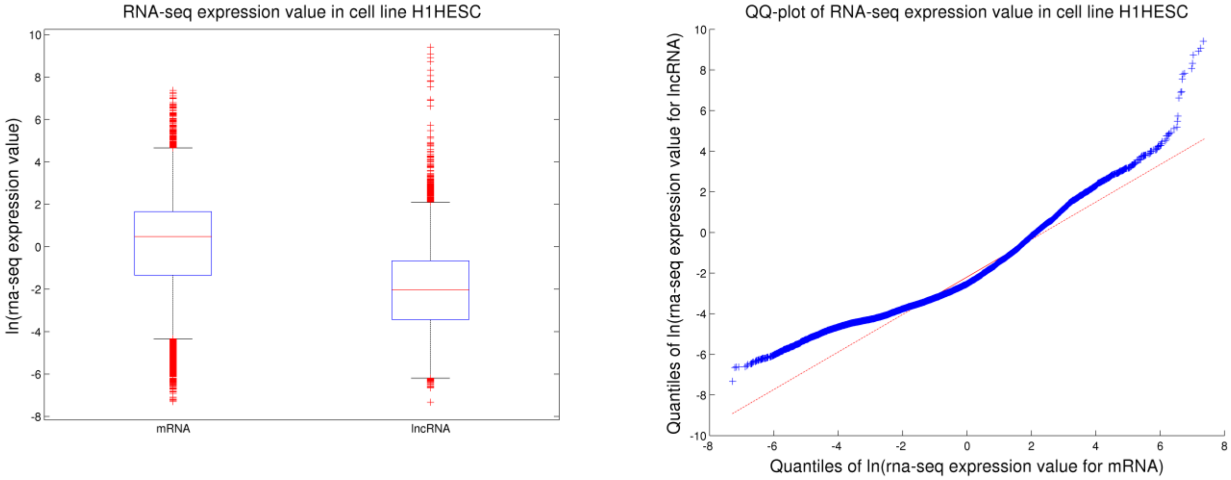

All transcripts having non-zero expression values in cell line H1-hESC

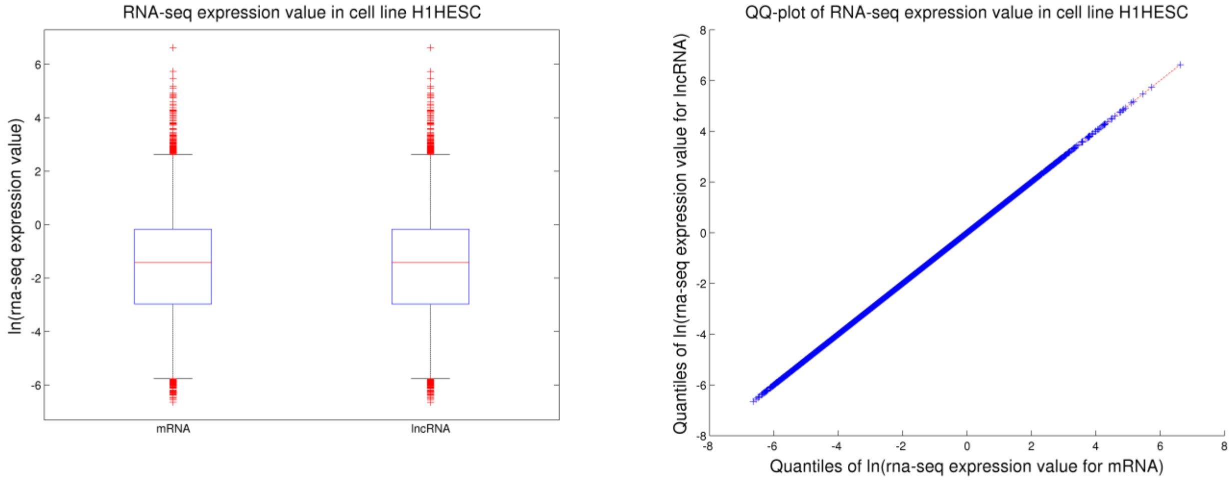

All transcripts having non-zero and similar expression values in cell line H1-hESC

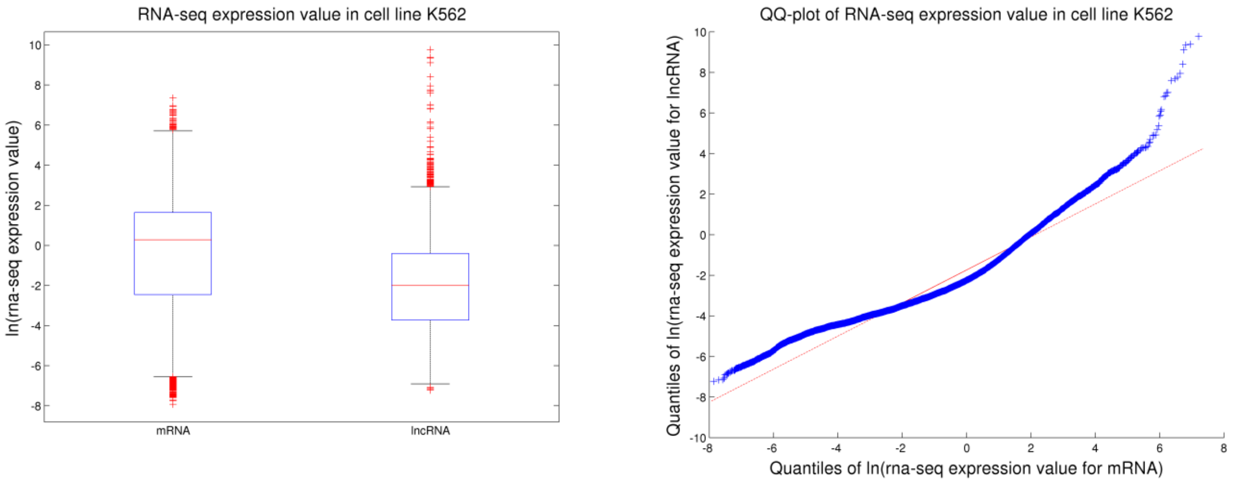

All transcripts having non-zero expression values in cell line K562

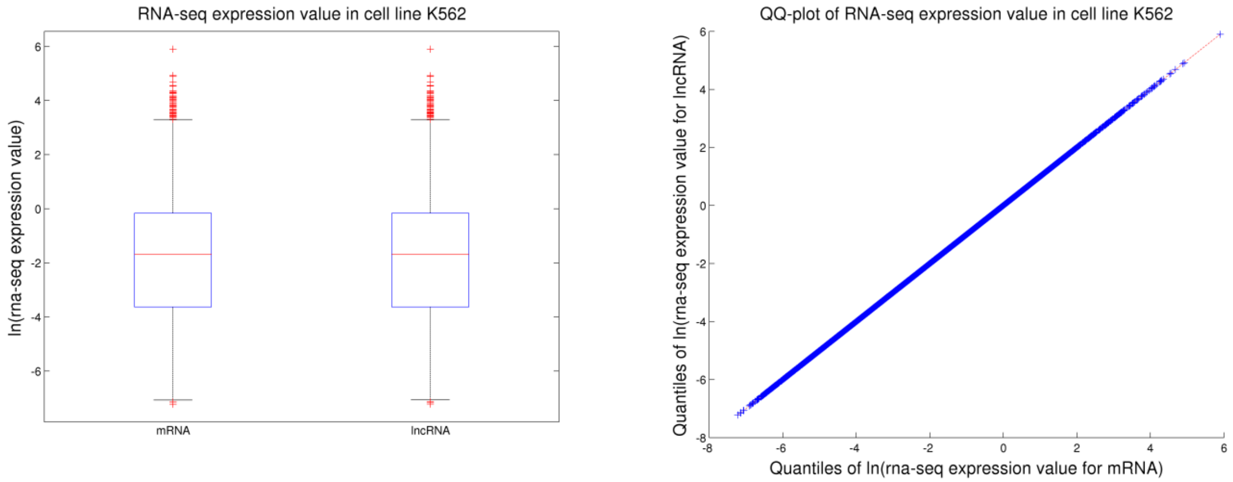

All transcripts having non-zero and similar expression values in cell line K562

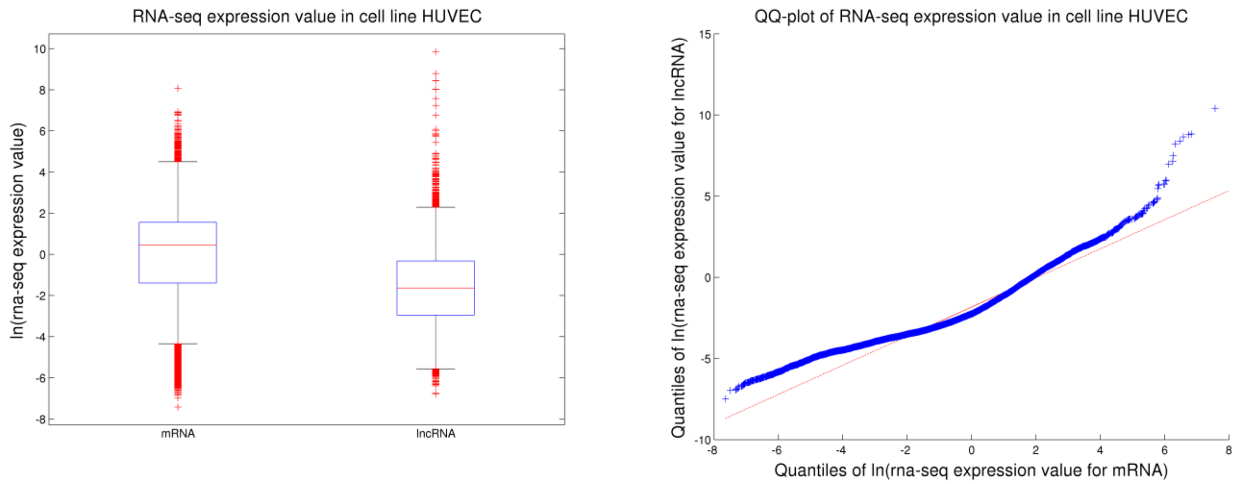

All transcripts having non-zero expression values in cell line HUVEC

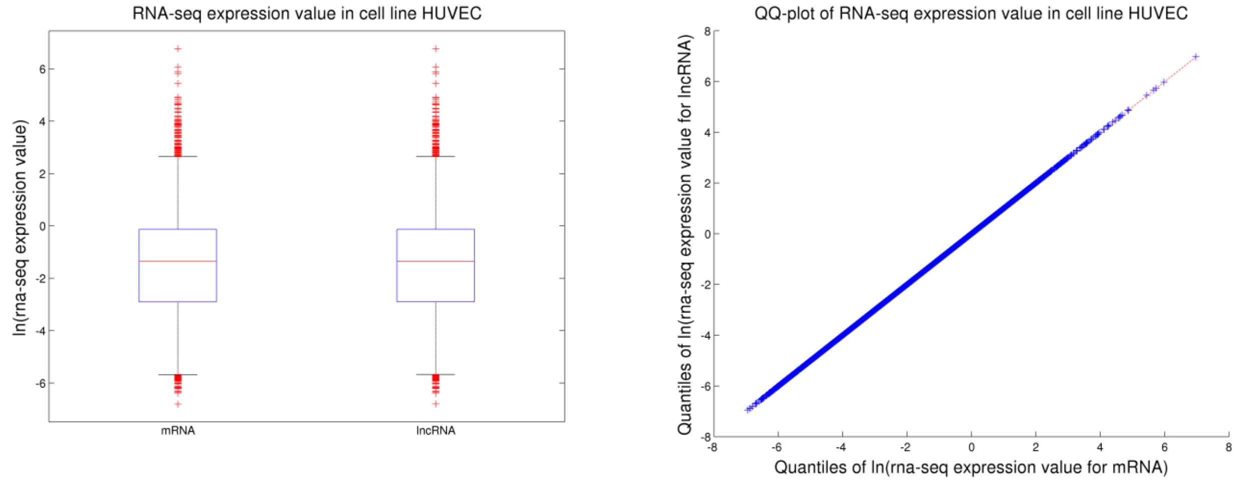

All transcripts having non-zero and similar expression values in cell line HUVEC

Figure S6: Boxplot and Quartile-Quartile plot for expression value of protein-coding genes and lncRNA genes from complete promoter set (CPS) in different cell lines.
