# Supplementary material for: Promoter Analysis Reveals Globally Differential Regulation of Human Long Non-Coding RNA and Protein-Coding Genes
Source: PLoS One. 2014 Oct 2;9(10):e109443. doi: 10.1371/journal.pone.0109443 (PMC4183604; doi:10.1371/journal.pone.0109443)
Supplement: Table S1 — Mono-, di-, and tri-nucleotides frequency and observed/expected ratio for both complete promoter set (CPS) and repeat-filtered promoter set (REFPS). (PDF) [file pone.0109443.s007.pdf]

Table S1. Mono-, di-, and tri-nucleotides frequency and observed/expected ratio for both complete promoter set (CPS) and repeat-filtered promoter set (REFPS).

Obs/Exp (di) = observed to expected ration of dinucleotides. Expected number is estimated form the assumption of independent nucleotide distribution
Obs/Exp (tri) = observed to expected ration of trinucleotides. Expected number is estimated form the assumption of independent nucleotide distribution
Upstream region = [-1000..0]
Downstream regions = [0..1000]

Complete promoter set (CPS)

Protein-coding genes									Long non-coding genes									Coding/noncoding ratio								
Nucleotides	Nucleotide probability		Di-nucleotides	Obs/Exp (di)		Tri-nucleotides	Obs/Exp (tri)		Nucleotides	Nucleotide probability		Di-nucleotides	Obs/Exp (di)		Tri-nucleotides	Obs/Exp (tri)		Nucleotides	Nucleotide probability		Di-nucleotides	Obs/Exp (di)		Tri-nucleotides	Obs/Exp (tri)	
	Up-stream region	Down-stream region		Up-stream region	Down-stream region		Up-stream region	Down-stream region		Up-stream region	Down-stream region		Up-stream region	Down-stresm region		Up-stream region	Down-stream region		Up-stream region	Down-stream region		Up-stream region	Down-stream region		Up-stream region	Down-stream region
A	0.238	0.203	AA	1.236	1.241	AAA	1.912	1.943	A	0.2674	0.2662	AA	1.158	1.146	AAA	1.629	1.570	A	0.891	0.761	AA	1.068	1.083	AAA	1.173	1.238
C	0.264	0.275	AC	0.766	0.754	AAC	0.805	0.822	C	0.2361	0.2309	AC	0.799	0.806	AAC	0.792	0.801	C	1.120	1.192	AC	0.958	0.935	AAC	1.017	1.026
G	0.263	0.298	AG	1.154	1.180	AAG	1.165	1.248	G	0.2322	0.2367	AG	1.162	1.174	AAG	1.132	1.164	G	1.131	1.260	AG	0.993	1.005	AAG	1.029	1.073
T	0.235	0.224	AT	0.849	0.839	AAT	1.114	1.104	T	0.2642	0.2663	AT	0.874	0.863	AAT	1.028	1.000	T	0.888	0.842	AT	0.971	0.972	AAT	1.083	1.104
			CA	1.059	1.038	ACA	1.018	0.988				CA	1.153	1.166	ACA	1.098	1.107				CA	0.919	0.890	ACA	0.928	0.892
			CC	1.216	1.197	ACC	0.812	0.857				CC	1.235	1.236	ACC	0.872	0.893				CC	0.985	0.969	ACC	0.932	0.959
			CG	0.587	0.647	ACG	0.368	0.385				CG	0.400	0.377	ACG	0.275	0.274				CG	1.467	1.716	ACG	1.340	1.405
			CT	1.152	1.191	ACT	0.900	0.903				CT	1.158	1.180	ACT	0.889	0.899				CT	0.995	1.009	ACT	1.012	1.005
			GA	0.960	1.024	AGA	1.246	1.379				GA	0.965	0.985	AGA	1.241	1.271				GA	0.995	1.039	AGA	1.004	1.085
			GC	1.016	1.010	AGC	1.063	1.107				GC	1.024	1.008	AGC	1.111	1.122				GC	0.992	1.002	AGC	0.957	0.987
			GG	1.223	1.153	AGG	1.392	1.311				GG	1.244	1.229	AGG	1.435	1.439				GG	0.983	0.938	AGG	0.970	0.911
			GT	0.769	0.760	AGT	0.892	0.912				GT	0.796	0.802	AGT	0.884	0.885				GT	0.966	0.947	AGT	1.009	1.031
			TA	0.733	0.697	ATA	0.787	0.747				TA	0.731	0.720	ATA	0.790	0.758				TA	1.004	0.968	ATA	0.996	0.985
			TC	0.972	0.964	ATC	0.689	0.710				TC	0.969	0.979	ATC	0.708	0.721				TC	1.004	0.985	ATC	0.974	0.984
			TG	1.056	1.062	ATG	0.816	0.817				TG	1.154	1.158	ATG	0.956	0.954				TG	0.915	0.917	ATG	0.854	0.856
			TT	1.236	1.229	ATT	1.125	1.105				TT	1.162	1.154	ATT	1.031	1.008				TT	1.064	1.066	ATT	1.092	1.096
						CAA	0.982	0.948							CAA	1.005	1.006							CAA	0.977	0.942
						CAC	0.990	0.886							CAC	1.132	1.136							CAC	0.874	0.780
						CAG	1.416	1.386							CAG	1.576	1.609							CAG	0.898	0.861
						CAT	0.811	0.836							CAT	0.943	0.953							CAT	0.860	0.877
						CCA	1.224	1.196							CCA	1.368	1.403							CCA	0.895	0.852
						CCC	1.502	1.425							CCC	1.540	1.523							CCC	0.975	0.936
						CCG	0.768	0.834							CCG	0.575	0.527							CCG	1.336	1.584
						CCT	1.377	1.396							CCT	1.402	1.445							CCT	0.982	0.966
						CGA	0.377	0.453							CGA	0.255	0.255							CGA	1.479	1.777
						CGC	0.789	0.796							CGC	0.550	0.482							CGC	1.435	1.652
						CGG	0.770	0.833							CGG	0.563	0.527							CGG	1.368	1.580
						CGT	0.364	0.388							CGT	0.268	0.273							CGT	1.357	1.424
						CTA	0.662	0.630							CTA	0.666	0.664							CTA	0.995	0.948
						CTC	1.339	1.288							CTC	1.346	1.366							CTC	0.995	0.943
						CTG	1.400	1.443							CTG	1.573	1.607							CTG	0.890	0.898
						CTT	1.157	1.237							CTT	1.121	1.152							CTT	1.032	1.074
						GAA	1.120	1.233							GAA	1.101	1.131							GAA	1.018	1.090
						GAC	0.694	0.747							GAC	0.724	0.748							GAC	0.959	0.999
						GAG	1.322	1.371							GAG	1.336	1.349							GAG	0.989	1.016
						GAT	0.689	0.707							GAT	0.712	0.716							GAT	0.968	0.988
						GCA	0.957	0.942							GCA	1.079	1.087							GCA	0.888	0.866
						GCC	1.260	1.196							GCC	1.345	1.318							GCC	0.937	0.907
						GCG	0.790	0.812							GCG	0.530	0.481							GCG	1.489	1.687
						GCT	1.047	1.104							GCT	1.111	1.124							GCT	0.943	0.982
						GGA	1.229	1.278							GGA	1.235	1.256							GGA	0.996	1.017
						GGC	1.265	1.166							GGC	1.348	1.298							GGC	0.938	0.898

Table S1. Mono-, di-, and tri-nucleotides frequency and observed/expected ratio for both complete promoter set (CPS) and repeat-filtered promoter set (REFPS).

GGG	1.518	1.319	GGG	1.561	1.506	GGG	0.972	0.876
GGT	0.834	0.798	GGT	0.876	0.889	GGT	0.951	0.898
GTA	0.551	0.539	GTA	0.572	0.585	GTA	0.963	0.921
GTC	0.705	0.685	GTC	0.727	0.732	GTC	0.970	0.936
GTG	0.992	0.957	GTG	1.124	1.130	GTG	0.882	0.847
GTT	0.808	0.785	GTT	0.792	0.785	GTT	1.021	1.000
TAA	0.961	0.970	TAA	0.863	0.851	TAA	1.113	1.140
TAC	0.551	0.535	TAC	0.571	0.574	TAC	0.963	0.932
TAG	0.654	0.604	TAG	0.666	0.647	TAG	0.983	0.934
TAT	0.796	0.768	TAT	0.794	0.777	TAT	1.002	0.988
TCA	1.022	1.013	TCA	1.077	1.085	TCA	0.949	0.933
TCC	1.250	1.223	TCC	1.228	1.252	TCC	1.018	0.977
TCG	0.377	0.431	TCG	0.255	0.255	TCG	1.478	1.690
TCT	1.269	1.310	TCT	1.250	1.278	TCT	1.015	1.025
TGA	1.023	1.060	TGA	1.079	1.086	TGA	0.948	0.977
TGC	0.944	0.971	TGC	1.073	1.087	TGC	0.880	0.893
TGG	1.226	1.175	TGG	1.375	1.374	TGG	0.892	0.855
TGT	1.024	1.022	TGT	1.104	1.097	TGT	0.928	0.932
TTA	0.959	0.945	TTA	0.865	0.849	TTA	1.108	1.113
TTC	1.140	1.167	TTC	1.104	1.120	TTC	1.032	1.042
TTG	0.979	0.955	TTG	1.001	0.995	TTG	0.979	0.960
TTT	1.911	1.922	TTT	1.653	1.624	TTT	1.156	1.183

Table S1. Mono-, di-, and tri-nucleotides frequency and observed/expected ratio for both complete promoter set (CPS) and repeat-filtered promoter set (REFPS).

Obs/Exp (di) = observed to expected ration of dinucleotides. Expected number is estimated form the assumption of independent nucleotide distribution
Obs/Exp (tri) = observed to expected ration of trinucleotides. Expected number is estimated form the assumption of independent nucleotide distribution
Upstream region = [-1000..0]
Downstream regions = [0..1000]

Repeat-filtered promoter set (REFPS)

Protein-coding genes

Nucleotides	Nucleotide probability		Di-nucleotides	Obs/Exp (di)		Tri-nucleotides	Obs/Exp (tri)	
	Up-stream region	Down-stream region		Up-stream region	Down-stream region		Up-stream region	Down-stream region
A	0.253	0.221	AA	1.197	1.197	AAA	1.750	1.749
C	0.250	0.257	AC	0.790	0.780	AAC	0.817	0.830
G	0.247	0.280	AG	1.174	1.194	AAG	1.164	1.244
T	0.250	0.242	AT	0.836	0.825	AAT	1.048	1.024
			CA	1.098	1.084	ACA	1.012	0.994
			CC	1.217	1.213	ACC	0.863	0.901
			CG	0.498	0.550	ACG	0.356	0.371
			CT	1.173	1.214	ACT	0.919	0.925
			GA	0.973	1.028	AGA	1.239	1.357
			GC	1.009	0.992	AGC	1.103	1.117
			GG	1.226	1.170	AGG	1.439	1.364
			GT	0.791	0.783	AGT	0.914	0.927
			TA	0.724	0.691	ATA	0.729	0.694
			TC	0.982	0.981	ATC	0.706	0.726
			TG	1.101	1.100	ATG	0.855	0.844
			TT	1.195	1.181	ATT	1.054	1.022
						CAA	1.003	0.971
						CAC	1.034	0.947
						CAG	1.513	1.485
						CAT	0.846	0.865
						CCA	1.309	1.299
						CCC	1.467	1.447
						CCG	0.650	0.713
						CCT	1.424	1.461
						CGA	0.346	0.404
						CGC	0.654	0.660
						CGG	0.645	0.716
						CGT	0.346	0.374
						CTA	0.687	0.656
						CTC	1.348	1.315
						CTG	1.504	1.540
						CTT	1.156	1.231
						GAA	1.121	1.225
						GAC	0.729	0.776
						GAG	1.334	1.370
						GAT	0.709	0.718
						GCA	1.011	0.981
						GCC	1.282	1.207
						GCG	0.641	0.675
						GCT	1.090	1.136
						GGA	1.244	1.294
						GGC	1.279	1.177
						GGG	1.495	1.341

Long non-coding genes

Nucleotides	Nucleotide probability		Di-nucleotides	Obs/Exp (di)		Tri-nucleotides	Obs/Exp (tri)	
	Up-stream region	Down-stream region		Up-stream region	Down-stresm region		Up-stream region	Down-stream region
A	0.271	0.269	AA	1.144	1.134	AAA	1.574	1.521
C	0.232	0.229	AC	0.810	0.810	AAC	0.805	0.808
G	0.229	0.234	AG	1.185	1.196	AAG	1.144	1.180
T	0.269	0.268	AT	0.858	0.853	AAT	1.000	0.978
			CA	1.183	1.187	ACA	1.093	1.094
			CC	1.230	1.234	ACC	0.904	0.913
			CG	0.329	0.325	ACG	0.260	0.261
			CT	1.183	1.199	ACT	0.909	0.916
			GA	0.976	0.994	AGA	1.250	1.285
			GC	1.010	0.999	AGC	1.148	1.148
			GG	1.240	1.226	AGG	1.471	1.471
			GT	0.807	0.806	AGT	0.904	0.903
			TA	0.713	0.707	ATA	0.734	0.716
			TC	0.980	0.987	ATC	0.724	0.731
			TG	1.184	1.179	ATG	0.972	0.971
			TT	1.146	1.144	ATT	0.997	0.987
						CAA	1.032	1.023
						CAC	1.148	1.133
						CAG	1.655	1.672
						CAT	0.961	0.969
						CCA	1.439	1.456
						CCC	1.499	1.500
						CCG	0.454	0.442
						CCT	1.444	1.473
						CGA	0.229	0.233
						CGC	0.410	0.387
						CGG	0.450	0.442
						CGT	0.256	0.259
						CTA	0.682	0.674
						CTC	1.353	1.367
						CTG	1.655	1.669
						CTT	1.135	1.167
						GAA	1.105	1.136
						GAC	0.746	0.762
						GAG	1.350	1.359
						GAT	0.724	0.726
						GCA	1.113	1.113
						GCC	1.327	1.309
						GCG	0.399	0.387
						GCT	1.148	1.152
						GGA	1.248	1.265
						GGC	1.330	1.290
						GGG	1.524	1.474

Coding/noncoding ratio

Nucleotides	Nucleotide probability		Di-nucleotides	Obs/Exp (di)		Tri-nucleotides	Obs/Exp (tri)	
	Up-stream region	Down-stream region		Up-stream region	Down-stream region		Up-stream region	Down-stream region
A	0.935	0.821	AA	1.046	1.056	AAA	1.112	1.150
C	1.081	1.122	AC	0.975	0.962	AAC	1.015	1.027
G	1.079	1.195	AG	0.991	0.999	AAG	1.018	1.054
T	0.928	0.904	AT	0.975	0.967	AAT	1.048	1.048
			CA	0.928	0.913	ACA	0.925	0.909
			CC	0.989	0.983	ACC	0.954	0.987
			CG	1.511	1.695	ACG	1.370	1.423
			CT	0.991	1.012	ACT	1.011	1.010
			GA	0.997	1.035	AGA	0.991	1.056
			GC	0.999	0.993	AGC	0.960	0.973
			GG	0.989	0.954	AGG	0.979	0.927
			GT	0.980	0.972	AGT	1.012	1.027
			TA	1.015	0.977	ATA	0.994	0.969
			TC	1.001	0.994	ATC	0.975	0.993
			TG	0.930	0.932	ATG	0.880	0.870
			TT	1.043	1.033	ATT	1.057	1.036
						CAA	0.972	0.949
						CAC	0.900	0.836
						CAG	0.914	0.888
						CAT	0.880	0.893
						CCA	0.909	0.892
						CCC	0.979	0.965
						CCG	1.434	1.612
						CCT	0.987	0.992
						CGA	1.512	1.732
						CGC	1.595	1.702
						CGG	1.434	1.619
						CGT	1.347	1.445
						CTA	1.007	0.974
						CTC	0.996	0.962
						CTG	0.909	0.923
						CTT	1.019	1.055
						GAA	1.014	1.079
						GAC	0.977	1.019
						GAG	0.988	1.008
						GAT	0.980	0.988
						GCA	0.908	0.882
						GCC	0.966	0.922
						GCG	1.606	1.744
						GCT	0.950	0.986
						GGA	0.996	1.023
						GGC	0.961	0.912
						GGG	0.981	0.910

Table S1. Mono-, di-, and tri-nucleotides frequency and observed/expected ratio for both complete promoter set (CPS) and repeat-filtered promoter set (REFPS).

GGT	0.882	0.846	GGT	0.906	0.909	GGT	0.974	0.931
GTA	0.577	0.561	GTA	0.576	0.587	GTA	1.001	0.955
GTC	0.734	0.718	GTC	0.747	0.748	GTC	0.983	0.961
GTG	1.033	1.004	GTG	1.141	1.124	GTG	0.905	0.893
GTT	0.822	0.796	GTT	0.804	0.794	GTT	1.023	1.003
TAA	0.900	0.897	TAA	0.836	0.833	TAA	1.077	1.077
TAC	0.576	0.556	TAC	0.576	0.576	TAC	1.001	0.965
TAG	0.681	0.628	TAG	0.678	0.655	TAG	1.005	0.959
TAT	0.733	0.715	TAT	0.736	0.736	TAT	0.996	0.973
TCA	1.056	1.054	TCA	1.109	1.111	TCA	0.952	0.949
TCC	1.256	1.252	TCC	1.240	1.260	TCC	1.013	0.994
TCG	0.344	0.395	TCG	0.231	0.232	TCG	1.488	1.699
TCT	1.254	1.300	TCT	1.259	1.287	TCT	0.996	1.010
TGA	1.063	1.080	TGA	1.110	1.110	TGA	0.957	0.973
TGC	1.001	1.011	TGC	1.112	1.114	TGC	0.900	0.908
TGG	1.321	1.270	TGG	1.442	1.427	TGG	0.916	0.890
TGT	1.019	1.010	TGT	1.096	1.083	TGT	0.930	0.932
TTA	0.897	0.875	TTA	0.833	0.830	TTA	1.077	1.055
TTC	1.134	1.161	TTC	1.111	1.126	TTC	1.021	1.031
TTG	1.008	0.973	TTG	1.023	1.013	TTG	0.985	0.961
TTT	1.741	1.717	TTT	1.593	1.585	TTT	1.093	1.083
