# Supplementary material for: Promoter Analysis Reveals Globally Differential Regulation of Human Long Non-Coding RNA and Protein-Coding Genes
Source: PLoS One. 2014 Oct 2;9(10):e109443. doi: 10.1371/journal.pone.0109443 (PMC4183604; doi:10.1371/journal.pone.0109443)
Supplement: Table S2 — Transcription factor binding sites overrepresented in promoters of protein-coding and lncRNA genes for complete promoter set (CPS) and repeat-filtered promoter set (REFPS) and support provided by DNAseI and ChIP-seq peaks. (PDF) [file pone.0109443.s008.pdf]

Table S2. Transcription factors overrepresented in promoters of protein-coding and lncRNA genes for complete promoter set (CPS) and repeat-filtered promoter set (REFPS) and support provided by DNAseI and ChIP-Seq peaks.

Complete Promoter Set (CPS)

Known TFBSs from HOCOMOCO overrepresented in promoters of mRNA genes

HOCOMOCO motif name	P-Value (fisher's Right-side Exact test)	Benjamini–Hochberg FDR correction (0.05)	FDR significance	Distance from the ideal motif family
SP4_f1	0.00000E+00	6.756757E-04	ok	0.403500
MECP2_f1	0.00000E+00	1.351351E-03	ok	0.425080
EGR1_f2	0.00000E+00	2.027027E-03	ok	0.441990
SP2_si	0.00000E+00	2.702703E-03	ok	0.452660
AP2B_f1	0.00000E+00	3.378378E-03	ok	0.473310
WT1_f1	0.00000E+00	4.054054E-03	ok	0.473310
KLF6_si	0.00000E+00	4.729730E-03	ok	0.477240
EGR2_si	0.00000E+00	5.405405E-03	ok	0.478110
MBD2_si	0.00000E+00	6.081081E-03	ok	0.482840
ZBT7B_si	0.00000E+00	6.756757E-03	ok	0.491760
NRF1_f1	0.00000E+00	7.432432E-03	ok	0.492800
KLF15_f1	0.00000E+00	8.108108E-03	ok	0.499520
ZN148_si	0.00000E+00	8.783784E-03	ok	0.503140
PLAG1_f1	0.00000E+00	9.459459E-03	ok	0.508070
MAZ_f1	0.00000E+00	1.013514E-02	ok	0.513620
E2F1_f2	0.00000E+00	1.081081E-02	ok	0.515900
ZBT7A_f1	0.00000E+00	1.148649E-02	ok	0.521200
ZFX_f1	0.00000E+00	1.216216E-02	ok	0.523120
ZN219_f1	0.00000E+00	1.283784E-02	ok	0.523730
CTCF_f2	0.00000E+00	1.351351E-02	ok	0.525420

Table S2. Transcription factors overrepresented in promoters of protein-coding and lncRNA genes for complete promoter set (CPS) and repeat-filtered promoter set (REFPS) and support provided by DNAseI and ChIP-Seq peaks.

PURA_f1	0.00000E+00	1.418919E-02	ok	0.537280
E2F6_f1	0.00000E+00	1.486486E-02	ok	0.538860
NR0B1_si	0.00000E+00	1.554054E-02	ok	0.542160
GABPA_f1	0.00000E+00	1.621622E-02	ok	0.543150
TFCP2_f1	0.00000E+00	1.689189E-02	ok	0.556040
PLAG1_si	0.00000E+00	1.756757E-02	ok	0.559620
SP3_f1	0.00000E+00	1.824324E-02	ok	0.561320
SP1_f1	0.00000E+00	1.891892E-02	ok	0.565830
ZIC1_f1	0.00000E+00	1.959459E-02	ok	0.567330
E2F4_do	0.00000E+00	2.027027E-02	ok	0.569760
GLIS3_f1	0.00000E+00	2.094595E-02	ok	0.573440
SP1_f2	0.00000E+00	2.162162E-02	ok	0.575280
HEN1_si	0.00000E+00	2.229730E-02	ok	0.577700
KLF4_f2	0.00000E+00	2.297297E-02	ok	0.579070
GABP1+GABP2_f1	0.00000E+00	2.364865E-02	ok	0.586050
AP2A_f2	0.00000E+00	2.432432E-02	ok	0.588960
HES1_f1	0.00000E+00	2.500000E-02	ok	0.591500
AP2C_f1	0.00000E+00	2.567568E-02	ok	0.598900
AP2D_f1	0.00000E+00	2.635135E-02	ok	0.689600
E2F2_f1	0.00000E+00	2.702703E-02	ok	0.693920
SRBP2_f1	3.09577E-271	2.770270E-02	ok	0.631840
PLAL1_si	2.78052E-267	2.837838E-02	ok	0.631070
FLI1_f1	5.03451E-259	2.905405E-02	ok	0.587740
RREB1_si	8.58052E-259	2.972973E-02	ok	0.679740
ARNT2_si	1.26920E-257	3.040541E-02	ok	0.588580
PAX5_f1	1.39939E-257	3.108108E-02	ok	0.583080
AHR_si	9.95798E-249	3.175676E-02	ok	0.699810
MZF1_f1	1.10901E-247	3.243243E-02	ok	0.618940
ETS2_f1	1.92601E-236	3.310811E-02	ok	0.637820
COE1_f2	2.38333E-225	3.378378E-02	ok	0.603350
ZIC2_f1	1.67662E-211	3.445946E-02	ok	0.605100

Table S2. Transcription factors overrepresented in promoters of protein-coding and lncRNA genes for complete promoter set (CPS) and repeat-filtered promoter set (REFPS) and support provided by DNAseI and ChIP-Seq peaks.

ELK1_f1	9.51385E-203	3.513514E-02	ok	0.614590
REST_f1	4.69131E-202	3.581081E-02	ok	0.607810
EGR3_f1	6.60587E-202	3.648649E-02	ok	0.695320
KLF1_f1	3.11315E-174	3.716216E-02	ok	0.647570
MYCN_si	3.85610E-136	3.783784E-02	ok	0.683430
GT2D1_f1	1.28782E-121	3.851351E-02	ok	0.623620
NFKB1_f1	2.79249E-119	3.918919E-02	ok	0.629000
ELF1_f1	1.74869E-114	3.986486E-02	ok	0.624980
MYC_f1	1.02765E-87	4.054054E-02	ok	0.673860
ZIC3_f1	1.87911E-84	4.121622E-02	ok	0.638120
KAISO_f1	3.58263E-83	4.189189E-02	ok	0.665770
ZEP2_si	1.27533E-81	4.256757E-02	ok	0.689350
SMAD2_si	4.15610E-65	4.324324E-02	ok	0.670980
MAFA_f1	1.11993E-52	4.391892E-02	ok	0.656770
MYOG_f1	1.35744E-47	4.459459E-02	ok	0.678930
YBOX1_f2	2.23871E-40	4.527027E-02	ok	0.693470
ESR1_do	1.43418E-36	4.594595E-02	ok	0.667000
NDF1_f1	1.24066E-34	4.662162E-02	ok	0.679420
SRBP1_f2	1.75397E-29	4.729730E-02	ok	0.668320
PAX5_si	2.14817E-29	4.797297E-02	ok	0.678860
TF65_f2	1.10303E-14	4.864865E-02	ok	0.691000
NFIA+NFIB+NFIC+NFIX_f2	2.00108E-11	4.932432E-02	ok	0.695140
ETS1_si	8.02230E-09	5.000000E-02	ok	0.696740

Table S2. Transcription factors overrepresented in promoters of protein-coding and lncRNA genes for complete promoter set (CPS) and repeat-filtered promoter set (REFPS) and support provided by DNAseI and ChIP-Seq peaks.

Complete Promoter Set (CPS)									
Known TFBSs from HOCOMOCO overrepresented in promoters of lncRNA genes					Ab initio motif families overrepresented in promoters of lncRNA genes and similar TFBSs from HOCOMOCO				
**HOCOMOCO motif name**	**P-Value (fisher's Right-side Exact test)**	**Benjamini–Hochberg FDR correction (0.05)**	**FDR significance**	**Distance from the ideal motif family**	**HOCOMOCO motif name**	**Ab initio motif family ID**	**Q-Value of similarity**	**P-Value of over-representation for ab initio motif using Fisher's right side exact test**	**Distance of ab initio motif from ideal motif family**
HMGA1_f1	0	0.000357143	ok	0.50975	NKX25_f1	204	0.0484951	2.3991E-172	0.62873708
SRY_f1	0	0.000714286	ok	0.50977	NKX32_f1	204	0.0484951	2.3991E-172	0.62873708
FOXO1_si	0	0.001071429	ok	0.51799	PRDM1_f1	204	0.0484951	2.3991E-172	0.62873708
FUBP1_f1	0	0.001428571	ok	0.5282					
FOXA1_f1	0	0.001785714	ok	0.53548					
FOXA2_f1	0	0.002142857	ok	0.53708					
IRF4_si	0	0.0025	ok	0.54987					
IRF7_f1	0	0.002857143	ok	0.55136	**New TFBS name**	**p-value of over-representation**	**Distance form ideal point**		
BPTF_si	0	0.003214286	ok	0.55203	NKX32_f1	1.78E-26	0.741		
FOXM1_f1	0	0.003571429	ok	0.55254					
FOXO3_si	0	0.003928571	ok	0.55707					
CDX2_f1	0	0.004285714	ok	0.55819					
SOX2_f1	0	0.004642857	ok	0.56036					
PO5F1_do	0	0.005	ok	0.56087					
STAT2_f1	0	0.005357143	ok	0.56459					
FOXP2_si	0	0.005714286	ok	0.5647					
SOX5_f1	0	0.006071429	ok	0.567					
NR2E3_f1	0	0.006428571	ok	0.58001					
PO2F1_f1	0	0.006785714	ok	0.58026					
PO3F2_si	0	0.007142857	ok	0.59147					
TBP_f1	0	0.0075	ok	0.5939					
SOX10_si	0	0.007857143	ok	0.6002					
FOXD3_f1	0	0.008214286	ok	0.60206					
EVI1_f1	0	0.008571429	ok	0.61631					
HXD10_f1	0	0.008928571	ok	0.61762					
MSX2_f1	0	0.009285714	ok	0.61975					
HXD13_f1	0	0.009642857	ok	0.61997					
CDX1_f1	0	0.01	ok	0.62756					
CEBPA_do	0	0.010357143	ok	0.62936					
FOXJ2_f1	0	0.010714286	ok	0.62988					
HXD4_f1	0	0.011071429	ok	0.6326					
FOXJ3_si	0	0.011428571	ok	0.63469					
SOX13_f1	0	0.011785714	ok	0.64062					
NKX31_si	0	0.012142857	ok	0.64207					
HNF1A_f1	0	0.0125	ok	0.64247					
FOXD1_si	0	0.012857143	ok	0.64831					
FOXF2_f1	0	0.013214286	ok	0.65482					
PIT1_f1	0	0.013571429	ok	0.65939					
GATA6_f2	0	0.013928571	ok	0.65978					

PBX1_do	0	0.014285714	ok	0.66714					
FOXP3_f1	0	0.014642857	ok	0.66722					
ARI3A_f1	0	0.015	ok	0.67776					
FOXA3_f1	0	0.015357143	ok	0.67912					
MEF2A_f1	0	0.015714286	ok	0.68147					
ARI5B_f1	0	0.016071429	ok	0.682					
ALX1_si	0	0.016428571	ok	0.68901					
HNF1B_f1	0	0.016785714	ok	0.69352					
IRF1_si	0	0.017142857	ok	0.57981					
NFAC1_si	0	0.0175	ok	0.60263					
FOXO4_f1	0	0.017857143	ok	0.66677					
SOX9_f1	1.2209E-301	0.018214286	ok	0.66331					
GATA4_f1	8.1568E-296	0.018571429	ok	0.6665					
CRX_si	1.7964E-275	0.018928571	ok	0.68986					
CEBPD_f1	1.8311E-270	0.019285714	ok	0.66851					
GATA3_si	2.6303E-259	0.019642857	ok	0.68183					
PITX2_si	5.7646E-253	0.02	ok	0.67876					
BATF_si	1.764E-248	0.020357143	ok	0.66556					
ZN384_f1	8.393E-248	0.020714286	ok	0.61785					
AIRE_f2	1.3864E-243	0.021071429	ok	0.6807					
BCL6_f1	2.3549E-242	0.021428571	ok	0.62602					
GFI1B_f1	2.1719E-237	0.021785714	ok	0.65162					
GATA2_si	5.3566E-237	0.022142857	ok	0.68691					
SOX15_f1	2.5188E-236	0.0225	ok	0.65138					
IRF3_f1	3.0784E-236	0.022857143	ok	0.59608					
UBIP1_f1	1.8885E-235	0.023214286	ok	0.61584					
GCR_si	2.3252E-222	0.023571429	ok	0.59422					
TEAD1_f1	4.5786E-220	0.023928571	ok	0.66917					
TF7L2_f1	6.0871E-215	0.024285714	ok	0.60562					
JUNB_f1	1.6331E-211	0.024642857	ok	0.66699					
STAT6_do	1.7989E-210	0.025	ok	0.64049					
NR1I2_si	2.5216E-210	0.025357143	ok	0.65305					
GFI1_f1	2.6277E-210	0.025714286	ok	0.64115					
OTX1_f1	2.2062E-207	0.026071429	ok	0.69451					
NR1I3_si	2.6362E-207	0.026428571	ok	0.64404					
MAFG_si	9.5153E-207	0.026785714	ok	0.67241					
PRGR_f1	2.201E-206	0.027142857	ok	0.60172					
LEF1_f1	2.1429E-204	0.0275	ok	0.62929					
IRF2_f1	1.7319E-195	0.027857143	ok	0.61459					
CEBPB_f1	2.1577E-191	0.028214286	ok	0.69019					
HSF1_f2	5.538E-189	0.028571429	ok	0.61318					
NKX25_f1	6.9639E-187	0.028928571	ok	0.64841					
PRDM1_f1	1.2681E-185	0.029285714	ok	0.60775					
JUND_f1	1.3612E-185	0.029642857	ok	0.65209					
PPARG_si	4.0066E-183	0.03	ok	0.61752					
NFAC2_f1	6.685E-182	0.030357143	ok	0.60325					
JUN_f1	1.38E-174	0.030714286	ok	0.66579					
NFAC4_f1	4.7843E-174	0.031071429	ok	0.6052					
STAT4_si	7.2568E-164	0.031428571	ok	0.68541					
NF2L1_f1	4.6986E-163	0.031785714	ok	0.69012					
HSF2_f2	4.6139E-160	0.032142857	ok	0.66969					
FOSL2_f1	1.1793E-157	0.0325	ok	0.65321					
NFAC3_f1	8.1777E-156	0.032857143	ok	0.62416					
FOSL1_f2	1.0732E-150	0.033214286	ok	0.67764					
COT2_f1	1.6733E-149	0.033571429	ok	0.65262					
RXRB_f1	1.608E-147	0.033928571	ok	0.63496					
RUNX1_f1	7.1988E-147	0.034285714	ok	0.64736					
RARG_f1	1.1605E-143	0.034642857	ok	0.64453					

Table S2. Transcription factors overrepresented in promoters of protein-coding and lncRNA genes for complete promoter set (CPS) and repeat-filtered promoter set (REFPS) and support provided by DNAseI and ChIP-Seq peaks.

ELF5_f1	5.07E-142	0.035	ok	0.62586					
STA5A_do	6.0833E-140	0.035357143	ok	0.65616					
ERR1_f1	5.2594E-136	0.035714286	ok	0.62969					
ERR2_f1	7.0641E-135	0.036071429	ok	0.63551					
IRF8_si	1.8366E-129	0.036428571	ok	0.6242					
SPIB_f1	1.3588E-125	0.036785714	ok	0.65455					
NF2L2_si	2.5054E-125	0.037142857	ok	0.63405					
PPARA_f1	5.6164E-125	0.0375	ok	0.65679					
PARP1_si	9.0369E-117	0.037857143	ok	0.62772					
MITF_f1	8.0556E-115	0.038214286	ok	0.65458					
VDR_f1	1.273E-113	0.038571429	ok	0.62921					
RUNX2_f1	6.6467E-113	0.038928571	ok	0.64212					
TGIF1_si	4.1793E-107	0.039285714	ok	0.66943					
TCF7_f1	4.59718E-94	0.039642857	ok	0.69088					
SPI1_si	3.76455E-88	0.04	ok	0.66576					
TBX5_si	4.88005E-88	0.040357143	ok	0.68059					
COT1_si	3.81385E-87	0.040714286	ok	0.67747					
TFE2_f2	1.68446E-82	0.041071429	ok	0.66787					
GCR_do	1.42113E-73	0.041428571	ok	0.64243					
NFE2_f2	1.81566E-72	0.041785714	ok	0.65817					
ATF5_si	1.11047E-68	0.042142857	ok	0.64445					
PEBB_f1	1.48289E-63	0.0425	ok	0.6876					
THA_f1	5.1439E-63	0.042857143	ok	0.67464					
THB_f1	8.58779E-59	0.043214286	ok	0.6797					
STF1_f1	1.45955E-47	0.043571429	ok	0.67182					
ZEB1_do	7.84789E-45	0.043928571	ok	0.65745					
ESR2_si	1.34751E-41	0.044285714	ok	0.67461					
COT1_f1	2.08944E-41	0.044642857	ok	0.67997					
ETV5_f1	5.06526E-41	0.045	ok	0.68449					
COT2_f2	7.51855E-40	0.045357143	ok	0.66559					
STAT1_f2	5.97535E-35	0.045714286	ok	0.67368					
MAF_f1	1.05446E-33	0.046071429	ok	0.67274					
HAND1_si	3.67089E-30	0.046428571	ok	0.68535					
NKX21_f1	1.6211E-27	0.046785714	ok	0.68951					
THA_f2	2.27188E-25	0.047142857	ok	0.67431					
NR5A2_f1	3.82676E-24	0.0475	ok	0.68776					
STAT3_si	9.28803E-23	0.047857143	ok	0.68123					
HNF4A_f1	5.63263E-22	0.048214286	ok	0.67792					
PPARG_f1	8.71338E-21	0.048571429	ok	0.67979					
NR2C2_f1	1.45038E-15	0.048928571	ok	0.68107					
SMAD4_si	1.36946E-14	0.049285714	ok	0.69381					
SUH_f1	2.55423E-14	0.049642857	ok	0.68875					
PPARA_f2	1.02195E-08	0.05	ok	0.69511					

Table S2. Transcription factors overrepresented in promoters of protein-coding and lncRNA genes for complete promoter set (CPS) and repeat-filtered promoter set (REFPS) and support provided by DNAseI and ChIP-Seq peaks.

Complete Promoter Set (CPS)

Known TFBSs from HOCOMOCO overrepresented in promoters of mRNA genes and their support by DNAaseI

HOCOMOCO motif name	Right-side Fisher's exact test	Benjamini–Hochberg FDR correction (0.05)	FDR significance
AP2A_f2	0.00000E+00	6.76E-004	ok
AP2B_f1	0.00000E+00	1.35E-003	ok
AP2C_f1	0.00000E+00	2.03E-003	ok
AP2D_f1	0.00000E+00	2.70E-003	ok
ARNT2_si	0.00000E+00	3.38E-003	ok
COE1_f2	0.00000E+00	4.05E-003	ok
CTCF_f2	0.00000E+00	4.73E-003	ok
E2F1_f2	0.00000E+00	5.41E-003	ok
E2F2_f1	0.00000E+00	6.08E-003	ok
E2F4_do	0.00000E+00	6.76E-003	ok
E2F6_f1	0.00000E+00	7.43E-003	ok
EGR1_f2	0.00000E+00	8.11E-003	ok
EGR2_si	0.00000E+00	8.78E-003	ok
ELF1_f1	0.00000E+00	9.46E-003	ok
ELK1_f1	0.00000E+00	1.01E-002	ok
ETS2_f1	0.00000E+00	1.08E-002	ok
FLI1_f1	0.00000E+00	1.15E-002	ok
GABP1+GABP2_f1	0.00000E+00	1.22E-002	ok
GABPA_f1	0.00000E+00	1.28E-002	ok
GLIS3_f1	0.00000E+00	1.35E-002	ok
GT2D1_f1	0.00000E+00	1.42E-002	ok
HEN1_si	0.00000E+00	1.49E-002	ok
HES1_f1	0.00000E+00	1.55E-002	ok
KLF15_f1	0.00000E+00	1.62E-002	ok
KLF4_f2	0.00000E+00	1.69E-002	ok
KLF6_si	0.00000E+00	1.76E-002	ok
MAZ_f1	0.00000E+00	1.82E-002	ok
MBD2_si	0.00000E+00	1.89E-002	ok
MECP2_f1	0.00000E+00	1.96E-002	ok
MZF1_f1	0.00000E+00	2.03E-002	ok
NR0B1_si	0.00000E+00	2.09E-002	ok
NRF1_f1	0.00000E+00	2.16E-002	ok
PAX5_f1	0.00000E+00	2.23E-002	ok
PLAG1_f1	0.00000E+00	2.30E-002	ok
PLAG1_si	0.00000E+00	2.36E-002	ok
PLAL1_si	0.00000E+00	2.43E-002	ok
PURA_f1	0.00000E+00	2.50E-002	ok
REST_f1	0.00000E+00	2.57E-002	ok
RREB1_si	0.00000E+00	2.64E-002	ok
SP1_f1	0.00000E+00	2.70E-002	ok
SP1_f2	0.00000E+00	2.77E-002	ok
SP2_si	0.00000E+00	2.84E-002	ok
SP3_f1	0.00000E+00	2.91E-002	ok
SP4_f1	0.00000E+00	2.97E-002	ok

Table S2. Transcription factors overrepresented in promoters of protein-coding and lncRNA genes for complete promoter set (CPS) and repeat-filtered promoter set (REFPS) and support provided by DNAseI and ChIP-Seq peaks.

SRBP2_f1	0.00000E+00	3.04E-002	ok
TFCP2_f1	0.00000E+00	3.11E-002	ok
WT1_f1	0.00000E+00	3.18E-002	ok
ZBT7A_f1	0.00000E+00	3.24E-002	ok
ZBT7B_si	0.00000E+00	3.31E-002	ok
ZFX_f1	0.00000E+00	3.38E-002	ok
ZIC1_f1	0.00000E+00	3.45E-002	ok
ZIC2_f1	0.00000E+00	3.51E-002	ok
ZN148_si	0.00000E+00	3.58E-002	ok
ZN219_f1	0.00000E+00	3.65E-002	ok
AHR_si	0.00000E+00	3.72E-002	ok
EGR3_f1	2.08630E-292	3.78E-002	ok
ZIC3_f1	4.63870E-291	3.85E-002	ok
NFKB1_f1	1.23300E-283	3.92E-002	ok
KLF1_f1	5.69410E-276	3.99E-002	ok
MYCN_si	1.16820E-251	4.05E-002	ok
PAX5_si	1.41950E-240	4.12E-002	ok
KAISO_f1	7.69540E-236	4.19E-002	ok
MAFA_f1	6.02380E-234	4.26E-002	ok
MYC_f1	8.92680E-230	4.32E-002	ok
SMAD2_si	8.96690E-226	4.39E-002	ok
SRBP1_f2	6.37730E-219	4.46E-002	ok
ESR1_do	4.89690E-216	4.53E-002	ok
ZEP2_si	2.30540E-214	4.59E-002	ok
ETS1_si	3.11160E-190	4.66E-002	ok
YBOX1_f2	3.73460E-186	4.73E-002	ok
MYOG_f1	9.14580E-182	4.80E-002	ok
NDF1_f1	1.89530E-171	4.86E-002	ok
NFIA+NFIB+NFIC+NFIX_f2	5.87600E-150	4.93E-002	ok
TF65_f2	6.92470E-128	5.00E-002	ok

Table S2. Transcription factors overrepresented in promoters of protein-coding and lncRNA genes for complete promoter set (CPS) and repeat filtered promoter set (REFPS) and support provided by DNAseI and ChIP-Seq peaks.

Complete Promoter Set (CPS)

Known TFBSs from HOCOMOCO overrepresented in promoters of lncRNA genes and their support by DNAaseI

HOCOMOCO motif name	Right-side Fisher's exact test	Benjamini–Hochberg FDR correction (0.05)	FDR significance
HMGA1_f1	1.51E-78	3.60E-004	ok
NKX31_si	1.78E-76	7.19E-004	ok
MSX2_f1	1.24E-59	1.08E-003	ok
SOX5_f1	7.51E-59	1.44E-003	ok
PO3F2_si	5.29E-56	1.80E-003	ok
FOXJ3_si	4.41E-55	2.16E-003	ok
FOXO1_si	6.68E-55	2.52E-003	ok
ALX1_si	8.92E-54	2.88E-003	ok
PO5F1_do	7.16E-52	3.24E-003	ok
SOX2_f1	5.89E-50	3.60E-003	ok
FOXJ2_f1	7.39E-50	3.96E-003	ok
CDX2_f1	1.41E-48	4.32E-003	ok
PIT1_f1	1.50E-48	4.68E-003	ok
SRY_f1	9.55E-47	5.04E-003	ok
FOXM1_f1	3.55E-46	5.40E-003	ok
FOXP3_f1	1.84E-45	5.76E-003	ok
HXD10_f1	1.25E-44	6.12E-003	ok
HNF1A_f1	1.60E-44	6.47E-003	ok
HXD4_f1	9.01E-44	6.83E-003	ok
MEF2A_f1	5.61E-42	7.19E-003	ok
HXD13_f1	1.19E-40	7.55E-003	ok

Table S2. Transcription factors overrepresented in promoters of protein-coding and lncRNA genes for complete promoter set (CPS) and repeat filtered promoter set (REFPS) and support provided by DNAseI and ChIP-Seq peaks.

EVI1_f1	2.21E-37	7.91E-003	ok
PO2F1_f1	1.23E-35	8.27E-003	ok
ARI3A_f1	8.66E-35	8.63E-003	ok
FUBP1_f1	7.78E-34	8.99E-003	ok
HNF1B_f1	7.55E-33	9.35E-003	ok
TBP_f1	8.21E-32	9.71E-003	ok
FOXA2_f1	1.52E-30	1.01E-002	ok
NR2E3_f1	1.97E-30	1.04E-002	ok
FOXD3_f1	1.84E-28	1.08E-002	ok
FOXF2_f1	7.74E-28	1.12E-002	ok
ARI5B_f1	3.43E-27	1.15E-002	ok
PBX1_do	3.65E-27	1.19E-002	ok
FOXO3_si	9.96E-26	1.22E-002	ok
FOXA3_f1	9.31E-25	1.26E-002	ok
GATA6_f2	7.04E-22	1.29E-002	ok
CDX1_f1	1.52E-21	1.33E-002	ok
FOXD1_si	1.80E-21	1.37E-002	ok
SOX13_f1	2.63E-20	1.40E-002	ok
BPTF_si	8.18E-17	1.44E-002	ok
SOX9_f1	8.71E-16	1.47E-002	ok
CEBPA_do	1.34E-15	1.51E-002	ok
FOXP2_si	6.37E-15	1.55E-002	ok
FOXA1_f1	1.34E-14	1.58E-002	ok
FOXO4_f1	1.57E-14	1.62E-002	ok
BATF_si	1.71E-12	1.65E-002	ok
CRX_si	2.96E-12	1.69E-002	ok
GATA4_f1	4.97E-11	1.73E-002	ok
IRF4_si	1.10E-10	1.76E-002	ok
CEBPD_f1	7.71E-10	1.80E-002	ok
PITX2_si	1.24E-09	1.83E-002	ok
AIRE_f2	6.81E-09	1.87E-002	ok
GATA2_si	3.40E-08	1.91E-002	ok
NFAC1_si	4.07E-08	1.94E-002	ok
TEAD1_f1	4.19E-08	1.98E-002	ok
GATA3_si	9.38E-08	2.01E-002	ok

Table S2. Transcription factors overrepresented in promoters of protein-coding and lncRNA genes for complete promoter set (CPS) and repeat filtered promoter set (REFPS) and support provided by DNAseI and ChIP-Seq peaks.

SOX10_si	9.15E-06	2.05E-002	ok
SOX15_f1	6.15E-05	2.09E-002	ok
GFI1B_f1	8.35E-05	2.12E-002	ok
JUNB_f1	2.03E-04	2.16E-002	ok
IRF7_f1	3.52E-04	2.19E-002	ok
NR1I2_si	4.81E-03	2.23E-002	ok
BCL6_f1	7.00E-03	2.27E-002	ok
IRF1_si	7.34E-03	2.30E-002	ok
CEBPB_f1	1.40E-02	2.34E-002	ok
ZN384_f1	1.44E-02	2.37E-002	ok
OTX1_f1	1.91E-02	2.41E-002	ok
JUN_f1	4.32E-02	2.45E-002	non-sign
STAT6_do	5.86E-02	2.48E-002	non-sign
MAFG_si	6.73E-02	2.52E-002	non-sign
NR1I3_si	1.19E-01	2.55E-002	non-sign
STAT4_si	2.04E-01	2.59E-002	non-sign
LEF1_f1	2.36E-01	2.63E-002	non-sign
FOSL1_f2	3.66E-01	2.66E-002	non-sign
GFI1_f1	3.74E-01	2.70E-002	non-sign
JUND_f1	3.74E-01	2.73E-002	non-sign
HSF2_f2	5.49E-01	2.77E-002	non-sign
NKX25_f1	6.27E-01	2.81E-002	non-sign
STAT2_f1	6.67E-01	2.84E-002	non-sign
NF2L1_f1	7.73E-01	2.88E-002	non-sign
FOSL2_f1	8.33E-01	2.91E-002	non-sign
RUNX1_f1	8.71E-01	2.95E-002	non-sign
TF7L2_f1	8.86E-01	2.99E-002	non-sign
HSF1_f2	9.43E-01	3.02E-002	non-sign
PPARG_si	9.65E-01	3.06E-002	non-sign
COT2_f1	9.70E-01	3.09E-002	non-sign
GCR_si	9.75E-01	3.13E-002	non-sign
IRF3_f1	9.78E-01	3.17E-002	non-sign
PRGR_f1	9.84E-01	3.20E-002	non-sign
IRF2_f1	9.91E-01	3.24E-002	non-sign
PPARA_f1	9.96E-01	3.27E-002	non-sign

Table S2. Transcription factors overrepresented in promoters of protein-coding and lncRNA genes for complete promoter set (CPS) and repeat filtered promoter set (REFPS) and support provided by DNAseI and ChIP-Seq peaks.

ERR2_f1	9.98E-01	3.31E-002	non-sign
RXRB_f1	9.99E-01	3.35E-002	non-sign
NFAC3_f1	9.99E-01	3.38E-002	non-sign
ERR1_f1	1.00E+00	3.42E-002	non-sign
RARG_f1	1.00E+00	3.45E-002	non-sign
STA5A_do	1.00E+00	3.49E-002	non-sign
SPIB_f1	1.00E+00	3.53E-002	non-sign
NFAC2_f1	1.00E+00	3.56E-002	non-sign
NFAC4_f1	1.00E+00	3.60E-002	non-sign
PRDM1_f1	1.00E+00	3.63E-002	non-sign
UBIP1_f1	1.00E+00	3.67E-002	non-sign
IRF8_si	1.00E+00	3.71E-002	non-sign
ELF5_f1	1.00E+00	3.74E-002	non-sign
PARP1_si	1.00E+00	3.78E-002	non-sign
VDR_f1	1.00E+00	3.81E-002	non-sign
NF2L2_si	1.00E+00	3.85E-002	non-sign
RUNX2_f1	1.00E+00	3.88E-002	non-sign
GCR_do	1.00E+00	3.92E-002	non-sign
ATF5_si	1.00E+00	3.96E-002	non-sign
MITF_f1	1.00E+00	3.99E-002	non-sign
ZEB1_do	1.00E+00	4.03E-002	non-sign
NFE2_f2	1.00E+00	4.06E-002	non-sign
COT2_f2	1.00E+00	4.10E-002	non-sign
SPI1_si	1.00E+00	4.14E-002	non-sign
TFE2_f2	1.00E+00	4.17E-002	non-sign
TGIF1_si	1.00E+00	4.21E-002	non-sign
STF1_f1	1.00E+00	4.24E-002	non-sign
MAF_f1	1.00E+00	4.28E-002	non-sign
STAT1_f2	1.00E+00	4.32E-002	non-sign
THA_f2	1.00E+00	4.35E-002	non-sign
ESR2_si	1.00E+00	4.39E-002	non-sign
THA_f1	1.00E+00	4.42E-002	non-sign
COT1_si	1.00E+00	4.46E-002	non-sign
HNF4A_f1	1.00E+00	4.50E-002	non-sign
THB_f1	1.00E+00	4.53E-002	non-sign

Table S2. Transcription factors overrepresented in promoters of protein-coding and lncRNA genes for complete promoter set (CPS) and repeat filtered promoter set (REFPS) and support provided by DNAseI and ChIP-Seq peaks.

PPARG_f1	1.00E+00	4.57E-002	non-sign
COT1_f1	1.00E+00	4.60E-002	non-sign
TBX5_si	1.00E+00	4.64E-002	non-sign
NR2C2_f1	1.00E+00	4.68E-002	non-sign
STAT3_si	1.00E+00	4.71E-002	non-sign
ETV5_f1	1.00E+00	4.75E-002	non-sign
HAND1_si	1.00E+00	4.78E-002	non-sign
PEBB_f1	1.00E+00	4.82E-002	non-sign
NR5A2_f1	1.00E+00	4.86E-002	non-sign
SUH_f1	1.00E+00	4.89E-002	non-sign
NKX21_f1	1.00E+00	4.93E-002	non-sign
TCF7_f1	1.00E+00	4.96E-002	non-sign
SMAD4_si	1.00E+00	5.00E-002	non-sign
PPARA_f2	1.00E+00	5.04E-002	non-sign

Table S2. Transcription factors overrepresented in promoters of protein-coding and lncRNA genes for complete promoter set (CPS) and repeat filtered promoter set (REFPS) and support provided by DNAseI and ChIP-Seq peaks.

Complete Promoter Set (CPS)			
**Known TFBSs from HOCOMOCO overrepresented in promoters of lncRNA genes and their support by ENCODE ChIP-Seq peaks**			
**HOCOMOCO motif name**	**Right-side Fisher's exact test**	**Benjamini–Hochberg FDR correction (0.05)**	**FDR significance**
GATA3_si	1.33E-004	1.19E-003	ok
ARI3A_do	6.06E-004	2.38E-003	ok
MEF2A_f1	2.41E-003	3.57E-003	ok
FOSL1_f2	5.53E-003	4.76E-003	non-sign
BATF_si	2.12E-002	5.95E-003	non-sign
PO5F1_do	8.55E-002	7.14E-003	non-sign
FOXA1_f1	2.61E-001	8.33E-003	non-sign
ARI3A_f1	3.95E-001	9.52E-003	non-sign
GATA2_si	4.56E-001	1.07E-002	non-sign
IRF4_si	4.68E-001	1.19E-002	non-sign
GCR_si	6.66E-001	1.31E-002	non-sign
FOXA2_f1	6.94E-001	1.43E-002	non-sign
JUNB_f1	7.47E-001	1.55E-002	non-sign
NFE2_f2	8.73E-001	1.67E-002	non-sign
FOSL2_f1	9.06E-001	1.79E-002	non-sign
FOXP2_si	9.61E-001	1.90E-002	non-sign
HSF1_f2	9.71E-001	2.02E-002	non-sign
STAT2_f1	9.89E-001	2.14E-002	non-sign
PRDM1_f1	9.92E-001	2.26E-002	non-sign
CEBPB_f1	9.94E-001	2.38E-002	non-sign
GCR_do	9.97E-001	2.50E-002	non-sign

Table S2. Transcription factors overrepresented in promoters of protein-coding and lncRNA genes for complete promoter set (CPS) and repeat filtered promoter set (REFPS) and support provided by DNAseI and ChIP-Seq peaks.

FOXM1_f1		9.98E-001	2.62E-002	non-sign
CEBPD_f1		9.99E-001	2.74E-002	non-sign
IRF3_f1		1.00E+000	2.86E-002	non-sign
NFAC1_do		1.00E+000	2.98E-002	non-sign
TFE2_f2		1.00E+000	3.10E-002	non-sign
JUN_f1		1.00E+000	3.21E-002	non-sign
ERR1_f1		1.00E+000	3.33E-002	non-sign
HNF4A_f1		1.00E+000	3.45E-002	non-sign
IRF1_si		1.00E+000	3.57E-002	non-sign
JUND_f1		1.00E+000	3.69E-002	non-sign
NFAC1_si		1.00E+000	3.81E-002	non-sign
NR2C2_f1		1.00E+000	3.93E-002	non-sign
COT2_f1		1.00E+000	4.05E-002	non-sign
COT2_f2		1.00E+000	4.17E-002	non-sign
SPI1_si		1.00E+000	4.29E-002	non-sign
STAT1_f2		1.00E+000	4.40E-002	non-sign
STAT3_si		1.00E+000	4.52E-002	non-sign
STA5A_do		1.00E+000	4.64E-002	non-sign
TBP_f1		1.00E+000	4.76E-002	non-sign
TF7L2_f1		1.00E+000	4.88E-002	non-sign
ZEB1_do		1.00E+000	5.00E-002	non-sign
ZN384_f1		1.00E+000	5.12E-002	non-sign

Table S2. Transcription factors overrepresented in promoters of protein-coding and lncRNA genes for complete promoter set (CPS) and repeat filtered promoter set (REFPS) and support provided by DNAseI and ChIP-Seq peaks.

Repeat-filtered promoter set (REFPS)				
**Known TFBSs from HOCOMOCO overrepresented in promoters of mRNA genes**				
**HOCOMOCO motif name**	**P-Value (fisher's Right-side Exact test)**	**Benjamini–Hochberg FDR correction (0.05)**	**FDR significance**	**Distance from the ideal motif family**
EGR1_f2	0.00000E+00	6.849315E-04	ok	0.403500
SP4_f1	0.00000E+00	1.369863E-03	ok	0.425080
MECP2_f1	0.00000E+00	2.054795E-03	ok	0.441990
WT1_f1	0.00000E+00	2.739726E-03	ok	0.452660
SP2_si	0.00000E+00	3.424658E-03	ok	0.473310
KLF6_si	0.00000E+00	4.109589E-03	ok	0.473310
AP2B_f1	0.00000E+00	4.794521E-03	ok	0.477240
ZN148_si	0.00000E+00	5.479452E-03	ok	0.478110
MAZ_f1	0.00000E+00	6.164384E-03	ok	0.482840
ZFX_f1	0.00000E+00	6.849315E-03	ok	0.491760
EGR2_si	0.00000E+00	7.534247E-03	ok	0.492800
ZN219_f1	0.00000E+00	8.219178E-03	ok	0.499520
E2F6_f1	0.00000E+00	8.904110E-03	ok	0.503140
CTCF_f2	0.00000E+00	9.589041E-03	ok	0.508070
E2F1_f2	0.00000E+00	1.027397E-02	ok	0.513620

Table S2. Transcription factors overrepresented in promoters of protein-coding and lncRNA genes for complete promoter set (CPS) and repeat filtered promoter set (REFPS) and support provided by DNAseI and ChIP-Seq peaks.

GABPA_f1		0.00000E+00	1.095890E-02	ok	0.515900
SP3_f1		0.00000E+00	1.164384E-02	ok	0.523120
NRF1_f1		0.00000E+00	1.232877E-02	ok	0.523730
SP1_f1		0.00000E+00	1.301370E-02	ok	0.537280
SP1_f2		0.00000E+00	1.369863E-02	ok	0.543150
KLF4_f2		0.00000E+00	1.438356E-02	ok	0.559620
MBD2_si		0.00000E+00	1.506849E-02	ok	0.567330
ZBT7B_si		0.00000E+00	1.575342E-02	ok	0.631070
KLF15_f1		1.14570E-295	1.643836E-02	ok	0.542160
PURA_f1		1.98528E-293	1.712329E-02	ok	0.525420
ZBT7A_f1		1.30754E-292	1.780822E-02	ok	0.521200
AP2A_f2		7.80211E-287	1.849315E-02	ok	0.579070
GABP1+GABP2_f1		2.99531E-280	1.917808E-02	ok	0.556040
PLAG1_f1		1.07371E-279	1.986301E-02	ok	0.538860
AP2C_f1		4.15751E-264	2.054795E-02	ok	0.586050
ZIC1_f1		7.82934E-224	2.123288E-02	ok	0.565830
NR0B1_si		4.72066E-214	2.191781E-02	ok	0.591500
E2F4_do		9.05822E-213	2.260274E-02	ok	0.573440
HEN1_si		1.06362E-199	2.328767E-02	ok	0.699810
ELK1_f1		4.92277E-198	2.397260E-02	ok	0.577700
HES1_f1		5.40751E-192	2.465753E-02	ok	0.631840
TFCP2_f1		4.74155E-189	2.534247E-02	ok	0.561320
FLI1_f1		1.53460E-185	2.602740E-02	ok	0.569760
PLAG1_si		1.85878E-173	2.671233E-02	ok	0.575280
ETS2_f1		3.81338E-159	2.739726E-02	ok	0.637820
GLIS3_f1		2.64906E-140	2.808219E-02	ok	0.693920
COE1_f2		2.82958E-131	2.876712E-02	ok	0.689600
SRBP2_f1		2.01211E-127	2.945205E-02	ok	0.603350
PLAL1_si		6.07911E-126	3.013699E-02	ok	0.623620

Table S2. Transcription factors overrepresented in promoters of protein-coding and lncRNA genes for complete promoter set (CPS) and repeat filtered promoter set (REFPS) and support provided by DNAseI and ChIP-Seq peaks.

MZF1_f1	2.43239E-125	3.082192E-02	ok	0.588580
ELF1_f1	1.07890E-122	3.150685E-02	ok	0.588960
PAX5_f1	1.92092E-112	3.219178E-02	ok	0.598900
KLF1_f1	2.93803E-109	3.287671E-02	ok	0.695320
ARNT2_si	6.39249E-108	3.356164E-02	ok	0.583080
REST_f1	1.35066E-94	3.424658E-02	ok	0.679740
ZIC2_f1	1.20110E-92	3.493151E-02	ok	0.587740
NFKB1_f1	1.02324E-79	3.561644E-02	ok	0.618940
ZEP2_si	6.83685E-71	3.630137E-02	ok	0.656770
KAISO_f1	2.83333E-63	3.698630E-02	ok	0.647570
GT2D1_f1	3.73263E-54	3.767123E-02	ok	0.605100
NFYC_f1	8.57934E-45	3.835616E-02	ok	0.668320
MYC_f1	8.91831E-45	3.904110E-02	ok	0.691000
YBOX1_f2	2.24613E-43	3.972603E-02	ok	0.665770
ZIC3_f1	5.45469E-40	4.041096E-02	ok	0.614590
SMAD2_si	7.02700E-37	4.109589E-02	ok	0.670980
ETS1_si	2.52465E-33	4.178082E-02	ok	0.673860
MYOG_f1	7.37488E-33	4.246575E-02	ok	0.689350
SRBP1_f2	8.00258E-29	4.315068E-02	ok	0.607810
PAX5_si	9.07160E-27	4.383562E-02	ok	0.638120
NFYA_f1	3.74366E-26	4.452055E-02	ok	0.679420
MAFA_f1	4.04977E-25	4.520548E-02	ok	0.683430
TF65_f2	6.94108E-25	4.589041E-02	ok	0.629000
NFYB_f1	2.27683E-23	4.657534E-02	ok	0.667000
ESR1_do	6.70493E-23	4.726027E-02	ok	0.624980
NDF1_f1	6.21315E-22	4.794521E-02	ok	0.678930
REL_do	2.15009E-18	4.863014E-02	ok	0.693470
MYF6_f1	1.11326E-10	4.931507E-02	ok	0.695140
NFIA+NFIB+NFIC+NFIX_f2	3.50070E-10	5.000000E-02	ok	0.678860

Table S2. Transcription factors overrepresented in promoters of protein-coding and lncRNA genes for complete promoter set (CPS) and repeat-filtered promoter set (REFPS) and support provided by DNAseI and ChIP-Seq peaks.

Repeat-filtered promoter set (REFPS)									
Known TFBSs from HOCOMOCO overrepresented in promoters of lncRNA genes					Ab initio motif families overrepresented in promoters of lncRNA genes and similar TFBSs from HOCOMOCO				
HOCOMOCO motif name	P-Value (fisher's Right-side Exact test)	Benjamini–Hochberg FDR correction (0.05)	FDR significance	Distance from the ideal motif family	HOCOMOCO motif name	Ab initio motif family ID	Q-Value of similarity	P-Value of over-representation for ab initio motif using Fisher's right side exact test	Distance of ab initio motif from ideal motif family
HMGA1_f1	0.00E+000	3.906250E-04	ok	5.32E-001	FUBP1_f1	194	2.75E-002	0.000000	0.517452
SRY_f1	0.00E+000	7.812500E-04	ok	5.38E-001	FOXJ3_si	194	2.75E-002	0.000000	0.517452
FOXO1_si	0.00E+00	1.171875E-03	ok	5.41E-001	FOXO1_si	194	2.75E-002	0.000000	0.517452
FUBP1_f1	1.93E-295	1.562500E-03	ok	5.54E-001					
FOXA2_f1	6.96E-278	1.953125E-03	ok	5.60E-001					
FOXA1_f1	2.78E-245	2.343750E-03	ok	5.65E-001					
SOX2_f1	5.09E-294	2.734375E-03	ok	5.69E-001					
BPTF_si	1.26E-248	3.125000E-03	ok	5.71E-001					
FOXM1_f1	7.80E-277	3.515625E-03	ok	5.72E-001					
PO5F1_do	3.62E-296	3.906250E-03	ok	5.73E-001					
CDX2_f1	4.42E-293	4.296875E-03	ok	5.74E-001					
SOX5_f1	0.00E+000	4.687500E-03	ok	5.74E-001					
FOXO3_si	6.89E-244	5.078125E-03	ok	5.76E-001					
FOXP2_si	2.27E-201	5.468750E-03	ok	5.85E-001					
IRF7_f1	3.73E-175	5.859375E-03	ok	5.88E-001					
PO2F1_f1	1.49E-256	6.250000E-03	ok	5.92E-001					
NR2E3_f1	8.59E-234	6.640625E-03	ok	5.92E-001					
PO3F2_si	0.00E+000	7.031250E-03	ok	6.00E-001					
TBP_f1	5.63E-233	7.421875E-03	ok	6.07E-001					
IRF1_si	4.31E-131	7.812500E-03	ok	6.07E-001					
SOX10_si	1.18E-166	8.203125E-03	ok	6.08E-001					
IRF4_si	1.33E-289	8.593750E-03	ok	6.09E-001					
FOXD3_f1	2.52E-229	8.984375E-03	ok	6.13E-001					
STAT2_f1	1.25E-169	9.375000E-03	ok	6.16E-001					
NFAC1_si	2.54E-135	9.765625E-03	ok	6.19E-001					
GCR_si	4.58E-093	1.015625E-02	ok	6.21E-001					
HXD13_f1	1.28E-254	1.054688E-02	ok	6.23E-001					
MSX2_f1	1.40E-284	1.093750E-02	ok	6.23E-001					
PRGR_f1	5.93E-89	1.132813E-02	ok	6.24E-001					
EVI1_f1	4.12E-233	1.171875E-02	ok	6.25E-001					
HXD10_f1	1.54E-250	1.210938E-02	ok	6.25E-001					
IRF3_f1	7.79E-89	1.250000E-02	ok	6.26E-001					
TF7L2_f1	9.21E-89	1.289063E-02	ok	6.27E-001					
CDX1_f1	1.30E-197	1.328125E-02	ok	6.32E-001					

Table S2. Transcription factors overrepresented in promoters of protein-coding and lncRNA genes for complete promoter set (CPS) and repeat-filtered promoter set (REFPS) and support provided by DNAseI and ChIP-Seq peaks.

FOXJ2_f1	6.62E-267	1.367188E-02	ok	6.34E-001	
PPARG_si	3.41E-075	1.406250E-02	ok	6.35E-001	
CEBPA_do	2.24E-157	1.445313E-02	ok	6.35E-001	
HXD4_f1	2.53E-243	1.484375E-02	ok	6.35E-001	
ZN384_f1	5.51E-099	1.523438E-02	ok	6.37E-001	
IRF2_f1	1.90E-76	1.562500E-02	ok	6.37E-001	
HSF1_f2	2.61E-069	1.601563E-02	ok	6.37E-001	
LEF1_f1	3.75E-91	1.640625E-02	ok	6.40E-001	
BCL6_f1	6.47E-096	1.679688E-02	ok	6.40E-001	
NFAC2_f1	3.68E-057	1.718750E-02	ok	6.42E-001	
SOX13_f1	4.47E-196	1.757813E-02	ok	6.42E-001	
ERR1_f1	3.87E-57	1.796875E-02	ok	6.44E-001	
NF2L2_si	9.84E-060	1.835938E-02	ok	6.44E-001	
HNF1A_f1	4.50E-251	1.875000E-02	ok	6.45E-001	
FOXJ3_si	1.37E-275	1.914063E-02	ok	6.45E-001	
RXRB_f1	1.68E-065	1.953125E-02	ok	6.45E-001	
VDR_f1	4.88E-050	1.992188E-02	ok	6.46E-001	
NFAC4_f1	4.90E-052	2.031250E-02	ok	6.46E-001	
PRDM1_f1	1.49E-067	2.070313E-02	ok	6.47E-001	
NR1I3_si	4.98E-099	2.109375E-02	ok	6.47E-001	
ERR2_f1	5.42E-056	2.148438E-02	ok	6.49E-001	
NKX31_si	5.36E-299	2.187500E-02	ok	6.49E-001	
GFI1_f1	2.58E-091	2.226563E-02	ok	6.49E-001	
PARP1_si	1.47E-043	2.265625E-02	ok	6.50E-001	
NFAC3_f1	1.12E-048	2.304688E-02	ok	6.50E-001	
IRF8_si	9.99E-044	2.343750E-02	ok	6.51E-001	
STAT6_do	2.63E-080	2.382813E-02	ok	6.52E-001	
RARG_f1	1.06E-064	2.421875E-02	ok	6.52E-001	
ELF5_f1	1.22E-041	2.460938E-02	ok	6.54E-001	
NKX25_f1	2.76E-088	2.500000E-02	ok	6.54E-001	
SOX15_f1	1.25E-118	2.539063E-02	ok	6.54E-001	
FOXD1_si	1.26E-165	2.578125E-02	ok	6.55E-001	
JUND_f1	1.94E-089	2.617188E-02	ok	6.55E-001	
NR1I2_si	2.14E-096	2.656250E-02	ok	6.56E-001	
FOSL2_f1	9.58E-078	2.695313E-02	ok	6.57E-001	
GFI1B_f1	3.54E-103	2.734375E-02	ok	6.57E-001	
MITF_f1	6.86E-64	2.773438E-02	ok	6.57E-001	
COT2_f1	1.55E-062	2.812500E-02	ok	6.60E-001	
RUNX2_f1	5.60E-039	2.851563E-02	ok	6.60E-001	
GATA6_f2	1.58E-170	2.890625E-02	ok	6.61E-001	
FOXF2_f1	2.41E-190	2.929688E-02	ok	6.62E-001	
GCR_do	8.85E-30	2.968750E-02	ok	6.62E-001	
RUNX1_f1	1.11E-52	3.007813E-02	ok	6.62E-001	
ATF5_si	4.44E-027	3.046875E-02	ok	6.64E-001	
PPARA_f1	1.77E-49	3.085938E-02	ok	6.65E-001	
BATF_si	1.98E-120	3.125000E-02	ok	6.65E-001	
PIT1_f1	5.91E-225	3.164063E-02	ok	6.65E-001	
SOX9_f1	3.98E-146	3.203125E-02	ok	6.66E-001	
JUNB_f1	1.35E-105	3.242188E-02	ok	6.67E-001	

Table S2. Transcription factors overrepresented in promoters of protein-coding and lncRNA genes for complete promoter set (CPS) and repeat-filtered promoter set (REFPS) and support provided by DNAseI and ChIP-Seq peaks.

NFE2_f2	2.01E-031	3.281250E-02	ok	6.67E-001	
JUN_f1	7.16E-079	3.320313E-02	ok	6.69E-001	
PBX1_do	3.07E-169	3.359375E-02	ok	6.69E-001	
GATA4_f1	1.17E-129	3.398438E-02	ok	6.69E-001	
FOXO4_f1	1.11E-156	3.437500E-02	ok	6.70E-001	
STA5A_do	4.32E-045	3.476563E-02	ok	6.71E-001	
FOXP3_f1	2.78E-208	3.515625E-02	ok	6.72E-001	
CEBPD_f1	1.87E-114	3.554688E-02	ok	6.73E-001	
ZEB1_do	1.54E-018	3.593750E-02	ok	6.73E-001	
TEAD1_f1	1.04E-098	3.632813E-02	ok	6.74E-001	
SPIB_f1	2.08E-039	3.671875E-02	ok	6.74E-001	
HSF2_f2	3.91E-070	3.710938E-02	ok	6.75E-001	
MAFG_si	1.01E-091	3.750000E-02	ok	6.75E-001	
UBIP1_f1	7.00E-079	3.789063E-02	ok	6.77E-001	
TGIF1_si	1.37E-042	3.828125E-02	ok	6.77E-001	
TFE2_f2	1.29E-032	3.867188E-02	ok	6.77E-001	
PITX2_si	2.77E-124	3.906250E-02	ok	6.78E-001	
FOSL1_f2	1.75E-068	3.945313E-02	ok	6.79E-001	
THA_f1	1.12E-026	3.984375E-02	ok	6.80E-001	
ARI3A_f1	2.71E-207	4.023438E-02	ok	6.80E-001	
FOXA3_f1	1.59E-165	4.062500E-02	ok	6.82E-001	
AIRE_f2	9.35E-106	4.101563E-02	ok	6.82E-001	
ARI5B_f1	7.25E-197	4.140625E-02	ok	6.83E-001	
GATA3_si	2.54E-112	4.179688E-02	ok	6.85E-001	
THB_f1	1.87E-024	4.218750E-02	ok	6.85E-001	
ESR2_si	7.10E-015	4.257813E-02	ok	6.85E-001	
CRX_si	1.01E-139	4.296875E-02	ok	6.85E-001	
MAF_f1	1.10E-011	4.335938E-02	ok	6.85E-001	
COT2_f2	1.32E-010	4.375000E-02	ok	6.85E-001	
COT1_si	6.38E-030	4.414063E-02	ok	6.86E-001	
THA_f2	3.23E-010	4.453125E-02	ok	6.86E-001	
TBX5_si	1.48E-037	4.492188E-02	ok	6.86E-001	
MEF2A_f1	1.02E-214	4.531250E-02	ok	6.87E-001	
STF1_f1	2.68E-013	4.570313E-02	ok	6.87E-001	
GATA2_si	2.29E-099	4.609375E-02	ok	6.89E-001	
OTX1_f1	2.24E-104	4.648438E-02	ok	6.91E-001	
CEBPB_f1	6.55E-079	4.687500E-02	ok	6.92E-001	
COT1_f1	6.76E-012	4.726563E-02	ok	6.93E-001	
ALX1_si	1.08E-243	4.765625E-02	ok	6.93E-001	
NF2L1_f1	7.08E-067	4.804688E-02	ok	6.93E-001	
STAT4_si	2.84E-056	4.843750E-02	ok	6.94E-001	
TCF7_f1	2.86E-039	4.882813E-02	ok	6.94E-001	
HNF1B_f1	9.00E-188	4.921875E-02	ok	6.95E-001	
ETV5_f1	1.06E-010	4.960938E-02	ok	6.98E-001	
HAND1_si	4.67E-008	5.000000E-02	ok	6.99E-001	

Table S2. Transcription factors overrepresented in promoters of protein-coding and lncRNA genes for complete promoter set (CPS) and repeat filtered promoter set (REFPS) and support provided by DNAseI and ChIP-Seq peaks.

Repeat-filtered promoter set (REFPS)

Known TFBSs from HOCOMOCO overrepresented in promoters of lncRNA genes and their support by DNAaseI

HOCOMOCO motif name	Right-side Fisher's exact test	Benjamini–Hochberg FDR correction (0.05)	FDR significance
ALX1_si	1.02E-012	3.91E-004	ok
NKX31_si	1.23E-012	7.81E-004	ok
SOX5_f1	2.91E-10	1.17E-003	ok
FOXJ3_si	3.62E-010	1.56E-003	ok
SOX2_f1	1.45E-09	1.95E-003	ok
PIT1_f1	3.40E-009	2.34E-003	ok
PO5F1_do	7.45E-09	2.73E-003	ok
FOXJ2_f1	9.53E-008	3.13E-003	ok
HMGA1_f1	1.99E-07	3.52E-003	ok
PO3F2_si	3.06E-007	3.91E-003	ok
FOXO1_si	9.98E-07	4.30E-003	ok
HXD4_f1	5.63E-006	4.69E-003	ok
FOXP3_f1	1.34E-005	5.08E-003	ok
HNF1A_f1	1.47E-005	5.47E-003	ok
MSX2_f1	1.88E-005	5.86E-003	ok
MEF2A_f1	6.88E-005	6.25E-003	ok
PO2F1_f1	9.24E-005	6.64E-003	ok
HXD10_f1	9.53E-005	7.03E-003	ok
SRY_f1	1.54E-04	7.42E-003	ok
EVI1_f1	0.00044778	7.81E-003	ok
CDX2_f1	5.89E-04	8.20E-003	ok

Table S2. Transcription factors overrepresented in promoters of protein-coding and lncRNA genes for complete promoter set (CPS) and repeat filtered promoter set (REFPS) and support provided by DNAseI and ChIP-Seq peaks.

ARI3A_f1	1.51E-003	8.59E-003	ok
HXD13_f1	0.0016333	8.98E-003	ok
FOXD3_f1	2.75E-003	9.38E-003	ok
FOXF2_f1	3.03E-003	9.77E-003	ok
HNF1B_f1	4.85E-003	1.02E-002	ok
FOXA3_f1	5.31E-003	1.05E-002	ok
PBX1_do	5.65E-003	1.09E-002	ok
SOX13_f1	0.0078952	1.13E-002	ok
FUBP1_f1	9.68E-03	1.17E-002	ok
FOXO3_si	1.42E-02	1.21E-002	non-sign
FOXA2_f1	1.95E-02	1.25E-002	non-sign
FOXM1_f1	2.20E-02	1.29E-002	non-sign
GATA6_f2	2.95E-002	1.33E-002	non-sign
BATF_si	3.02E-002	1.37E-002	non-sign
NR2E3_f1	3.38E-002	1.41E-002	non-sign
ARI5B_f1	5.70E-002	1.45E-002	non-sign
SOX9_f1	9.34E-002	1.48E-002	non-sign
FOXO4_f1	1.24E-001	1.52E-002	non-sign
BPTF_si	3.22E-01	1.56E-002	non-sign
FOXD1_si	5.54E-001	1.60E-002	non-sign
FOXP2_si	5.69E-01	1.64E-002	non-sign
TBP_f1	5.91E-001	1.68E-002	non-sign
JUNB_f1	6.22E-001	1.72E-002	non-sign
CEBPA_do	6.50E-001	1.76E-002	non-sign
CDX1_f1	0.65017	1.80E-002	non-sign
SOX10_si	6.58E-001	1.84E-002	non-sign
FOXA1_f1	7.83E-01	1.88E-002	non-sign
CRX_si	9.13E-001	1.91E-002	non-sign
SOX15_f1	9.31E-001	1.95E-002	non-sign
TEAD1_f1	9.40E-001	1.99E-002	non-sign
JUN_f1	9.70E-001	2.03E-002	non-sign
MAFG_si	9.80E-001	2.07E-002	non-sign
GATA4_f1	9.87E-001	2.11E-002	non-sign
AIRE_f2	9.91E-001	2.15E-002	non-sign

Table S2. Transcription factors overrepresented in promoters of protein-coding and lncRNA genes for complete promoter set (CPS) and repeat filtered promoter set (REFPS) and support provided by DNAseI and ChIP-Seq peaks.

JUND_f1	9.93E-001	2.19E-002	non-sign
PITX2_si	9.97E-001	2.23E-002	non-sign
CEBPD_f1	9.97E-001	2.27E-002	non-sign
GATA2_si	9.98E-001	2.30E-002	non-sign
GATA3_si	9.98E-001	2.34E-002	non-sign
NR1I3_si	1.00E+000	2.38E-002	non-sign
NFAC1_si	1.00E+000	2.42E-002	non-sign
NR1I2_si	1.00E+000	2.46E-002	non-sign
GFI1B_f1	1.00E+000	2.50E-002	non-sign
FOSL1_f2	1.00E+000	2.54E-002	non-sign
IRF7_f1	1.00E+000	2.58E-002	non-sign
IRF1_si	1.00E+000	2.62E-002	non-sign
IRF4_si	1.00E+000	2.66E-002	non-sign
STAT2_f1	1.00E+000	2.70E-002	non-sign
GCR_si	1.00E+000	2.73E-002	non-sign
PRGR_f1	1	2.77E-002	non-sign
IRF3_f1	1.00E+000	2.81E-002	non-sign
TF7L2_f1	1.00E+000	2.85E-002	non-sign
PPARG_si	1.00E+000	2.89E-002	non-sign
ZN384_f1	1	2.93E-002	non-sign
IRF2_f1	1	2.97E-002	non-sign
HSF1_f2	1	3.01E-002	non-sign
LEF1_f1	1.00E+000	3.05E-002	non-sign
BCL6_f1	1	3.09E-002	non-sign
NFAC2_f1	1.00E+000	3.13E-002	non-sign
ERR1_f1	1	3.16E-002	non-sign
NF2L2_si	1.00E+000	3.20E-002	non-sign
RXRB_f1	1.00E+000	3.24E-002	non-sign
VDR_f1	1.00E+000	3.28E-002	non-sign
NFAC4_f1	1.00E+000	3.32E-002	non-sign
PRDM1_f1	1.00E+000	3.36E-002	non-sign
ERR2_f1	1.00E+000	3.40E-002	non-sign
GFI1_f1	1.00E+000	3.44E-002	non-sign
PARP1_si	1.00E+000	3.48E-002	non-sign

Table S2. Transcription factors overrepresented in promoters of protein-coding and lncRNA genes for complete promoter set (CPS) and repeat filtered promoter set (REFPS) and support provided by DNAseI and ChIP-Seq peaks.

NFAC3_f1		1.00E+000	3.52E-002	non-sign
IRF8_si		1.00E+000	3.55E-002	non-sign
STAT6_do		1.00E+000	3.59E-002	non-sign
RARG_f1		1.00E+000	3.63E-002	non-sign
ELF5_f1		1.00E+000	3.67E-002	non-sign
NKX25_f1		1.00E+000	3.71E-002	non-sign
FOSL2_f1		1.00E+000	3.75E-002	non-sign
MITF_f1		1.00E+000	3.79E-002	non-sign
COT2_f1		1.00E+000	3.83E-002	non-sign
RUNX2_f1		1.00E+000	3.87E-002	non-sign
GCR_do		1.00E+000	3.91E-002	non-sign
RUNX1_f1		1.00E+000	3.95E-002	non-sign
ATF5_si		1.00E+000	3.98E-002	non-sign
PPARA_f1		1.00E+000	4.02E-002	non-sign
NFE2_f2		1.00E+000	4.06E-002	non-sign
STA5A_do		1.00E+000	4.10E-002	non-sign
ZEB1_do		1.00E+000	4.14E-002	non-sign
SPIB_f1		1.00E+000	4.18E-002	non-sign
HSF2_f2		1.00E+000	4.22E-002	non-sign
UBIP1_f1		1.00E+000	4.26E-002	non-sign
TGIF1_si		1.00E+000	4.30E-002	non-sign
TFE2_f2		1.00E+000	4.34E-002	non-sign
THA_f1		1.00E+000	4.38E-002	non-sign
THB_f1		1.00E+000	4.41E-002	non-sign
ESR2_si		1.00E+000	4.45E-002	non-sign
MAF_f1		1.00E+000	4.49E-002	non-sign
COT2_f2		1.00E+000	4.53E-002	non-sign
COT1_si		1.00E+000	4.57E-002	non-sign
THA_f2		1.00E+000	4.61E-002	non-sign
TBX5_si		1.00E+000	4.65E-002	non-sign
STF1_f1		1.00E+000	4.69E-002	non-sign
OTX1_f1		1.00E+000	4.73E-002	non-sign
CEBPB_f1		1.00E+000	4.77E-002	non-sign
COT1_f1		1.00E+000	4.80E-002	non-sign

Table S2. Transcription factors overrepresented in promoters of protein-coding and lncRNA genes for complete promoter set (CPS) and repeat filtered promoter set (REFPS) and support provided by DNAseI and ChIP-Seq peaks.

NF2L1_f1	1.00E+000	4.84E-002	non-sign
STAT4_si	1.00E+000	4.88E-002	non-sign
TCF7_f1	1.00E+000	4.92E-002	non-sign
ETV5_f1	1.00E+000	4.96E-002	non-sign
HAND1_si	1.00E+000	5.00E-002	non-sign

Table S2. Transcription factors overrepresented in promoters of protein-coding and lncRNA genes for complete promoter set (CPS) and repeat filtered promoter set (REFPS) and support provided by DNAseI and ChIP-Seq peaks.

Repeat-filtered promoter set (REFPS)			
**Known TFBSs from HOCOMOCO overrepresented in promoters of lncRNA genes and their support by ChIP-Seq peaks**			
**HOCOMOCO motif name**	**Right-side Fisher's exact test**	**Benjamini–Hochberg FDR correction (0.05)**	**FDR significance**
PO5F1_do	0.82388	1.32E-003	non-sign
ARI3A_do	1	2.63E-003	non-sign
ARI3A_f1	1	3.95E-003	non-sign
BATF_si	1	5.26E-003	non-sign
JUN_f1	1	6.58E-003	non-sign
CEBPB_f1	1	7.89E-003	non-sign
CEBPD_f1	1	9.21E-003	non-sign
ERR1_f1	1	1.05E-002	non-sign
FOSL1_f2	1	1.18E-002	non-sign
FOSL2_f1	1	1.32E-002	non-sign
FOXA1_f1	1	1.45E-002	non-sign
FOXA2_f1	1	1.58E-002	non-sign
FOXM1_f1	1	1.71E-002	non-sign
FOXP2_si	1	1.84E-002	non-sign
GATA2_si	1	1.97E-002	non-sign
GATA3_si	1	2.11E-002	non-sign
HSF1_f2	1	2.24E-002	non-sign
IRF1_si	1	2.37E-002	non-sign
IRF3_f1	1	2.50E-002	non-sign
IRF4_si	1	2.63E-002	non-sign
JUNB_f1	1	2.76E-002	non-sign

Table S2. Transcription factors overrepresented in promoters of protein-coding and lncRNA genes for complete promoter set (CPS) and repeat filtered promoter set (REFPS) and support provided by DNAseI and ChIP-Seq peaks.

JUND_f1	1	2.89E-002	non-sign
MEF2A_f1	1	3.03E-002	non-sign
NFAC1_do	1	3.16E-002	non-sign
NFAC1_si	1	3.29E-002	non-sign
NFE2_f2	1	3.42E-002	non-sign
COT2_f1	1	3.55E-002	non-sign
COT2_f2	1	3.68E-002	non-sign
GCR_do	1	3.82E-002	non-sign
GCR_si	1	3.95E-002	non-sign
PRDM1_f1	1	4.08E-002	non-sign
STAT2_f1	1	4.21E-002	non-sign
STA5A_do	1	4.34E-002	non-sign
TBP_f1	1	4.47E-002	non-sign
TFE2_f2	1	4.61E-002	non-sign
TF7L2_f1	1	4.74E-002	non-sign
ZEB1_do	1	4.87E-002	non-sign
ZN384_f1	1	5.00E-002	non-sign
