# Supplementary material for: Promoter Analysis Reveals Globally Differential Regulation of Human Long Non-Coding RNA and Protein-Coding Genes
Source: PLoS One. 2014 Oct 2;9(10):e109443. doi: 10.1371/journal.pone.0109443 (PMC4183604; doi:10.1371/journal.pone.0109443)
Supplement: Table S3 — P-values of overrepresentation for chromatin states, CpG islands, repetitive elements and palindromes for complete promoter set (CPS) and repeat-filtered promoter set (REFPS). (PDF) [file pone.0109443.s009.pdf]

Table S3. P-values of overrepresentation for chromatin states, CpG islands, repetitive elements and palindromes for complete promoter set (CPS) and repeat-filtered promoter set (REFPS).

**Contingency table (protein-coding genes promoters vs lncRNA promoters)**	
a:Total protein-coding genes promoters having this mark	b:Total protein-coding genes promoters NOT having this mark
c:Total lncRNA promoters having this mark	d: Total lncRNA promoters NOT having this mark

**Contingency table (lncRNA promoters vs protein-coding genes promoters)**	
a:Total lncRNA promoters having this mark	b: Total lncRNA promoters NOT having this mark
c:Total protein-coding genes promoters having this mark	d:Total protein-coding genes promoters NOT having this mark

Complete promoter set (CPS)

Protein-coding gene promoters vs lncRNA promoters

**Repeat Elements**	**a**	**b**	**c**	**d**	**pvalue_Coding_over_NonCoding**		
	17462	1325	17530	957	1.00E+000		
**CGI**	**a**	**b**	**c**	**d**	**pvalue_Coding_over_NonCoding**		
	13179	5608	4354	14133	0.00E+000		
**Palindromes**	**a**	**b**	**c**	**d**	**pvalue_Coding_over_NonCoding**		
	18787	0	18487	0	1.00E+000		
**GM12878**	**a**	**b**	**c**	**d**	**pvalue_Coding_over_NonCoding**	**Benjamini–Hochberg FDR correction (0.05)**	**FDR significance**
AP: Active Promoter	9718	9069	2393	16094	0.00E+000	3.33E-003	sign
WP: Weak Promoter	7418	11369	2310	16177	0.00E+000	6.67E-003	sign
WE: Weak Enhancer	5720	13067	2274	16213	0.00E+000	1.00E-002	sign
IP: Inactive Promoter	1954	16833	685	17802	8.34E-146	1.33E-002	sign
SE: Strong Enhancer	2197	16590	989	17498	5.32E-109	1.67E-002	sign
PR: Polycomb Repressed	3289	15498	1980	16507	5.05E-080	2.00E-002	sign
SE: Strong Enhancer	675	18112	682	17805	7.00E-001	2.33E-002	non-sign
RP: Repetitive/Copy number variation	132	18655	158	18329	9.58E-001	2.67E-002	non-sign
WE: Weak Enhancer	1830	16957	1980	16507	9.99E-001	3.00E-002	non-sign
RP: Repetitive/Copy number variation	41	18746	89	18398	1.00E+000	3.33E-002	non-sign
I: Insulator	560	18227	867	17620	1.00E+000	3.67E-002	non-sign
TT: Transcriptional Transition	168	18619	301	18186	1.00E+000	4.00E-002	non-sign
TE: Transcriptional Elongation	262	18525	1198	17289	1.00E+000	4.33E-002	non-sign
WT: Weak Transcribed	2554	16233	2977	15510	1.00E+000	4.67E-002	non-sign
HC: Heterochromatin low signal	6693	12094	11527	6960	1.00E+000	5.00E-002	non-sign

LncRNA promoters vs protein-coding gene promoters

**Repeat Elements**	**a**	**b**	**c**	**d**	**pvalue_NonCoding_over_Coding**		
	17530	957	17462	1325	2.19E-014		
**CGI**	**a**	**b**	**c**	**d**	**pvalue_NonCoding_over_Coding**		
	4354	14133	13179	5608	1.00E+000		
**Palindromes**	**a**	**b**	**c**	**d**	**pvalue_NonCoding_over_Coding**		
	18487	0	18787	0	1.00E+000		
**GM12878**	**a**	**b**	**c**	**d**	**pvalue_NonCoding_over_Coding**	**Benjamini–Hochberg FDR correction (0.05)**	**FDR significance**
HC: Heterochromatin low signal	11527	6960	6693	12094	0.00E+000	3.33E-003	sign
TE: Transcriptional Elongation	1198	17289	262	18525	4.65E-152	6.67E-003	sign
I: Insulator	867	17620	560	18227	4.00E-018	1.00E-002	sign
WT: Weak Transcribed	2977	15510	2554	16233	5.24E-012	1.33E-002	sign
TT: Transcriptional Transition	301	18186	168	18619	1.09E-010	1.67E-002	sign
RP: Repetitive/Copy number variation	89	18398	41	18746	1.00E-005	2.00E-002	sign
WE: Weak Enhancer	1980	16507	1830	16957	1.06E-003	2.33E-002	sign
RP: Repetitive/Copy number variation	158	18329	132	18655	5.35E-002	2.67E-002	non-sign
SE: Strong Enhancer	682	17805	675	18112	3.20E-001	3.00E-002	non-sign
AP: Active Promoter	2393	16094	9718	9069	1.00E+000	3.33E-002	non-sign
WP: Weak Promoter	2310	16177	7418	11369	1.00E+000	3.67E-002	non-sign
IP: Inactive Promoter	685	17802	1954	16833	1.00E+000	4.00E-002	non-sign
SE: Strong Enhancer	989	17498	2197	16590	1.00E+000	4.33E-002	non-sign
WE: Weak Enhancer	2274	16213	5720	13067	1.00E+000	4.67E-002	non-sign
PR: Polycomb Repressed	1980	16507	3289	15498	1.00E+000	5.00E-002	non-sign

Table S3. P-values of overrepresentation for chromatin states, CpG islands, repetitive elements and palindromes for complete promoter set (CPS) and repeat-filtered promoter set (REFPS).

H1-hESC	a	b	c	d	pvalue_Coding_over_NonCoding	Benjamini–Hochberg FDR correction (0.05)	FDR significance
AP: Active Promoter	8775	10012	2091	16396	0.00E+000	3.33E-003	sign
WE: Weak Enhancer	7981	10806	3255	15232	0.00E+000	6.67E-003	sign
WP: Weak Promoter	10811	7976	2928	15559	0.00E+000	1.00E-002	sign
IP: Inactive Promoter	4073	14714	1614	16873	1.18E-272	1.33E-002	sign
PR: Polycomb Repressed	2397	16390	1421	17066	1.94E-059	1.67E-002	sign
SE: Strong Enhancer	792	17995	326	18161	3.30E-045	2.00E-002	sign
SE: Strong Enhancer	361	18426	365	18122	6.58E-001	2.33E-002	non-sign
RP: Repetitive/Copy number variation	34	18753	69	18418	1.00E+000	2.67E-002	non-sign
RP: Repetitive/Copy number variation	59	18728	106	18381	1.00E+000	3.00E-002	non-sign
HC: Heterochromatin low signal	6475	12312	10975	7512	1.00E+000	3.33E-002	non-sign
I: Insulator	719	18068	1324	17163	1.00E+000	3.67E-002	non-sign
TE: Transcriptional Elongation	133	18654	756	17731	1.00E+000	4.00E-002	non-sign
TT: Transcriptional Transition	233	18554	362	18125	1.00E+000	4.33E-002	non-sign
WE: Weak Enhancer	2201	16586	2767	15720	1.00E+000	4.67E-002	non-sign
WT: Weak Transcribed	3432	15355	4679	13808	1.00E+000	5.00E-002	non-sign

H1-hESC	a	b	c	d	pvalue_NonCoding_over_Coding	Benjamini–Hochberg FDR correction (0.05)	FDR significance
HC: Heterochromatin low signal	10975	7512	6475	12312	0.00E+000	3.33E-003	sign
TE: Transcriptional Elongation	756	17731	133	18654	2.64E-111	6.67E-003	sign
WT: Weak Transcribed	4679	13808	3432	15355	2.36E-061	1.00E-002	sign
I: Insulator	1324	17163	719	18068	3.75E-046	1.33E-002	sign
WE: Weak Enhancer	2767	15720	2201	16586	1.39E-020	1.67E-002	sign
TT: Transcriptional Transition	362	18125	233	18554	1.83E-008	2.00E-002	sign
RP: Repetitive/Copy number variation	106	18381	59	18728	1.02E-004	2.33E-002	sign
RP: Repetitive/Copy number variation	69	18418	34	18753	2.65E-004	2.67E-002	sign
SE: Strong Enhancer	365	18122	361	18426	3.70E-001	3.00E-002	non-sign
AP: Active Promoter	2091	16396	8775	10012	1.00E+000	3.33E-002	non-sign
WP: Weak Promoter	2928	15559	10811	7976	1.00E+000	3.67E-002	non-sign
IP: Inactive Promoter	1614	16873	4073	14714	1.00E+000	4.00E-002	non-sign
SE: Strong Enhancer	326	18161	792	17995	1.00E+000	4.33E-002	non-sign
WE: Weak Enhancer	3255	15232	7981	10806	1.00E+000	4.67E-002	non-sign
PR: Polycomb Repressed	1421	17066	2397	16390	1.00E+000	5.00E-002	non-sign

HMEC	a	b	c	d	pvalue_Coding_over_NonCoding	Benjamini–Hochberg FDR correction (0.05)	FDR significance
AP: Active Promoter	9765	9022	2273	16214	0.00E+000	3.33E-003	sign
WP: Weak Promoter	8552	10235	2404	16083	0.00E+000	6.67E-003	sign
SE: Strong Enhancer	4269	14518	1516	16971	0.00E+000	1.00E-002	sign
WE: Weak Enhancer	6754	12033	2790	15697	0.00E+000	1.33E-002	sign
IP: Inactive Promoter	1615	17172	535	17952	7.94E-129	1.67E-002	sign
PR: Polycomb Repressed	2112	16675	1340	17147	7.33E-041	2.00E-002	sign
SE: Strong Enhancer	1541	17246	1481	17006	2.55E-001	2.33E-002	non-sign
RP: Repetitive/Copy number variation	41	18746	81	18406	1.00E+000	2.67E-002	non-sign
RP: Repetitive/Copy number variation	24	18763	58	18429	1.00E+000	3.00E-002	non-sign
WE: Weak Enhancer	2508	16279	2937	15550	1.00E+000	3.33E-002	non-sign
I: Insulator	429	18358	705	17782	1.00E+000	3.67E-002	non-sign
TT: Transcriptional Transition	115	18672	229	18258	1.00E+000	4.00E-002	non-sign
TE: Transcriptional Elongation	189	18598	979	17508	1.00E+000	4.33E-002	non-sign
WT: Weak Transcribed	3292	15495	3998	14489	1.00E+000	4.67E-002	non-sign
HC: Heterochromatin low signal	7314	11473	11393	7094	1.00E+000	5.00E-002	non-sign

HMEC	a	b	c	d	pvalue_NonCoding_over_Coding	Benjamini–Hochberg FDR correction (0.05)	FDR significance
HC: Heterochromatin low signal	11393	7094	7314	11473	0.00E+000	3.33E-003	sign
TE: Transcriptional Elongation	979	17508	189	18598	8.23E-136	6.67E-003	sign
WT: Weak Transcribed	3998	14489	3292	15495	9.51E-024	1.00E-002	sign
I: Insulator	705	17782	429	18358	3.74E-018	1.33E-002	sign
WE: Weak Enhancer	2937	15550	2508	16279	2.23E-012	1.67E-002	sign
TT: Transcriptional Transition	229	18258	115	18672	1.27E-010	2.00E-002	sign
RP: Repetitive/Copy number variation	58	18429	24	18763	8.20E-005	2.33E-002	sign
RP: Repetitive/Copy number variation	81	18406	41	18746	1.29E-004	2.67E-002	sign
SE: Strong Enhancer	1481	17006	1541	17246	7.57E-001	3.00E-002	non-sign
AP: Active Promoter	2273	16214	9765	9022	1.00E+000	3.33E-002	non-sign
WP: Weak Promoter	2404	16083	8552	10235	1.00E+000	3.67E-002	non-sign
IP: Inactive Promoter	535	17952	1615	17172	1.00E+000	4.00E-002	non-sign
SE: Strong Enhancer	1516	16971	4269	14518	1.00E+000	4.33E-002	non-sign
WE: Weak Enhancer	2790	15697	6754	12033	1.00E+000	4.67E-002	non-sign
PR: Polycomb Repressed	1340	17147	2112	16675	1.00E+000	5.00E-002	non-sign

Table S3. P-values of overrepresentation for chromatin states, CpG islands, repetitive elements and palindromes for complete promoter set (CPS) and repeat-filtered promoter set (REFPS).

HSMM	a	b	c	d	pvalue_Coding_over_NonCoding	Benjamini–Hochberg FDR correction (0.05)	FDR significance
AP: Active Promoter	9865	8922	2392	16095	0.00E+000	3.33E-003	sign
WP: Weak Promoter	9949	8838	2749	15738	0.00E+000	6.67E-003	sign
WE: Weak Enhancer	5604	13183	2717	15770	2.63E-274	1.00E-002	sign
IP: Inactive Promoter	1675	17112	637	17850	5.22E-110	1.33E-002	sign
SE: Strong Enhancer	2293	16494	1281	17206	4.69E-068	1.67E-002	sign
PR: Polycomb Repressed	2792	15995	2055	16432	2.85E-027	2.00E-002	sign
RP: Repetitive/Copy number variation	191	18596	160	18327	7.24E-002	2.33E-002	non-sign
RP: Repetitive/Copy number variation	48	18739	84	18403	1.00E+000	2.67E-002	non-sign
SE: Strong Enhancer	770	18017	1285	17202	1.00E+000	3.00E-002	non-sign
WE: Weak Enhancer	1377	17410	2226	16261	1.00E+000	3.33E-002	non-sign
I: Insulator	594	18193	898	17589	1.00E+000	3.67E-002	non-sign
TT: Transcriptional Transition	333	18454	512	17975	1.00E+000	4.00E-002	non-sign
TE: Transcriptional Elongation	411	18376	1560	16927	1.00E+000	4.33E-002	non-sign
WT: Weak Transcribed	3588	15199	4140	14347	1.00E+000	4.67E-002	non-sign
HC: Heterochromatin low signal	6977	11810	10297	8190	1.00E+000	5.00E-002	non-sign

HSMM	a	b	c	d	pvalue_NonCoding_over_Coding	Benjamini–Hochberg FDR correction (0.05)	FDR significance
HC: Heterochromatin low signal	10297	8190	6977	11810	1.74E-284	3.33E-003	sign
TE: Transcriptional Elongation	1560	16927	411	18376	1.04E-169	6.67E-003	sign
WE: Weak Enhancer	2226	16261	1377	17410	4.87E-054	1.00E-002	sign
SE: Strong Enhancer	1285	17202	770	18017	5.50E-034	1.33E-002	sign
I: Insulator	898	17589	594	18193	3.44E-017	1.67E-002	sign
WT: Weak Transcribed	4140	14347	3588	15199	2.31E-015	2.00E-002	sign
TT: Transcriptional Transition	512	17975	333	18454	5.52E-011	2.33E-002	sign
RP: Repetitive/Copy number variation	84	18403	48	18739	7.88E-004	2.67E-002	sign
RP: Repetitive/Copy number variation	160	18327	191	18596	9.41E-001	3.00E-002	non-sign
AP: Active Promoter	2392	16095	9865	8922	1.00E+000	3.33E-002	non-sign
WP: Weak Promoter	2749	15738	9949	8838	1.00E+000	3.67E-002	non-sign
IP: Inactive Promoter	637	17850	1675	17112	1.00E+000	4.00E-002	non-sign
SE: Strong Enhancer	1281	17206	2293	16494	1.00E+000	4.33E-002	non-sign
WE: Weak Enhancer	2717	15770	5604	13183	1.00E+000	4.67E-002	non-sign
PR: Polycomb Repressed	2055	16432	2792	15995	1.00E+000	5.00E-002	non-sign

HUVEC	a	b	c	d	pvalue_Coding_over_NonCoding	Benjamini–Hochberg FDR correction (0.05)	FDR significance
AP: Active Promoter	9701	9086	2246	16241	0.00E+000	3.33E-003	sign
WP: Weak Promoter	7839	10948	2123	16364	0.00E+000	6.67E-003	sign
SE: Strong Enhancer	5604	13183	1967	16520	0.00E+000	1.00E-002	sign
WE: Weak Enhancer	5875	12912	2153	16334	0.00E+000	1.33E-002	sign
IP: Inactive Promoter	2142	16645	830	17657	1.81E-138	1.67E-002	sign
PR: Polycomb Repressed	4013	14774	2950	15537	3.36E-041	2.00E-002	sign
SE: Strong Enhancer	1274	17513	1251	17236	4.86E-001	2.33E-002	non-sign
RP: Repetitive/Copy number variation	77	18710	111	18376	9.96E-001	2.67E-002	non-sign
WE: Weak Enhancer	1884	16903	2026	16461	9.98E-001	3.00E-002	non-sign
RP: Repetitive/Copy number variation	81	18706	139	18348	1.00E+000	3.33E-002	non-sign
I: Insulator	650	18137	875	17612	1.00E+000	3.67E-002	non-sign
TT: Transcriptional Transition	129	18658	259	18228	1.00E+000	4.00E-002	non-sign
TE: Transcriptional Elongation	202	18585	904	17583	1.00E+000	4.33E-002	non-sign
WT: Weak Transcribed	2538	16249	3147	15340	1.00E+000	4.67E-002	non-sign
HC: Heterochromatin low signal	6862	11925	11112	7375	1.00E+000	5.00E-002	non-sign

HUVEC	a	b	c	d	pvalue_NonCoding_over_Coding	Benjamini–Hochberg FDR correction (0.05)	FDR significance
HC: Heterochromatin low signal	11112	7375	6862	11925	0.00E+000	3.33E-003	sign
TE: Transcriptional Elongation	904	17583	202	18585	3.12E-112	6.67E-003	sign
WT: Weak Transcribed	3147	15340	2538	16249	2.14E-021	1.00E-002	sign
TT: Transcriptional Transition	259	18228	129	18658	5.14E-012	1.33E-002	sign
I: Insulator	875	17612	650	18137	3.06E-010	1.67E-002	sign
RP: Repetitive/Copy number variation	139	18348	81	18706	3.25E-005	2.00E-002	sign
WE: Weak Enhancer	2026	16461	1884	16903	1.78E-003	2.33E-002	sign
RP: Repetitive/Copy number variation	111	18376	77	18710	5.73E-003	2.67E-002	sign
SE: Strong Enhancer	1251	17236	1274	17513	5.30E-001	3.00E-002	non-sign
AP: Active Promoter	2246	16241	9701	9086	1.00E+000	3.33E-002	non-sign
WP: Weak Promoter	2123	16364	7839	10948	1.00E+000	3.67E-002	non-sign
IP: Inactive Promoter	830	17657	2142	16645	1.00E+000	4.00E-002	non-sign
SE: Strong Enhancer	1967	16520	5604	13183	1.00E+000	4.33E-002	non-sign
WE: Weak Enhancer	2153	16334	5875	12912	1.00E+000	4.67E-002	non-sign
PR: Polycomb Repressed	2950	15537	4013	14774	1.00E+000	5.00E-002	non-sign

Table S3. P-values of overrepresentation for chromatin states, CpG islands, repetitive elements and palindromes for complete promoter set (CPS) and repeat-filtered promoter set (REFPS).

NHEK	a	b	c	d	pvalue_Coding_over_NonCoding	Benjamini–Hochberg FDR correction (0.05)	FDR significance
AP: Active Promoter	10169	8618	2521	15966	0.00E+000	3.33E-003	sign
WP: Weak Promoter	7024	11763	2108	16379	0.00E+000	6.67E-003	sign
WE: Weak Enhancer	5944	12843	2530	15957	0.00E+000	1.00E-002	sign
SE: Strong Enhancer	4150	14637	1693	16794	1.46E-265	1.33E-002	sign
IP: Inactive Promoter	2218	16569	889	17598	5.08E-136	1.67E-002	sign
PR: Polycomb Repressed	3475	15312	2695	15792	1.25E-024	2.00E-002	sign
RP: Repetitive/Copy number variation	59	18728	92	18395	9.98E-001	2.33E-002	non-sign
SE: Strong Enhancer	1170	17617	1303	17184	9.99E-001	2.67E-002	non-sign
RP: Repetitive/Copy number variation	19	18768	43	18444	9.99E-001	3.00E-002	non-sign
WE: Weak Enhancer	1647	17140	2304	16183	1.00E+000	3.33E-002	non-sign
I: Insulator	641	18146	1038	17449	1.00E+000	3.67E-002	non-sign
TT: Transcriptional Transition	213	18574	326	18161	1.00E+000	4.00E-002	non-sign
TE: Transcriptional Elongation	307	18480	1151	17336	1.00E+000	4.33E-002	non-sign
WT: Weak Transcribed	2952	15835	3587	14900	1.00E+000	4.67E-002	non-sign
HC: Heterochromatin low signal	6415	12372	10613	7874	1.00E+000	5.00E-002	non-sign

NHEK	a	b	c	d	pvalue_NonCoding_over_Coding	Benjamini–Hochberg FDR correction (0.05)	FDR significance
HC: Heterochromatin low signal	10613	7874	6415	12372	0.00E+000	3.33E-003	sign
TE: Transcriptional Elongation	1151	17336	307	18480	1.37E-122	6.67E-003	sign
WE: Weak Enhancer	2304	16183	1647	17140	2.19E-031	1.00E-002	sign
I: Insulator	1038	17449	641	18146	4.81E-025	1.33E-002	sign
WT: Weak Transcribed	3587	14900	2952	15835	4.13E-021	1.67E-002	sign
TT: Transcriptional Transition	326	18161	213	18574	2.05E-007	2.00E-002	sign
SE: Strong Enhancer	1303	17184	1170	17617	7.84E-004	2.33E-002	sign
RP: Repetitive/Copy number variation	43	18444	19	18768	1.27E-003	2.67E-002	sign
RP: Repetitive/Copy number variation	92	18395	59	18728	3.30E-003	3.00E-002	sign
AP: Active Promoter	2521	15966	10169	8618	1.00E+000	3.33E-002	non-sign
WP: Weak Promoter	2108	16379	7024	11763	1.00E+000	3.67E-002	non-sign
IP: Inactive Promoter	889	17598	2218	16569	1.00E+000	4.00E-002	non-sign
SE: Strong Enhancer	1693	16794	4150	14637	1.00E+000	4.33E-002	non-sign
WE: Weak Enhancer	2530	15957	5944	12843	1.00E+000	4.67E-002	non-sign
PR: Polycomb Repressed	2695	15792	3475	15312	1.00E+000	5.00E-002	non-sign

NHLF	a	b	c	d	pvalue_Coding_over_NonCoding	Benjamini–Hochberg FDR correction (0.05)	FDR significance
AP: Active Promoter	10449	8338	2558	15929	0.00E+000	3.33E-003	sign
WP: Weak Promoter	8745	10042	2435	16052	0.00E+000	6.67E-003	sign
WE: Weak Enhancer	5779	13008	2333	16154	0.00E+000	1.00E-002	sign
SE: Strong Enhancer	2581	16206	1058	17429	3.12E-154	1.33E-002	sign
IP: Inactive Promoter	1708	17079	645	17842	1.68E-113	1.67E-002	sign
PR: Polycomb Repressed	3777	15010	2929	15558	4.72E-027	2.00E-002	sign
SE: Strong Enhancer	1256	17531	1306	17181	9.29E-001	2.33E-002	non-sign
RP: Repetitive/Copy number variation	55	18732	103	18384	1.00E+000	2.67E-002	non-sign
TT: Transcriptional Transition	238	18549	334	18153	1.00E+000	3.00E-002	non-sign
RP: Repetitive/Copy number variation	41	18746	95	18392	1.00E+000	3.33E-002	non-sign
WE: Weak Enhancer	1585	17202	2091	16396	1.00E+000	3.67E-002	non-sign
I: Insulator	1235	17552	1550	16937	1.00E+000	4.00E-002	non-sign
TE: Transcriptional Elongation	388	18399	1266	17221	1.00E+000	4.33E-002	non-sign
WT: Weak Transcribed	2776	16011	3597	14890	1.00E+000	4.67E-002	non-sign
HC: Heterochromatin low signal	6524	12263	10412	8075	1.00E+000	5.00E-002	non-sign

NHLF	a	b	c	d	pvalue_NonCoding_over_Coding	Benjamini–Hochberg FDR correction (0.05)	FDR significance
HC: Heterochromatin low signal	10412	8075	6524	12263	0.00E+000	3.33E-003	sign
TE: Transcriptional Elongation	1266	17221	388	18399	9.78E-117	6.67E-003	sign
WT: Weak Transcribed	3597	14890	2776	16011	1.79E-033	1.00E-002	sign
WE: Weak Enhancer	2091	16396	1585	17202	7.10E-021	1.33E-002	sign
I: Insulator	1550	16937	1235	17552	1.65E-011	1.67E-002	sign
RP: Repetitive/Copy number variation	95	18392	41	18746	1.28E-006	2.00E-002	sign
TT: Transcriptional Transition	334	18153	238	18549	1.30E-005	2.33E-002	sign
RP: Repetitive/Copy number variation	103	18384	55	18732	5.35E-005	2.67E-002	sign
SE: Strong Enhancer	1306	17181	1256	17531	7.70E-002	3.00E-002	non-sign
AP: Active Promoter	2558	15929	10449	8338	1.00E+000	3.33E-002	non-sign
WP: Weak Promoter	2435	16052	8745	10042	1.00E+000	3.67E-002	non-sign
IP: Inactive Promoter	645	17842	1708	17079	1.00E+000	4.00E-002	non-sign
SE: Strong Enhancer	1058	17429	2581	16206	1.00E+000	4.33E-002	non-sign
WE: Weak Enhancer	2333	16154	5779	13008	1.00E+000	4.67E-002	non-sign
PR: Polycomb Repressed	2929	15558	3777	15010	1.00E+000	5.00E-002	non-sign

Table S3. P-values of overrepresentation for chromatin states, CpG islands, repetitive elements and palindromes for complete promoter set (CPS) and repeat-filtered promoter set (REFPS).

**Contingency table (protein-coding genes promoters vs lncRNA promoters)**	
a:Total protein-coding genes promoters having this mark	b:Total protein-coding genes promoters NOT having this mark
c:Total lncRNA promoters having this mark	d: Total lncRNA promoters NOT having this mark

**Contingency table (lncRNA promoters vs protein-coding genes promoters)**	
a:Total lncRNA promoters having this mark	b: Total lncRNA promoters NOT having this mark
c:Total protein-coding genes promoters having this mark	d:Total protein-coding genes promoters NOT having this mark

Repeat-filtered promoter set (REFPS)

Protein-coding gene promoters vs lncRNA promoters

**Repeat Elements**	**a**	**b**	**c**	**d**	**pvalue_Coding_over_NonCoding**		
	6605	1325	9650	957	1.00E+00		
**CGI**	**a**	**b**	**c**	**d**	**pvalue_Coding_over_NonCoding**		
	4692	3238	1841	8766	0.00E+00		
**Palindromes**	**a**	**b**	**c**	**d**	**pvalue_Coding_over_NonCoding**		
	7930	0	10607	0	1.00E+00		
**GM12878**	**a**	**b**	**c**	**d**	**pvalue_Coding_over_NonCoding**	**Benjamini–Hochberg FDR correction (0.05)**	**FDR significance**
AP: Active Promoter	3967	3963	1016	9591	0.00E+000	3.33E-003	sign
WP: Weak Promoter	2929	5001	1022	9585	0.00E+000	6.67E-003	sign
WE: Weak Enhancer	2069	5861	1108	9499	4.16E-171	1.00E-002	sign
SE: Strong Enhancer	993	6937	546	10061	6.44E-072	1.33E-002	sign
IP: Inactive Promoter	396	7534	217	10390	1.73E-028	1.67E-002	sign
PR: Polycomb Repressed	939	6991	950	9657	9.41E-011	2.00E-002	sign
SE: Strong Enhancer	366	7564	431	10176	3.65E-002	2.33E-002	non-sign
WE: Weak Enhancer	845	7085	1198	9409	9.19E-001	2.67E-002	non-sign
RP: Repetitive/Copy number variation	15	7915	42	10565	9.97E-001	3.00E-002	non-sign
WT: Weak Transcribed	1221	6709	1854	8753	1.00E+000	3.33E-002	non-sign
TT: Transcriptional Transition	93	7837	198	10409	1.00E+000	3.67E-002	non-sign
RP: Repetitive/Copy number variation	25	7905	78	10529	1.00E+000	4.00E-002	non-sign
I: Insulator	246	7684	529	10078	1.00E+000	4.33E-002	non-sign
TE: Transcriptional Elongation	169	7761	823	9784	1.00E+000	4.67E-002	non-sign
HC: Heterochromatin low signal	3287	4643	6845	3762	1.00E+000	5.00E-002	non-sign

LncRNA promoters vs protein-coding gene promoters

**Repeat Elements**	**a**	**b**	**c**	**d**	**pvalue_NonCoding_over_Coding**		
	9650	957	6605	1325	1.81E-55		
**CGI**	**a**	**b**	**c**	**d**	**pvalue_NonCoding_over_Coding**		
	1841	8766	4692	3238	1.00E+00		
**Palindromes**	**a**	**b**	**c**	**d**	**pvalue_NonCoding_over_Coding**		
	10607	0	7930	0	1.00E+00		
**GM12878**	**a**	**b**	**c**	**d**	**pvalue_NonCoding_over_Coding**	**Benjamini–Hochberg FDR correction (0.05)**	**FDR significance**
HC: Heterochromatin low signal	6845	3762	3287	4643	1.90E-215	3.33E-003	sign
TE: Transcriptional Elongation	823	9784	169	7761	1.50E-070	6.67E-003	sign
I: Insulator	529	10078	246	7684	7.82E-011	1.00E-002	sign
RP: Repetitive/Copy number variation	78	10529	25	7905	6.79E-005	1.33E-002	sign
WT: Weak Transcribed	1854	8753	1221	6709	8.52E-005	1.67E-002	sign
TT: Transcriptional Transition	198	10409	93	7837	8.80E-005	2.00E-002	sign
RP: Repetitive/Copy number variation	42	10565	15	7915	7.51E-003	2.33E-002	sign
WE: Weak Enhancer	1198	9409	845	7085	8.84E-002	2.67E-002	non-sign
SE: Strong Enhancer	431	10176	366	7564	9.69E-001	3.00E-002	non-sign
AP: Active Promoter	1016	9591	3967	3963	1.00E+000	3.33E-002	non-sign
WP: Weak Promoter	1022	9585	2929	5001	1.00E+000	3.67E-002	non-sign
IP: Inactive Promoter	217	10390	396	7534	1.00E+000	4.00E-002	non-sign
SE: Strong Enhancer	546	10061	993	6937	1.00E+000	4.33E-002	non-sign
WE: Weak Enhancer	1108	9499	2069	5861	1.00E+000	4.67E-002	non-sign
PR: Polycomb Repressed	950	9657	939	6991	1.00E+000	5.00E-002	non-sign

Table S3. P-values of overrepresentation for chromatin states, CpG islands, repetitive elements and palindromes for complete promoter set (CPS) and repeat-filtered promoter set (REFPS).

H1-hESC	a	b	c	d	pvalue_Coding_over_NonCoding	Benjamini–Hochberg FDR correction (0.05)	FDR significance
AP: Active Promoter	3570	4360	849	9758	0.00E+000	3.33E-003	sign
WP: Weak Promoter	4125	3805	1195	9412	0.00E+000	6.67E-003	sign
WE: Weak Enhancer	3091	4839	1580	9027	2.95E-305	1.00E-002	sign
IP: Inactive Promoter	977	6953	575	10032	1.17E-062	1.33E-002	sign
SE: Strong Enhancer	347	7583	188	10419	1.52E-025	1.67E-002	sign
PR: Polycomb Repressed	774	7156	683	9924	8.75E-017	2.00E-002	sign
SE: Strong Enhancer	203	7727	242	10365	1.20E-001	2.33E-002	non-sign
TT: Transcriptional Transition	128	7802	234	10373	9.98E-001	2.67E-002	non-sign
RP: Repetitive/Copy number variation	8	7922	33	10574	1.00E+000	3.00E-002	non-sign
RP: Repetitive/Copy number variation	18	7912	56	10551	1.00E+000	3.33E-002	non-sign
WE: Weak Enhancer	1101	6829	1694	8913	1.00E+000	3.67E-002	non-sign
I: Insulator	370	7560	853	9754	1.00E+000	4.00E-002	non-sign
TE: Transcriptional Elongation	88	7842	533	10074	1.00E+000	4.33E-002	non-sign
WT: Weak Transcribed	1750	6180	2953	7654	1.00E+000	4.67E-002	non-sign
HC: Heterochromatin low signal	3389	4541	6567	4040	1.00E+000	5.00E-002	non-sign

H1-hESC	a	b	c	d	pvalue_NonCoding_over_Coding	Benjamini–Hochberg FDR correction (0.05)	FDR significance
HC: Heterochromatin low signal	6567	4040	3389	4541	1.15E-148	3.33E-003	sign
TE: Transcriptional Elongation	533	10074	88	7842	3.84E-055	6.67E-003	sign
I: Insulator	853	9754	370	7560	7.92E-021	1.00E-002	sign
WT: Weak Transcribed	2953	7654	1750	6180	1.65E-019	1.33E-002	sign
WE: Weak Enhancer	1694	8913	1101	6829	4.45E-005	1.67E-002	sign
RP: Repetitive/Copy number variation	56	10551	18	7912	7.28E-004	2.00E-002	sign
RP: Repetitive/Copy number variation	33	10574	8	7922	1.51E-003	2.33E-002	sign
TT: Transcriptional Transition	234	10373	128	7802	2.18E-003	2.67E-002	sign
SE: Strong Enhancer	242	10365	203	7727	8.98E-001	3.00E-002	non-sign
AP: Active Promoter	849	9758	3570	4360	1.00E+000	3.33E-002	non-sign
WP: Weak Promoter	1195	9412	4125	3805	1.00E+000	3.67E-002	non-sign
IP: Inactive Promoter	575	10032	977	6953	1.00E+000	4.00E-002	non-sign
SE: Strong Enhancer	188	10419	347	7583	1.00E+000	4.33E-002	non-sign
WE: Weak Enhancer	1580	9027	3091	4839	1.00E+000	4.67E-002	non-sign
PR: Polycomb Repressed	683	9924	774	7156	1.00E+000	5.00E-002	non-sign

HMEC	a	b	c	d	pvalue_Coding_over_NonCoding	Benjamini–Hochberg FDR correction (0.05)	FDR significance
AP: Active Promoter	3863	4067	901	9706	0.00E+000	3.33E-003	sign
WP: Weak Promoter	2967	4963	939	9668	0.00E+000	6.67E-003	sign
WE: Weak Enhancer	2524	5406	1338	9269	5.98E-222	1.00E-002	sign
SE: Strong Enhancer	1788	6142	767	9840	1.08E-196	1.33E-002	sign
IP: Inactive Promoter	344	7586	180	10427	9.50E-027	1.67E-002	sign
PR: Polycomb Repressed	615	7315	661	9946	3.04E-005	2.00E-002	sign
SE: Strong Enhancer	727	7203	912	9695	9.26E-002	2.33E-002	non-sign
RP: Repetitive/Copy number variation	13	7917	38	10569	9.97E-001	2.67E-002	non-sign
RP: Repetitive/Copy number variation	7	7923	27	10580	9.98E-001	3.00E-002	non-sign
TT: Transcriptional Transition	67	7863	155	10452	1.00E+000	3.33E-002	non-sign
WE: Weak Enhancer	1166	6764	1788	8819	1.00E+000	3.67E-002	non-sign
I: Insulator	201	7729	425	10182	1.00E+000	4.00E-002	non-sign
TE: Transcriptional Elongation	107	7823	680	9927	1.00E+000	4.33E-002	non-sign
WT: Weak Transcribed	1577	6353	2462	8145	1.00E+000	4.67E-002	non-sign
HC: Heterochromatin low signal	3500	4430	6737	3870	1.00E+000	5.00E-002	non-sign

HMEC	a	b	c	d	pvalue_NonCoding_over_Coding	Benjamini–Hochberg FDR correction (0.05)	FDR significance
HC: Heterochromatin low signal	6737	3870	3500	4430	1.82E-152	3.33E-003	sign
TE: Transcriptional Elongation	680	9927	107	7823	4.49E-073	6.67E-003	sign
I: Insulator	425	10182	201	7729	1.66E-008	1.00E-002	sign
WT: Weak Transcribed	2462	8145	1577	6353	2.93E-008	1.33E-002	sign
WE: Weak Enhancer	1788	8819	1166	6764	3.85E-005	1.67E-002	sign
TT: Transcriptional Transition	155	10452	67	7863	6.89E-005	2.00E-002	sign
RP: Repetitive/Copy number variation	27	10580	7	7923	5.79E-003	2.33E-002	sign
RP: Repetitive/Copy number variation	38	10569	13	7917	7.98E-003	2.67E-002	sign
SE: Strong Enhancer	912	9695	727	7203	9.16E-001	3.00E-002	non-sign
PR: Polycomb Repressed	661	9946	615	7315	1.00E+000	3.33E-002	non-sign
AP: Active Promoter	901	9706	3863	4067	1.00E+000	3.67E-002	non-sign
WP: Weak Promoter	939	9668	2967	4963	1.00E+000	4.00E-002	non-sign
IP: Inactive Promoter	180	10427	344	7586	1.00E+000	4.33E-002	non-sign
SE: Strong Enhancer	767	9840	1788	6142	1.00E+000	4.67E-002	non-sign
WE: Weak Enhancer	1338	9269	2524	5406	1.00E+000	5.00E-002	non-sign

Table S3. P-values of overrepresentation for chromatin states, CpG islands, repetitive elements and palindromes for complete promoter set (CPS) and repeat-filtered promoter set (REFPS).

HSMM	a	b	c	d	pvalue_Coding_over_NonCoding	Benjamini–Hochberg FDR correction (0.05)	FDR significance
AP: Active Promoter	3911	4019	967	9640	0.00E+000	3.33E-003	sign
WP: Weak Promoter	3706	4224	1136	9471	0.00E+000	6.67E-003	sign
WE: Weak Enhancer	2233	5697	1377	9230	6.73E-146	1.00E-002	sign
SE: Strong Enhancer	998	6932	700	9907	5.02E-044	1.33E-002	sign
IP: Inactive Promoter	376	7554	206	10401	4.21E-027	1.67E-002	sign
PR: Polycomb Repressed	887	7043	1046	9561	1.94E-003	2.00E-002	sign
RP: Repetitive/Copy number variation	54	7876	71	10536	4.96E-001	2.33E-002	non-sign
RP: Repetitive/Copy number variation	22	7908	37	10570	8.38E-001	2.67E-002	non-sign
TT: Transcriptional Transition	185	7745	345	10262	1.00E+000	3.00E-002	non-sign
WT: Weak Transcribed	1670	6260	2486	8121	1.00E+000	3.33E-002	non-sign
SE: Strong Enhancer	397	7533	799	9808	1.00E+000	3.67E-002	non-sign
WE: Weak Enhancer	677	7253	1381	9226	1.00E+000	4.00E-002	non-sign
I: Insulator	286	7644	541	10066	1.00E+000	4.33E-002	non-sign
TE: Transcriptional Elongation	257	7673	1057	9550	1.00E+000	4.67E-002	non-sign
HC: Heterochromatin low signal	3416	4514	6102	4505	1.00E+000	5.00E-002	non-sign

HSMM	a	b	c	d	pvalue_NonCoding_over_Coding	Benjamini–Hochberg FDR correction (0.05)	FDR significance
HC: Heterochromatin low signal	6102	4505	3416	4514	6.66E-085	3.33E-003	sign
TE: Transcriptional Elongation	1057	9550	257	7673	6.74E-076	6.67E-003	sign
WE: Weak Enhancer	1381	9226	677	7253	1.50E-022	1.00E-002	sign
SE: Strong Enhancer	799	9808	397	7533	1.49E-012	1.33E-002	sign
I: Insulator	541	10066	286	7644	5.18E-007	1.67E-002	sign
WT: Weak Transcribed	2486	8121	1670	6260	6.40E-005	2.00E-002	sign
TT: Transcriptional Transition	345	10262	185	7745	1.05E-004	2.33E-002	sign
RP: Repetitive/Copy number variation	37	10570	22	7908	2.36E-001	2.67E-002	non-sign
RP: Repetitive/Copy number variation	71	10536	54	7876	5.75E-001	3.00E-002	non-sign
PR: Polycomb Repressed	1046	9561	887	7043	9.98E-001	3.33E-002	non-sign
AP: Active Promoter	967	9640	3911	4019	1.00E+000	3.67E-002	non-sign
WP: Weak Promoter	1136	9471	3706	4224	1.00E+000	4.00E-002	non-sign
IP: Inactive Promoter	206	10401	376	7554	1.00E+000	4.33E-002	non-sign
SE: Strong Enhancer	700	9907	998	6932	1.00E+000	4.67E-002	non-sign
WE: Weak Enhancer	1377	9230	2233	5697	1.00E+000	5.00E-002	non-sign

HUVEC	a	b	c	d	pvalue_Coding_over_NonCoding	Benjamini–Hochberg FDR correction (0.05)	FDR significance
AP: Active Promoter	3766	4164	858	9749	0.00E+000	3.33E-003	sign
WP: Weak Promoter	2967	4963	878	9729	0.00E+000	6.67E-003	sign
SE: Strong Enhancer	2340	5590	977	9630	4.21E-279	1.00E-002	sign
WE: Weak Enhancer	2126	5804	988	9619	1.35E-217	1.33E-002	sign
IP: Inactive Promoter	505	7425	299	10308	1.36E-031	1.67E-002	sign
PR: Polycomb Repressed	1372	6558	1565	9042	1.53E-006	2.00E-002	sign
SE: Strong Enhancer	589	7341	751	9856	1.91E-001	2.33E-002	non-sign
WE: Weak Enhancer	848	7082	1196	9411	8.99E-001	2.67E-002	non-sign
RP: Repetitive/Copy number variation	31	7899	55	10552	9.16E-001	3.00E-002	non-sign
RP: Repetitive/Copy number variation	27	7903	55	10552	9.74E-001	3.33E-002	non-sign
I: Insulator	297	7633	522	10085	1.00E+000	3.67E-002	non-sign
TT: Transcriptional Transition	67	7863	180	10427	1.00E+000	4.00E-002	non-sign
TE: Transcriptional Elongation	120	7810	632	9975	1.00E+000	4.33E-002	non-sign
WT: Weak Transcribed	1200	6730	1929	8678	1.00E+000	4.67E-002	non-sign
HC: Heterochromatin low signal	3438	4492	6643	3964	1.00E+000	5.00E-002	non-sign

HUVEC	a	b	c	d	pvalue_NonCoding_over_Coding	Benjamini–Hochberg FDR correction (0.05)	FDR significance
HC: Heterochromatin low signal	6643	3964	3438	4492	1.93E-150	3.33E-003	sign
TE: Transcriptional Elongation	632	9975	120	7810	3.84E-058	6.67E-003	sign
WT: Weak Transcribed	1929	8678	1200	6730	1.97E-008	1.00E-002	sign
TT: Transcriptional Transition	180	10427	67	7863	2.11E-007	1.33E-002	sign
I: Insulator	522	10085	297	7633	6.01E-005	1.67E-002	sign
RP: Repetitive/Copy number variation	55	10552	27	7903	4.37E-002	2.00E-002	non-sign
WE: Weak Enhancer	1196	9411	848	7082	1.10E-001	2.33E-002	non-sign
RP: Repetitive/Copy number variation	55	10552	31	7899	1.23E-001	2.67E-002	non-sign
SE: Strong Enhancer	751	9856	589	7341	8.24E-001	3.00E-002	non-sign
PR: Polycomb Repressed	1565	9042	1372	6558	1.00E+000	3.33E-002	non-sign
AP: Active Promoter	858	9749	3766	4164	1.00E+000	3.67E-002	non-sign
WP: Weak Promoter	878	9729	2967	4963	1.00E+000	4.00E-002	non-sign
IP: Inactive Promoter	299	10308	505	7425	1.00E+000	4.33E-002	non-sign
SE: Strong Enhancer	977	9630	2340	5590	1.00E+000	4.67E-002	non-sign
WE: Weak Enhancer	988	9619	2126	5804	1.00E+000	5.00E-002	non-sign

Table S3. P-values of overrepresentation for chromatin states, CpG islands, repetitive elements and palindromes for complete promoter set (CPS) and repeat-filtered promoter set (REFPS).

NHEK	a	b	c	d	pvalue_Coding_over_NonCoding	Benjamini–Hochberg FDR correction (0.05)	FDR significance
AP: Active Promoter	4039	3891	1024	9583	0.00E+000	3.33E-003	sign
WP: Weak Promoter	2614	5316	893	9714	0.00E+000	6.67E-003	sign
SE: Strong Enhancer	1838	6092	906	9701	7.08E-169	1.00E-002	sign
WE: Weak Enhancer	2203	5727	1247	9360	3.14E-168	1.33E-002	sign
IP: Inactive Promoter	478	7452	329	10278	5.86E-022	1.67E-002	sign
PR: Polycomb Repressed	1145	6785	1462	9145	1.06E-001	2.00E-002	non-sign
SE: Strong Enhancer	576	7354	799	9808	7.64E-001	2.33E-002	non-sign
RP: Repetitive/Copy number variation	8	7922	17	10590	9.04E-001	2.67E-002	non-sign
RP: Repetitive/Copy number variation	19	7911	40	10567	9.64E-001	3.00E-002	non-sign
TT: Transcriptional Transition	124	7806	218	10389	9.94E-001	3.33E-002	non-sign
WT: Weak Transcribed	1411	6519	2159	8448	1.00E+000	3.67E-002	non-sign
WE: Weak Enhancer	814	7116	1414	9193	1.00E+000	4.00E-002	non-sign
I: Insulator	293	7637	639	9968	1.00E+000	4.33E-002	non-sign
TE: Transcriptional Elongation	187	7743	813	9794	1.00E+000	4.67E-002	non-sign
HC: Heterochromatin low signal	3192	4738	6325	4282	1.00E+000	5.00E-002	non-sign

NHEK	a	b	c	d	pvalue_NonCoding_over_Coding	Benjamini–Hochberg FDR correction (0.05)	FDR significance
HC: Heterochromatin low signal	6325	4282	3192	4738	2.48E-151	3.33E-003	sign
TE: Transcriptional Elongation	813	9794	187	7743	8.65E-062	6.67E-003	sign
I: Insulator	639	9968	293	7637	2.01E-013	1.00E-002	sign
WE: Weak Enhancer	1414	9193	814	7116	9.51E-011	1.33E-002	sign
WT: Weak Transcribed	2159	8448	1411	6519	6.27E-006	1.67E-002	sign
TT: Transcriptional Transition	218	10389	124	7806	7.74E-003	2.00E-002	sign
RP: Repetitive/Copy number variation	40	10567	19	7911	6.37E-002	2.33E-002	non-sign
RP: Repetitive/Copy number variation	17	10590	8	7922	1.88E-001	2.67E-002	non-sign
SE: Strong Enhancer	799	9808	576	7354	2.54E-001	3.00E-002	non-sign
PR: Polycomb Repressed	1462	9145	1145	6785	9.02E-001	3.33E-002	non-sign
AP: Active Promoter	1024	9583	4039	3891	1.00E+000	3.67E-002	non-sign
WP: Weak Promoter	893	9714	2614	5316	1.00E+000	4.00E-002	non-sign
IP: Inactive Promoter	329	10278	478	7452	1.00E+000	4.33E-002	non-sign
SE: Strong Enhancer	906	9701	1838	6092	1.00E+000	4.67E-002	non-sign
WE: Weak Enhancer	1247	9360	2203	5727	1.00E+000	5.00E-002	non-sign

NHLF	a	b	c	d	pvalue_Coding_over_NonCoding	Benjamini–Hochberg FDR correction (0.05)	FDR significance
AP: Active Promoter	4097	3833	1014	9593	0.00E+000	3.33E-003	sign
WP: Weak Promoter	3330	4600	1010	9597	0.00E+000	6.67E-003	sign
WE: Weak Enhancer	2082	5848	1119	9488	2.15E-171	1.00E-002	sign
SE: Strong Enhancer	1096	6834	575	10032	2.67E-086	1.33E-002	sign
IP: Inactive Promoter	411	7519	218	10389	4.02E-031	1.67E-002	sign
PR: Polycomb Repressed	1323	6607	1584	9023	6.51E-004	2.00E-002	sign
SE: Strong Enhancer	583	7347	794	9813	6.45E-001	2.33E-002	non-sign
TT: Transcriptional Transition	132	7798	227	10380	9.92E-001	2.67E-002	non-sign
RP: Repetitive/Copy number variation	17	7913	44	10563	9.94E-001	3.00E-002	non-sign
RP: Repetitive/Copy number variation	13	7917	43	10564	9.99E-001	3.33E-002	non-sign
WE: Weak Enhancer	739	7191	1258	9349	1.00E+000	3.67E-002	non-sign
I: Insulator	539	7391	938	9669	1.00E+000	4.00E-002	non-sign
TE: Transcriptional Elongation	236	7694	886	9721	1.00E+000	4.33E-002	non-sign
WT: Weak Transcribed	1352	6578	2210	8397	1.00E+000	4.67E-002	non-sign
HC: Heterochromatin low signal	3199	4731	6147	4460	1.00E+000	5.00E-002	non-sign

NHLF	a	b	c	d	pvalue_NonCoding_over_Coding	Benjamini–Hochberg FDR correction (0.05)	FDR significance
HC: Heterochromatin low signal	6147	4460	3199	4731	2.99E-125	3.33E-003	sign
TE: Transcriptional Elongation	886	9721	236	7694	2.83E-056	6.67E-003	sign
WT: Weak Transcribed	2210	8397	1352	6578	4.54E-011	1.00E-002	sign
WE: Weak Enhancer	1258	9349	739	7191	1.61E-008	1.33E-002	sign
I: Insulator	938	9669	539	7391	1.73E-007	1.67E-002	sign
RP: Repetitive/Copy number variation	43	10564	13	7917	1.80E-003	2.00E-002	sign
TT: Transcriptional Transition	227	10380	132	7798	1.12E-002	2.33E-002	sign
RP: Repetitive/Copy number variation	44	10563	17	7913	1.17E-002	2.67E-002	sign
SE: Strong Enhancer	794	9813	583	7347	3.77E-001	3.00E-002	non-sign
PR: Polycomb Repressed	1584	9023	1323	6607	9.99E-001	3.33E-002	non-sign
AP: Active Promoter	1014	9593	4097	3833	1.00E+000	3.67E-002	non-sign
WP: Weak Promoter	1010	9597	3330	4600	1.00E+000	4.00E-002	non-sign
IP: Inactive Promoter	218	10389	411	7519	1.00E+000	4.33E-002	non-sign
SE: Strong Enhancer	575	10032	1096	6834	1.00E+000	4.67E-002	non-sign
WE: Weak Enhancer	1119	9488	2082	5848	1.00E+000	5.00E-002	non-sign
