# Supplementary material for: Promoter Analysis Reveals Globally Differential Regulation of Human Long Non-Coding RNA and Protein-Coding Genes
Source: PLoS One. 2014 Oct 2;9(10):e109443. doi: 10.1371/journal.pone.0109443 (PMC4183604; doi:10.1371/journal.pone.0109443)
Supplement: Table S4 — a. Summary of the results for separation of promoters of protein-coding and lncRNA genes using different combinations of features for the complete promoter set (CPS) and repeat-filtered promoter set (REFPS). For REFPS, we used all types of repeats except “simple repeats”, “low complexity regions” and “satellite repeats”. k-mer: mono-, di-,tri-nucleotide frequencies, CS: chromatin states, WC: word commonality, TFBS: transcription factor binding sites, CGI: CpG Islands, SKEW: A/T and C/G skews, PALIN: palindromes, RE: repetitive elements, COMBINE: combination of all types of features. b. Summary of the cross validation (CV) results for separation of promoters of protein-coding from lncRNA genes using all features (COMBINE) for completer promoter set (CPS) and repeat-filtered promoter set (REFPS). (PDF) [file pone.0109443.s010.pdf]

Table S4 a. Summary of the results for separation of promoters of protein-coding and lncRNA genes using different combinations of features for the complete promoter set (CPS) and repeat-filtered promoter set (REFPS). For REFPS, we used all types of repeats except "simple repeats", "low complexity regions" and "satellite repeats". k-mer: mono-, di-,tri-nucleotide frequencies, CS: chromatin states, WC: word commonality, TFBS: transcription factor binding sites, CGI: CpG Islands, SKEW: A/T and C/G skews, , PALIN: palindromes, RE: repetitive elements, COMBINE: combination of all types of features.

Features	#Features	CPS			REFPS		
		Sensitivity (%)	Specificity (%)	Accuracy (%)	Sensitivity (%)	Specificity (%)	Accuracy (%)
K-mer	168	82.750	77.208	79.957	87.282	68.436	79.220
CS	105	78.428	73.013	75.699	85.896	61.576	75.492
WC	39	80.862	72.476	76.635	86.000	61.980	75.724
TFBS	426	70.790	73.476	72.144	79.457	59.634	70.977
CGI	39	84.513	64.278	74.315	87.537	61.639	76.458
SKEW	78	64.261	70.948	67.632	81.060	50.088	67.810
PALIN	39	64.132	55.554	59.808	81.314	29.029	58.947
RE	39	60.891	76.867	68.943	68.040	73.317	70.297
COMBINE	933	82.788	80.609	81.690	87.414	73.190	81.329
CG+CGI	144	82.582	72.508	77.504	87.537	61.639	76.458
All except K-mer,WC	726	81.219	79.438	80.321	86.565	70.971	79.894
All except K-mer,WC, PALIN	687	81.371	79.555	80.456	86.613	71.665	80.218

Table S4 b. Summary of the cross validation (CV) results for separation of promoters of protein-coding from lncRNA genes using all features (COMBINE) for completer promoter set (CPS) and repeat-filtered promoter set (REFPS).

Fold	CPS			REFPS		
	Sensitivity(%)	Specificity (%)	Accuracy (%)	Sensitivity %)	Specificity (%)	Accuracy (%)
1	82.33333333	78.92004154	80.56897477	83.80566802	75.46296296	79.91360691
2	85.25104603	79.07488987	82.24248927	86.6419295	72.16494845	80.58252427
3	85.14099783	79.29936306	82.1888412	89.01960784	72.1822542	81.44552319
4	83.57705287	81.53846154	82.51072961	88.11700183	75.00	82.74002157
5	84.04588113	79.66850829	81.92060086	88.47583643	76.3496144	83.38727077
6	82.32758621	80.98290598	81.65236052	89.23076923	70.51597052	81.01402373
7	86.00867679	82.59023355	84.28111588	85.76709797	75.12953368	81.33764833
8	79.28870293	79.40528634	79.34549356	87.31060606	72.18045113	80.798274
9	81.07810781	80.94240838	81.00858369	82.77886497	74.03846154	78.85652643
10	81.10859729	80.71428571	80.90128755	87.06739526	72.22222222	81.01402373
11	83.31562168	82.55687974	82.93991416	88.34951456	74.27184466	82.09277238
12	84.69827586	80.34188034	82.51072961	87.76699029	76.45631068	82.74002157
13	82.1466525	81.04008667	81.59871245	85.38461538	72.48157248	79.71952535
14	82.37214363	81.05820106	81.70600858	87.99249531	72.08121827	81.22977346
15	81.7887931	79.91452991	80.84763948	87.05440901	73.0964467	81.1218986
16	80.96846847	80.82051282	80.89103596	87.36654804	72.60273973	81.55339806
17	82.70440252	81.18811881	81.96457327	88.09980806	70.93596059	80.58252427
18	81.4973262	81.35775862	81.42780462	90.94269871	71.50259067	82.84789644
19	83.03769401	80.95733611	81.96457327	88.53974122	72.20779221	81.74946004
20	82.93736501	79.72251868	81.32045089	88.16029144	72.67904509	81.8574514
Average	82.78133626	80.60471035	81.68959596	87.39359446	73.17809701	81.32920823
