# Supplementary material for: Promoter Analysis Reveals Globally Differential Regulation of Human Long Non-Coding RNA and Protein-Coding Genes
Source: PLoS One. 2014 Oct 2;9(10):e109443. doi: 10.1371/journal.pone.0109443 (PMC4183604; doi:10.1371/journal.pone.0109443)
Supplement: Table S5 — Results from execution of the computational model from promoters considering only upstream ([−1000, 0]) of TSS, as well as from promoters considering only upstream ([−1000, 0]) of TSS having no overlap with CGI. (PDF) [file pone.0109443.s011.pdf]

Supplementary Tables S5: Results from execution of the computational model from promoters considering only upstream ([-1000,0]) of TSS, as well as from promoters considering only upstream ([-1000,0]) of TSS having no overlap with CGI	
**Title**	**Description**
**Result_Upstream**	Result of computational model on different fold for promoter([-1000,0] bp) of gene having no overlap with CDS
**Result_Upstream_CGI**	promoter([-1000,0] bp) of gene having no overlap with CDS and CGI

Result_Upstream:Results for the computational model from promoters considering only upstream ([-1000,0]) of TSS.

Fold	Sensivity	Specificity	Accuracy
1	80.93681917	75.66137566	78.26087
2	79.31769723	71.59827214	75.482833
3	78.82352941	75.45748116	77.145923
4	78.92004154	75.91564928	77.467811
5	78.91891892	73.26943557	76.072961
6	80.63112078	73.43915344	76.984979
7	77.49490835	74.26303855	75.965665
8	78.81081081	74.65388711	76.716738
9	78.44827586	72.54273504	75.482833
10	80.33333333	74.58506224	77.360515
11	80.88552916	74.52025586	77.682403
12	80.04314995	73.63927428	76.824034
13	80.95238095	74.29760666	77.521459
14	80.42780749	72.33584499	76.39485
15	79.5404814	74.10526316	76.770386
16	81.22941822	73.52941176	77.294686
17	77.54880694	74.49521785	76.006441
18	80.72687225	73.82198953	77.187332
19	79.93158495	73.12373225	76.328502
20	79.80665951	69.42060086	74.610843
Average	79.68640731	73.73376437	76.678103

Result_Upstream_CGI:Results for computational model from promoters considering only upstream ([-1000,0]) of TSS having no overlap with CGI.

Run	Fold	Sensivity (%)	Specificity (%)	Accuracy (%)	Average Sensivity (%)	Average Specificity (%)	Average Accuracy (%)
1	1	68.40278	74.32836	71.58909			
	2	69.10828	72.58065	70.83333			
	3	63.49206	75.40453	69.39103			
	4	64.2623	76.17555	70.35256			
	5	65.75758	78.57143	71.79487			
	6	67.40506	76.94805	72.11538			
	7	65.04559	76.94915	70.67308			
	8	64.50512	77.03927	71.15385			
	9	64.0625	75.65789	69.71154			
	10	65.25974	76.58228	70.99359			
	11	59.35484	80.25478	69.87179			
	12	63.0363	79.43925	71.47436			
	13	69.23077	76.30769	72.91667			
	14	66.23377	80.6962	73.55769			
	15	60.96774	77.70701	69.39103			
	16	68.07818	77.91798	73.07692			
	17	61.44201	78.68852	69.87179			
	18	62.5387	73.0897	67.62821			
	19	63.52584	75.59322	69.23077			
	20	64.53674	74.51613	69.50241	64.81229	76.72238	70.7565
2	1	65.63467	77	71.10754			
	2	60.45752	80.81761	70.83333			
	3	71.5655	80.7074	76.12179			
	4	67.9868	80.06231	74.19872			
	5	69.25566	78.73016	74.03846			
	6	65.15152	77.89116	71.15385			
	7	65.2027	78.04878	71.95513			
	8	63.66667	78.7037	71.47436			
	9	67.75244	82.64984	75.32051			
	10	66.25387	73.75415	69.87179			
	11	61.8123	79.04762	70.51282			
	12	64.80263	79.0625	72.11538			
	13	63.24503	77.95031	70.83333			
	14	62.5387	78.73754	70.35256			
	15	62.05788	76.35783	69.23077			
	16	61	79.32099	70.51282			
	17	59.32722	77.10438	67.78846			
	18	66.66667	78.81944	72.27564			

Result_Upstream_CGI:Results for computational model from promoters considering only upstream ([-1000,0]) of TSS having no overlap with CGI.

	**19**	65.83851	78.47682	71.95513			
	**20**	64.0678	71.34146	67.89727	64.7142	78.2292	71.47748
**3**	**1**	66.0066	74.0625	70.14446			
	**2**	63.19218	76.65615	70.03205			
	**3**	67.61006	75.81699	71.63462			
	**4**	60.75949	77.27273	68.91026			
	**5**	63.02251	76.67732	69.87179			
	**6**	66.97819	77.22772	71.95513			
	**7**	59.50156	80.52805	69.71154			
	**8**	67.62821	76.28205	71.95513			
	**9**	67.19745	78.06452	72.59615			
	**10**	67.83439	74.51613	71.15385			
	**11**	62.73885	80.32258	71.47436			
	**12**	65.55184	73.84615	69.87179			
	**13**	65.26316	75.51622	70.83333			
	**14**	60.96774	80.57325	70.83333			
	**15**	67.30159	77.6699	72.4359			
	**16**	63.92405	74.02597	68.91026			
	**17**	71.47436	82.05128	76.76282			
	**18**	69.53642	77.95031	73.87821			
	**19**	67.15543	79.5053	72.75641			
	**20**	62.66234	81.90476	72.39165	65.31532	77.52349	71.40565
**4**	**1**	64.08046	76	69.34189			
	**2**	61.68831	76.26582	69.07051			
	**3**	63.93939	76.19048	69.71154			
	**4**	64	78.08642	71.3141			
	**5**	61.68224	81.84818	71.47436			
	**6**	64.07767	76.50794	70.35256			
	**7**	64.16382	78.24773	71.63462			
	**8**	62.57862	80.71895	71.47436			
	**9**	60.20761	74.92537	68.10897			
	**10**	68.16609	79.40299	74.19872			
	**11**	64.33121	83.22581	73.71795			
	**12**	57.2327	77.45098	67.14744			
	**13**	68.24324	77.7439	73.23718			
	**14**	65.03268	75.78616	70.51282			
	**15**	68.19572	75.08418	71.47436			
	**16**	62.77603	81.75896	72.11538			
	**17**	64.26332	76.06557	70.03205			
	**18**	67.83439	80	73.87821			
	**19**	62.12625	77.08978	69.87179			

Result_Upstream_CGI:Results for computational model from promoters considering only upstream ([-1000,0]) of TSS having no overlap with CGI.

	**20**	62.73292	78.73754	70.46549	63.86763	78.05684	70.95672
**5**	**1**	70.06369	78.31715	74.1573			
	**2**	64.15094	73.20261	68.58974			
	**3**	61.96721	78.68339	70.51282			
	**4**	67.84566	77.31629	72.59615			
	**5**	64.42308	78.20513	71.3141			
	**6**	63.90728	79.19255	71.79487			
	**7**	64.85623	78.77814	71.79487			
	**8**	63.39869	75.15723	69.39103			
	**9**	67.19243	75.89577	71.47436			
	**10**	62.5	76.92308	69.71154			
	**11**	64.59016	73.04075	68.91026			
	**12**	65.42056	74.91749	70.03205			
	**13**	61.88925	79.81073	70.99359			
	**14**	70.38217	78.70968	74.51923			
	**15**	65.74924	80.80808	72.91667			
	**16**	63.6646	75.49669	69.39103			
	**17**	64	75	69.71154			
	**18**	62.89308	76.14379	69.39103			
	**19**	62.0462	79.12773	70.83333			
	**20**	68.58974	82.31511	75.44141	64.97651	77.35207	71.17385
	**Average**				64.73719	77.5768	71.15404
