# Supplementary material for: Promoter Analysis Reveals Globally Differential Regulation of Human Long Non-Coding RNA and Protein-Coding Genes
Source: PLoS One. 2014 Oct 2;9(10):e109443. doi: 10.1371/journal.pone.0109443 (PMC4183604; doi:10.1371/journal.pone.0109443)
Supplement: Table S6 — P-values of overrepresentation for chromatin states for similarly expressed genes promoter in complete promoter set (CPS). (PDF) [file pone.0109443.s012.pdf]

Table S6. P-values of overrepresentation for chromatin states for similarly expressed genes promoter in complete promoter set (CPS).

Contingency table	
a:Total protein-coding genes promoters having this mark	b:Total protein-coding genes promoters NOT having this mark
c:Total lncRNA promoters having this mark	d: Total lncRNA promoters NOT having this mark

Contingency table	
a:Total lncRNA promoters having this mark	b: Total lncRNA promoters NOT having this mark
c:Total protein-coding genes promoters having this mark	d:Total protein-coding genes promoters NOT having this mark

	Complete promoter set (CPS)															
	Protein-coding gene promoters vs lncRNA promoters							LncRNA promoters vs protein-coding gene promoters								
**GM12878**	**a**	**b**	**c**	**d**	**pvalCoverNC**	**Benjamini–Hochberg FDR correction (0.05)**	**FDR significance**		**a**	**b**	**c**	**d**	**pvalNCoverC**	**Benjamini–Hochberg FDR correction (0.05)**	**FDR significance**
WP: Weak Promoter	3329	5052	1581	6800	1.06E-196	4.44E-002	sign	TE: Transcriptional Elongation	1086	7295	127	8254	1.84E-203	4.44E-002	sign
PR: Polycomb Repressed	1887	6494	555	7826	6.23E-196	3.89E-002	sign	TT: Transcriptional Transition	277	8104	68	8313	6.18E-32	3.89E-002	sign
IP: Inactive Promoter	1289	7092	267	8114	9.55E-176	3.33E-002	sign	WT: Weak Transcribed	2019	6362	1424	6957	2.65E-30	3.33E-002	sign
WE: Weak Enhancer	2712	5669	1378	7003	4.27E-129	2.78E-002	sign	SE: Strong Enhancer	504	7877	292	8089	6.49E-15	2.78E-002	sign
AP: Active Promoter	3278	5103	1863	6518	1.67E-125	2.22E-002	sign	HC: Heterochromatin low signal	3697	4684	3358	5023	6.16E-08	2.22E-002	sign
SE: Strong Enhancer	713	7668	771	7610	9.46E-01	1.67E-002	non-sign	WE: Weak Enhancer	1126	7255	956	7425	3.76E-05	1.67E-002	sign
RP: Repetitive/Copy number variation	55	8326	73	8308	9.54E-01	1.11E-002	non-sign	RP: Repetitive/Copy number variation	45	8336	18	8363	4.39E-04	1.11E-002	sign
I: Insulator	306	8075	384	7997	9.99E-01	5.56E-003	non-sign	I: Insulator	384	7997	306	8075	1.37E-03	5.56E-003	sign
RP: Repetitive/Copy number variation	18	8363	45	8336	1.00E+00	1.11E-002	non-sign	SE: Strong Enhancer	771	7610	713	7668	6.06E-02	1.11E-002	non-sign
WE: Weak Enhancer	956	7425	1126	7255	1.00E+00	1.67E-002	non-sign	RP: Repetitive/Copy number variation	73	8308	55	8326	6.56E-02	1.67E-002	non-sign
SE: Strong Enhancer	292	8089	504	7877	1.00E+00	2.22E-002	non-sign	AP: Active Promoter	1863	6518	3278	5103	1.00E+00	2.22E-002	non-sign
TT: Transcriptional Transition	68	8313	277	8104	1.00E+00	2.78E-002	non-sign	WP: Weak Promoter	1581	6800	3329	5052	1.00E+00	2.78E-002	non-sign
TE: Transcriptional Elongation	127	8254	1086	7295	1.00E+00	3.33E-002	non-sign	IP: Inactive Promoter	267	8114	1289	7092	1.00E+00	3.33E-002	non-sign
WT: Weak Transcribed	1424	6957	2019	6362	1.00E+00	3.89E-002	non-sign	WE: Weak Enhancer	1378	7003	2712	5669	1.00E+00	3.89E-002	non-sign
HC: Heterochromatin low signal	3358	5023	3697	4684	1.00E+00	3.89E-002	non-sign	PR: Polycomb Repressed	555	7826	1887	6494	1.00E+00	3.89E-002	non-sign

Table S6. P-values of overrepresentation for chromatin states for similarly expressed genes promoter in complete promoter set (CPS).

Contingency table	
a:Total protein-coding genes promoters having this mark	b:Total protein-coding genes promoters NOT having this mark
c:Total lncRNA promoters having this mark	d: Total lncRNA promoters NOT having this mark

Contingency table	
a:Total lncRNA promoters having this mark	b: Total lncRNA promoters NOT having this mark
c:Total protein-coding genes promoters having this mark	d:Total protein-coding genes promoters NOT having this mark

	Complete promoter set (CPS)														
	Protein-coding gene promoters vs lncRNA promoters							LncRNA promoters vs protein-coding gene promoters							
H1-hESC	a	b	c	d	pvalCoverNC	Benjamini–Hochberg FDR correction (0.05)	FDR significance		a	b	c	d	pvalNCoverC	Benjamini–Hochberg FDR correction (0.05)	FDR significance
IP: Inactive Promoter	2499	5548	754	7293	2.44E-268	4.44E-002	sign	TE: Transcriptional Elongation	700	7347	41	8006	2.99E-162	4.44E-002	sign
WP: Weak Promoter	3303	4744	2014	6033	2.13E-104	3.89E-002	sign	WT: Weak Transcribed	2752	5295	1675	6372	2.40E-81	3.89E-002	sign
PR: Polycomb Repressed	1515	6532	611	7436	4.00E-101	3.33E-002	sign	TT: Transcriptional Transition	331	7716	76	7971	3.16E-40	3.33E-002	sign
WE: Weak Enhancer	2842	5205	1869	6178	2.92E-64	2.78E-002	sign	SE: Strong Enhancer	237	7810	148	7899	2.58E-06	2.78E-002	sign
AP: Active Promoter	2144	5903	1546	6501	1.74E-29	2.22E-002	sign	I: Insulator	544	7503	425	7622	4.53E-05	2.22E-002	sign
WE: Weak Enhancer	1239	6808	1293	6754	8.83E-01	1.67E-002	non-sign	RP: Repetitive/Copy number variation	34	8013	9	8038	8.35E-05	1.67E-002	sign
RP: Repetitive/Copy number variation	20	8027	42	8005	9.98E-01	1.11E-002	non-sign	HC: Heterochromatin low signal	3350	4697	3142	4905	4.40E-04	1.11E-002	sign
SE: Strong Enhancer	185	7862	248	7799	9.99E-01	5.56E-003	non-sign	SE: Strong Enhancer	248	7799	185	7862	1.24E-03	5.56E-003	sign
HC: Heterochromatin low signal	3142	4905	3350	4697	1.00E+00	1.11E-002	non-sign	RP: Repetitive/Copy number variation	42	8005	20	8027	3.52E-03	1.11E-002	sign
I: Insulator	425	7622	544	7503	1.00E+00	1.67E-002	non-sign	WE: Weak Enhancer	1293	6754	1239	6808	1.26E-01	1.67E-002	non-sign
RP: Repetitive/Copy number variation	9	8038	34	8013	1.00E+00	2.22E-002	non-sign	AP: Active Promoter	1546	6501	2144	5903	1.00E+00	2.22E-002	non-sign
SE: Strong Enhancer	148	7899	237	7810	1.00E+00	2.78E-002	non-sign	WP: Weak Promoter	2014	6033	3303	4744	1.00E+00	2.78E-002	non-sign
TT: Transcriptional Transition	76	7971	331	7716	1.00E+00	3.33E-002	non-sign	IP: Inactive Promoter	754	7293	2499	5548	1.00E+00	3.33E-002	non-sign
TE: Transcriptional Elongation	41	8006	700	7347	1.00E+00	3.89E-002	non-sign	WE: Weak Enhancer	1869	6178	2842	5205	1.00E+00	3.89E-002	non-sign
WT: Weak Transcribed	1675	6372	2752	5295	1.00E+00	3.89E-002	non-sign	PR: Polycomb Repressed	611	7436	1515	6532	1.00E+00	3.89E-002	non-sign

Table S6. P-values of overrepresentation for chromatin states for similarly expressed genes promoter in complete promoter set (CPS).

Contingency table	
a:Total protein-coding genes promoters having this mark	b:Total protein-coding genes promoters NOT having this mark
c:Total lncRNA promoters having this mark	d: Total lncRNA promoters NOT having this mark

Contingency table	
a:Total lncRNA promoters having this mark	b: Total lncRNA promoters NOT having this mark
c:Total protein-coding genes promoters having this mark	d:Total protein-coding genes promoters NOT having this mark

	Complete promoter set (CPS)														
	Protein-coding gene promoters vs lncRNA promoters							LncRNA promoters vs protein-coding gene promoters							
HUVEC	a	b	c	d	pvalCoverNC	Benjamini–Hochberg FDR correction (0.05)	FDR significance		a	b	c	d	pvalNCoverC	Benjamini–Hochberg FDR correction (0.05)	FDR significance
WP: Weak Promoter	3040	4288	1323	6005	1.28E-215	4.44E-002	sign	TE: Transcriptional Elongation	833	6495	80	7248	4.83E-168	4.44E-002	sign
PR: Polycomb Repressed	2133	5195	757	6571	1.59E-185	3.89E-002	sign	WT: Weak Transcribed	2079	5249	1158	6170	4.52E-76	3.89E-002	sign
IP: Inactive Promoter	1342	5986	289	7039	8.14E-181	3.33E-002	sign	TT: Transcriptional Transition	241	7087	34	7294	1.51E-40	3.33E-002	sign
AP: Active Promoter	2788	4540	1613	5715	1.55E-100	2.78E-002	sign	SE: Strong Enhancer	776	6552	412	6916	9.06E-29	2.78E-002	sign
WE: Weak Enhancer	2299	5029	1226	6102	8.02E-97	2.22E-002	sign	WE: Weak Enhancer	1083	6245	820	6508	5.70E-11	2.22E-002	sign
SE: Strong Enhancer	1517	5811	1410	5918	1.43E-02	1.67E-002	sign	RP: Repetitive/Copy number variation	58	7270	26	7302	3.03E-04	1.67E-002	sign
HC: Heterochromatin low signal	2906	4422	2923	4405	6.19E-01	1.11E-002	non-sign	RP: Repetitive/Copy number variation	60	7268	30	7298	9.98E-04	1.11E-002	sign
I: Insulator	318	7010	346	6982	8.75E-01	5.56E-003	non-sign	I: Insulator	346	6982	318	7010	1.42E-01	5.56E-003	non-sign
RP: Repetitive/Copy number variation	30	7298	60	7268	1.00E+00	1.11E-002	non-sign	HC: Heterochromatin low signal	2923	4405	2906	4422	3.94E-01	1.11E-002	non-sign
RP: Repetitive/Copy number variation	26	7302	58	7270	1.00E+00	1.67E-002	non-sign	SE: Strong Enhancer	1410	5918	1517	5811	9.87E-01	1.67E-002	non-sign
SE: Strong Enhancer	412	6916	776	6552	1.00E+00	2.22E-002	non-sign	AP: Active Promoter	1613	5715	2788	4540	1.00E+00	2.22E-002	non-sign
WE: Weak Enhancer	820	6508	1083	6245	1.00E+00	2.78E-002	non-sign	WP: Weak Promoter	1323	6005	3040	4288	1.00E+00	2.78E-002	non-sign
TT: Transcriptional Transition	34	7294	241	7087	1.00E+00	3.33E-002	non-sign	IP: Inactive Promoter	289	7039	1342	5986	1.00E+00	3.33E-002	non-sign
TE: Transcriptional Elongation	80	7248	833	6495	1.00E+00	3.89E-002	non-sign	WE: Weak Enhancer	1226	6102	2299	5029	1.00E+00	3.89E-002	non-sign
WT: Weak Transcribed	1158	6170	2079	5249	1.00E+00	3.89E-002	non-sign	PR: Polycomb Repressed	757	6571	2133	5195	1.00E+00	3.89E-002	non-sign
