# Supplementary material for: Promoter Analysis Reveals Globally Differential Regulation of Human Long Non-Coding RNA and Protein-Coding Genes
Source: PLoS One. 2014 Oct 2;9(10):e109443. doi: 10.1371/journal.pone.0109443 (PMC4183604; doi:10.1371/journal.pone.0109443)
Supplement: Text S1 — Supporting information for the methods applied and results obtained. The details of methods are described under Methods section. The details of results are described under Results section. (DOCX) [file pone.0109443.s013.docx]

**Promoter analysis reveals globally differential regulation of human long non-coding RNA and protein-coding genes**

Tanvir Alam^a,1^, Yulia A. Medvedeva^a,1,e^, Hui Jia^b^, James B. Brown^c^, Leonard Lipovich^b,d,2,*^, Vladimir B. Bajic^a,2,*^

^a^King Abdullah University of Science and Technology (KAUST), Computational Bioscience Research Center (CBRC), Computer, Electrical and Mathematical Sciences and Engineering Division (CEMSE), Thuwal 23955-6900, Saudi Arabia; ^b^Center for Molecular Medicine and Genetics, Wayne State University, Detroit, Michigan 48201, USA; ^c^Department of Genome Dynamics, Life Sciences Division, Lawrence Berkeley National Laboratory, Berkeley, California 94720, USA; ^d^Department of Neurology, School of Medicine, Wayne State University, Detroit, Michigan 48201, USA; ^e^Present address: Institute of Personal and Predictive Medicine of Cancer, Badalona 08916, Spain, and Vavilov Institute of General Genetics, Moscow, 119991, Russia; ^1,2^Contributed equally;^*^To whom correspondence may be addressed. E-mail: [llipovich@med.wayne.edu](mailto:llipovich@med.wayne.edu) or [vladimir.bajic@kaust.edu.sa](mailto:Vladimir.bajic@kaust.edu.sa)

## Methods

###### Gene-centric lncRNA catalog construction

###### Consistent with our promoter selection procedure for protein-coding genes, we selected one transcript per gene for the lncRNA genes. The selection of a single reference transcript per gene was done for two reasons: first, we are interested in counting genes, rather than in counting individual transcripts; second, and more important, it is imperative for our subsequent sequence feature analyses to avoid promoter redundancy and to ensure that we sample each promoter only once. Accordingly, we performed genomic positional two-dimensional overlap of every transcript in our original combined BED file (of the multiple public sources listed above) with every other transcript in the same file. We considered only genes located on chromosomes 1-22, X, and Y. We filtered out all transcripts that could be same-strand hosts of any snRNA, snoRNA, or microRNA. All lncRNAs that had same-strand overlaps, anywhere in their promoters, exons, and/or introns, with any known snoRNAs or microRNAs were eliminated before we arrived at the 18,498 genes (Dataset S1b).

###### Promoter sets processing

We selected promoter sequences for each TSS of both coding and noncoding genes. If two or more sequences shared the same TSS on the same strand, only one promoter region was included in the promoter set. In cases when the same promoter appears in both coding and non-coding sets, we excluded it from both sets to reduce the ambiguity. The resulting set contained promoters of 18,787 protein-coding and 18,487 lncRNA genes (Dataset S1a-b).

Additionally, to avoid biases caused by repetitive elements we performed analysis in two ways: using a complete promoter set (CPS) as described above and using a repeat-filtered promoter set (REFPS). We generated a “non-repetitive” promoter dataset excluding all promoters overlapping with simple repeats, low complexity regions or satellite repeats for at least 1 bp. Coordinates of repetitive elements were downloaded from the UCSC Genome Browser Database

(<http://hgdownload.cse.ucsc.edu/goldenPath/hg19/database/rmsk.txt.gz>, last modification date: 27 April, 2009). The resulting set contains 7,930 protein-coding and 10,607 lncRNA (Dataset S1c-d) genes (repeat-filtered promoter set, REFPS). In the generation of CPS and REFPS, overlapping but not identical promoter regions were included independently, despite the possibility of false positive motif enrichment caused by oversampling these redundant genomic regions (approximately 1%, e.g. 600 and 200 promoters from CPS and REFPS, respectively). However, since these cases are rather rare they will not impact significantly the results.

We selected a shorter region ([-250 …+250]) around TSS (DataSet S1e-h) to check the support for our lncRNA-promoter-enriched TSS by DNAseI and ENCODE ChIP-seq for both CPS and REFPS.

###### K-mers

For each promoter we calculated frequencies of mono-, di-, tri- consecutive nucleotides in the upstream [-1000,-1] and downstream [+1,+1000] regions relative to TSSs. This generated 168 = (2*(4+16+64)) features. We also checked the observed/expected ratio[[1](#_ENREF_1)] of nucleotide combinations:

$$\begin{matrix} \frac{Obs}{Exp}b_{i}b_{j}= \frac{\#(b_{i}b_{j})}{\#b_{i}*\#b_{j}} *N, \\ \frac{Obs}{Exp}b_{i}b_{j}b_{k}= \frac{\#(b_{i}b_{j}b_{k})}{\#b_{i}*\#b_{j} *\#b_{k}} *N^{2}, \end{matrix}$$

$\frac{\mathrm{Obs}}{\mathrm{Exp}}b_{i}b_{j}= \frac{\#(b_{i}b_{j})}{\#b_{i}{\cdot\#b}_{j}} \cdot N$where $b_{i}{, b}_{j}{,b}_{k}$ are nucleotides A,C,G or T; $\#b_{i}{, \#b}_{j}{,\#b}_{k}$,$\#(b_{i}b_{j}), \#(b_{i}b_{j}b_{k})$ are numbers of mono-, di- and trinucleotides found in a promoter set; $N$is the total number of nucleotides in a promoter set.

###### AT and CG skew (SKEW)

Promoter regions are known to have very prominent AT- and CG-skews [[2](#_ENREF_2)] around TSS. For each promoter we calculated AT- and CG-skews in a sliding overlapping window of 100 bp with a shift of 50 bp at each step. This resulted into (39*2 = 78 features).

We calculated AT- and CG-skews using the following formulas:

$$\begin{matrix} AT_{skew}= \frac{\#A-\#T}{\#A+\#T}, \\ CG_{skew}= \frac{\#C-\#G}{\#C+\#G}, \end{matrix}$$

where *#A*, *#T*, *#C* and *#G* represent the number of A, T, C and G in a window, respectively.

###### Palindromes (PALIN)

Promoters often form intrastrand hairpin structures [[3](#_ENREF_3)]. To incorporate this information we sought for all complementary palindromes of length six or more using Bioinformatics toolbox (<http://www.mathworks.com/help/toolbox/bioinfo/ref/palindromes.html>) for MATLAB 2011a. For each promoter we counted the number of nucleotides overlapping with any palindrome structure in a sliding window of 100 bp with a shift of 50 bp at each step, which finally resulted in 39 features.

###### Word commonality (WC)

It was previously shown that protein-coding gene promoters contain patterns which are rare in the rest of the genome [[4](#_ENREF_4)]. To estimate the commonality of hexamer oligonucleotides in promoters of both protein-coding and lncRNA genes we took randomly 1000 sequences of 1,000 bp from a human genome (hg19) and counted the frequency of all possible 4^6^ = 4,096 hexamers. The frequency of each hexamer was normalized by min-max normalization to range [0,1], so the least common hexamer had the score 0 and the most frequent one had the score 1. For each promoter we calculated a score in each sliding window of 100 bp with 50 bp shift at each step by adding the score of each hexamer occurring in a window at any of the strands. This produced 39 features.

###### Transcription factor binding sites (TFBSs)

We selected significantly overrepresented TFBSs in promoters of protein-coding vs. promoters of lncRNA (and vice versa) gene sets (p-value <= 0.05, right sided Fisher’s exact test with Benjamini-Hochberg multiple testing for controlling false discovery rate (FDR)[[5](#_ENREF_5)]). We selected only TFBS having distance less than 0.7 from the “ideal” point and ranked them based on the distance (see below).

###### Distance from the ideal TFBS

For each TFBS model we calculated the Euclidean distance from the “ideal” motif family (MF), whose motifs are present in all promoters of one set and absent in another, using the concept of the distance of a predictor from an ideal predictor [[6](#_ENREF_6)]. In our case the formulae are as follows:

$$\begin{matrix} Dist_{c}\left(\overline{TFBS}, \overline{ideal_{c}}\right)= \sqrt{\left(TFBS_{c}-1\right)^{2}+ \left(TFBS_{nc}-0\right)^{2}}, \\ Dist_{nc}\left(\overline{TFBS}, \overline{ideal_{nc}}\right)= \sqrt{\left(TFBS_{c}-0\right)^{2}+ \left(TFBS_{nc}-1\right)^{2}}, \end{matrix}$$

where $\bar{\mathrm{TFBS}}=\left( \mathrm{TFBS}_{c}, \mathrm{TFBS}_{\mathrm{nc}} \right)$ $\mathrm{TFBS}_{c}$ and $\mathrm{TFBS}_{\mathrm{nc}}$ represent proportion of promoters of protein-coding and lncRNA genes, respectively, that contain the TFBS; $\bar{\mathrm{ideal}_{c}}=\left( 1, 0 \right)$ corresponds to the TFBS present in all promoters of protein-coding genes and absent in promoters of lncRNA genes; $\bar{\mathrm{ideal}_{\mathrm{nc}}}=\left( 0, 1 \right)$ corresponds to the TFBS present all promoters of lncRNA genes and absent in promoters of protein-coding genes. According to this definition, a TFBS, which is present in all lncRNA promoters and absent in all protein-coding gene promoters, will have a zero distance from $\overline{ideal_{nc}}$ and a distance of √2 from $\overline{ideal_{c}}$. All the TFBS, overrepresented in lncRNA promoters are ranked based on the distance from $\overline{ideal_{nc}}$. We selected only TFBSs having distance less than 0.7 (approximately ½ of a maximum possible distance) from the “ideal” point $\overline{ideal_{nc}}$ and ranked them based on the distance. The lower the distance from the “ideal” point $\overline{ideal_{nc}}$ the higher is the ranking of a TFBS. Similarly, a TFBS, which is present in all protein-coding gene promoters and absent in all lncRNA gene promoters, will have a zero distance from$\overline{ideal_{c}}$ and a distance of √2 from $\overline{ideal_{nc}}$. All the TFBS, overrepresented in promoters of protein-coding gene promoters are ranked based on the distance from $\overline{ideal_{c}}$. We selected only TFBSs having distance less than 0.7 (approximately ½ of a maximum possible distance) from the “ideal” point $\overline{ideal_{c}}$ and ranked them based on the distance. The lower the distance from the “ideal” point $\overline{ideal_{c}}$ the higher is the ranking of a TFBS. To check overrepresentation of TFBSs, we applied the right-sided exact Fisher’s test to contingency tables with Benjamini-Hochberg multiple testing for controlling false discovery rate (FDR).

###### *Ab initio* motif search

To compensate for missing variations of TFBSs for a particular TF and non-complete set of TFBS models we sought for *ab initio* identified motif families (MFs) using Dragon Motif Finder (DMF) [[7](#_ENREF_7)]. The list of parameters that we used for *ab initio* motif finding is as follows:

EMThreshold Range:0.90,0.70,-.05

motifNum:25

Motiflength:6 - 15

MotifExtension:4

Masking:1

We selected only MFs overrepresented in the protein-coding gene promoters as compared to lncRNA gene promoters and vice versa (p-value ≤ 0.05, right-sided Fisher’s exact test with Bonferroni correction) and ranked them based on the distance from ideal MF as mentioned above for ranking of TFBS.

To remove the redundant MFs, we measured the similarity between any two MFs using Pearson's correlation co-efficient. For two PWMs, PWM*_A_ and* PWM*_B_ -* having length *L_A_* and *L_B_* respectively, we move A over B in forward and reverse complemented strands in window based fashion and calculated the similarity between shorter PWM and corresponding part of the longer PWM for each position. For a single alignment of window from two PWMs, we took the average similarities for corresponding positions of two windows and call it as window-based-similarity. We chose the maximum of all window-based-similarities as a measure of similarity between two PWMs and normalized in the range from 0 to 1. Then we took the top ranked one MF and discard other similar MFs having similarity more than 0.70 with it. From the remaining MFs we took the highest ranked MF and discard remaining MFs in the same fashion.

**Comparison of *ab initio* identified PWMs to PWMs of known TFBSs**

###### We compared *ab initio* identified MFs to the HOCOMOCO database v8. For the comparison we used Tomtom [[8](#_ENREF_8)] software. We chose Pearson’s correlation coefficient as a measure of pairwise PWMs similarity. For each known TFBS, we selected *ab initio* determined MF having similarity expressed in terms of q-value < 0.05 with complete scoring option provided in Tomtom.

###### Repetitive elements (RE)

For each promoter we calculated the number of nucleotides overlapping with any type of RE in any strand in a sliding window of 100 bp with 50 bp shift at each step. This generated 39 features, corresponding to 39 sliding windows. For CPS, we considered all types of REs. For REFPS, we used all types of repeat elements except simple repeat, low complexity or satellite repeats.

###### CpG islands (CGIs)

CGIs were generated using a program developed by G. Miklem and L. Hillier based on criteria [[1](#_ENREF_1)]. It is worth noting that we applied the algorithm to the non-repeat masked genome, to allow for the possibility that REs can function as promoters of lncRNA. This is a reasonable strategy in view of the recent discovery that primate-specific and rodent-specific REs have drastically modified the protein-coding gene promoters during mammalian evolution, dispersing intra-RE TFBSs to new promoters in the stem cell regulatory network [[9](#_ENREF_9)]. For each promoter we calculated the number of nucleotides overlapping with CGI in a sliding window of 100 bp with a shift of 50 bp at each step. This generated 39 features, corresponding to 39 windows.

###### Chromatin states (CS) and histone modification (HM) marks

We used coordinates of 15 different chromatin states [[10](#_ENREF_10)] for seven cell types with normal karyotype (GM12878, H1-hESC, HMEC, HSMM, HUVEC, NHEK, and NHLF) from the UCSC Genome Browser (<http://genome.ucsc.edu/cgi-bin/hgTrackUi?hgsid=244325981&c=chr21&g=wgEncodeBroadHmm>, downloaded on 27 December, 2012). For each promoter we calculated the percentage of the total number of positions overlapping with each of these 15 states in each of the 7 samples, which finally resulted into 105 = (7*15) features.

We also used ChIP-seq peaks for 12 separate HMs and other epigenetically related marks (EZH2, H2A.Z, H3K4me1, H3K4me2, H3K4me3, H3K9ac, H3K9me3, H3K27ac, H3K27me3, H3K36me3, H3K79me2 and CTCF) from the UCSC Genome Browser Database (<http://encodeproject.org/cgi-bin/hgTrackUi?hgsid=305974773&c=chr21&g=wgEncodeBroadHistone>, downloaded on 27 December, 2012) for the GM12878 cell line. For each promoter we calculated the percentage of the total number of positions overlapping with each of these 12 marks.

###### Model building

We used an ensemble of decision trees [[11](#_ENREF_11)] to generate a classification model and estimate its accuracy with 20-fold cross-validation. Random partition for 20-fold cross-validation on the whole dataset was used to assess the performance of the classifier model constructed in this study. Data set was divided randomly into 20 non-overlapping partitions. Then the model was trained on nineteen of these partitions that were subjected to normalization and performance was measured on the remaining testing partition. The whole process was repeated 20 times with different training and testing sets. Sensitivity, specificity and accuracy are estimated as the average across all testing folds.

We normalized the features in the training data to the mean value of zero and the standard deviation of one. The testing data is transformed based on the normalization parameters obtained on the training data. Using MATLAB version 2011a we built an ensemble model with 100 decision trees [[11](#_ENREF_11)] to make a classifier that distinguishes the promoters of protein-coding genes from promoters of lncRNA genes. We considered promoters of lncRNA genes as the positive set and promoters of protein-coding genes as the negative set. A predicted class for a promoter in a test set was determined by majority voting of 100 decision trees. We used the ‘Bag’ method of ‘fitensemble’ function of MATLAB 2011a with default parameters setting. We built models for two different promoter sets, CPS and REFPS, independently.

To avoid a potential bias cause by coding exons which might have been included into a region of [-1000,+1000] bp around the TSS, we also performed the analysis using only upstream promoter regions ([-1000,0] bp upstream of the TSS) from CPS (Dataset S2).

We also build a model for the upstream promoter regions ([-1000,0] bp) having no overlap with CGI to avoid a bias caused by more abundant presence of the CGIs in protein-coding vs. lncRNA promoters from CPS. This results in a set of 14795 lncRNA promoters and 6239 protein-coding gene promoters from CPS (Dataset S2). The model was built using all features except CGI related features. The model was run 5 times, by under sampling the lncRNA promoter set randomly each time, and with 20-fold cross validation.

###### Performance measures

The total number of true positive (TP), false negative (FN), false positive (FP), true negative (TN) predictions were used to determine sensitivity, specificity and accuracy as follows:

$$Sensitivity=\frac{TP}{\left(TP+FN\right)}\times100\%$$

$$Specificity=\frac{TN}{\left(FP+TN\right)}\times100\%$$

$$Accuracy=\frac{TP + TN}{\left(\text{ }TP+FN+FP+TN\right)}\times100\%$$

**Support for lncRNA-TSS-enriched TFBSs by DNAseI and ChIP-seq peaks from ENCODE**

For each lncRNA-enriched TF, we generated a genomewide set of lncRNA promoters that contained the corresponding HOCOMOCO TFBSs, and we then overlapped the genomic locations of each TFBS with the combined list of ENCODE DNAseI site genomic locations, testing for significant association of the lncRNA-promoter-enriched TFBSs with the DNAseI sites. We selected a shorter region ([-250 …+250]) around TSS (DataSet S1e-h) to check the support for our lncRNA-TSS-enriched TSS by DNAseI and ENCODE ChIP-seq. As ENCODE DNAseI peaks are very sharp, we constrained the promoters from 1000 bp to 250 bp and re-did the analysis, finding strong and significant overlaps of ENCODE DNAseI peaks from the 127 cell lines and ChIP-seq peaks with our lncRNA-promoter-enriched TFBSs. Tracks are provided for ENCODE ChIP-seq supported three TFBS which are overrepresented in promoters of lncRNA (DataSet S1). For the promoters with upstream ([-1000,0] bp upstream of the TSS) , we used the upstream region for checking the support by DNAseI and ChIP-seq.

**Expression analysis using RNA-seq data**

We consider genes with non-zero expression values in three ENCODE tier-1 cell lines (Gm12878, H1- hESC, K562) and one ENCODE tier-2 cell line (HUVEC) for the analysis. We got 10117, 11126, 9149 and 8789 lncRNAs with non-zero expression value from Gm12878, H1- hESC, K562, HUVEC cell lines respectively. We got 16254, 17041, 15530 and 15901 non-zero protein-coding genes with non-zero expression value from Gm12878, H1- hESC, K562, HUVEC cell lines respectively. To control the distribution of expression at similar level, for each lncRNA having non-zero expression value we consider all protein-coding genes with maximum 1% deviation of expression. To make one-to-one correspondence between lncRNA gene and protein-coding gene, we select the protein-coding gene with nearest expression value. If two or more lncRNA genes have same protein-coding gene as the nearest neighbor then we keep one of the lncRNA genes and discard rest of them. In this way, we select 8361, 8047, 7801 and 7328 lncRNA gene and protein-coding genes from Gm12878, H1-hESC, K562 and HUVEC cell lines respectively.

## Results

###### Nucleotide patterns in promoters of protein-coding and lncRNA genes

Although most protein-coding gene promoters are GC-enriched [[12](#_ENREF_12)], we find that lncRNA gene promoters are not, perhaps due to different functions or RNA secondary structures relative to those of mRNA 5’UTRs [[13](#_ENREF_13)]. This is also consistent with the association of CpG-island promoters and highly-expressed protein-coding genes and with the known tissue-specificity of multiple lncRNAs: tissue-specific lncRNAs might need to be expressed in only one tissue, and to be constitutively repressed in all others [[14](#_ENREF_14)].

In the dinucleotide data, the CpG dinucleotide demonstrated the most pronounced and intriguing difference, having 1.4-fold and 1.7-fold enrichment in upstream and downstream regions of promoters of protein-coding genes vs. promoters of lncRNA genes, respectively (Table S1). The similar tendency holds for REFPS. This is consistent with the observation that CpG islands serve as promoters for highly expressed protein-coding genes.

Most of the other dinucleotides do not show any significant difference for protein-coding and lncRNA promoters. Most of the trinucleotide differences for both upstream and downstream regions were derivatives of the CpG overrepresentation in promoters of protein-coding genes. However, for downstream region there are two trinucleotides overrepresented: AAA in promoters of protein-coding genes and CAC in promoters of lncRNA genes. For REFPS only, CpG-derived trinucleotides remain overrepresented in promoters of protein-coding genes.

###### Epigenetic features associated with promoters of protein-coding and lncRNA genes

The association of the two promoter groups with group-specific CSs is an excellent indicator that CSs could play a critical role in controlling differential regulation of protein-coding and lncRNA genes. Yet, our results on chromatin properties of promoters should be considered with care. The cDNAs and ESTs which gave rise to our lncRNA collection and RefSeq protein-coding genes represent the entire spectrum of human tissues and cell types, while the chromatin data is confined to nine cell lines, not necessarily reflecting the promoter states in all other tissues that the RNAs came from. Promoters of lncRNAs expressed in several cell types and tissues show clear depletion of H3K4me3, H3K9me3, H3K27me3 and H3K36me3 marks around TSSs of lncRNA genes as compared to TSSs of protein-coding genes [[15](#_ENREF_15)], which is in concordance with our results, although we do not consider the expression of lncRNAs in the same cell line for which the ChIP-Seq histone modifications results are studied. In contrast, our recent ENCODE effort [[14](#_ENREF_14)] suggested that for K562 cell line, at least H3K4me1, H3K27me3, H3K36me3 and H4K20me1 show higher level in lncRNA gene promoters, compared to protein-coding gene promoters. This controversy may occur due to several factors: different regulatory patterns in normal cells from which lncRNA cDNA/ESTs originated, relative to ENCODE’s cancer cell lines; variations between lncRNA sets used by different authors, which are less standardized than sets of protein-coding genes; and incomplete coverage of cell-type-specific lncRNAs.

## Enhancer-associated CSs at protein-coding and lncRNA promoters

It is noteworthy, that relationship of enhancer CSs to lncRNA and coding-gene promoters is ambiguous. Among four categories of enhancers one state was overrepresented in lncRNA gene promoters and two others in promoters of coding genes, although the overall coverage of both sets with enhancers is low (Figure. S3, Table S3). The enhancer CS overrepresented in lncRNA gene promoters (weak enhancers) are characterized by the presence of H3K4me1 (a known mark of enhancers), but concomitant depletion of H3K4me2 (promoters, enhancers, and CpG islands) / H3K4me3 (active promoters) marks and a decreased level of H3K27ac/H3K9ac. In contrast, enhancer CSs overrepresented in protein-coding gene promoters are enriched with H3K4me1 as well as H3K4me2, and to a lesser extent with H3K4me3, H3K27ac and H3K9ac. None of these marks were enriched at lncRNA promoters, which suggest a new type of enhancer chromatin organization there.

**Differences in repeat-filtered (REFPS) and complete set (CPS) of promoters**

To avoid bias caused by repetitive elements we performed analysis in two ways: on a complete promoter set (CPS) and repeat-filtered promoter set (REFPS). CPS results account for the biology of repeat-mediated dispersal of sequence features, whereas REFPS results only include single-copy sequences. Although the results for both sets were quite similar, minor differences appeared.

## References

1. Gardiner-Garden M, Frommer M (1987) CpG islands in vertebrate genomes. J Mol Biol 196: 261.

2. Touchon M, Nicolay S, Arneodo A, d’Aubenton-Carafa Y, Thermes C (2003) Transcription-coupled TA and GC strand asymmetries in the human genome. FEBS Lett 555: 579-582.

3. Lu L, Jia H, Droge P, Li J (2007) The human genome-wide distribution of DNA palindromes. Funct Integr Genomics 7: 221-227.

4. Roberts L, Steele N, Reeves C, King G (1995) Training neural networks to identify coding regions in genomic DNA. Fourth International Conference on Artificial Neural Networks 399-403.

5. Benjamini Y, Hochberg Y (1995) Controlling the false discovery rate: a practical and powerful approach to multiple testing. Journal of the Royal Statistical Society Series B (Methodological): 289-300.

6. Bajic VB (2000) Comparing the success of different prediction software in sequence analysis: a review. Brief Bioinform 1: 214-228.

7. Marchand B, Bajic V, Kaushik D (2011) Highly scalable ab initio genomic motif identification. Proceedings of 2011 International Conference for High Performance Computing, Networking, Storage and Analysis: 1-10.

8. Gupta S, Stamatoyannopoulos JA, Bailey TL, Noble WS (2007) Quantifying similarity between motifs. Genome Biol 8: R24.

9. Kunarso G, Chia NY, Jeyakani J, Hwang C, Lu X, et al. (2010) Transposable elements have rewired the core regulatory network of human embryonic stem cells. Nat Genet 42: 631-634.

10. Ernst J, Kheradpour P, Mikkelsen TS, Shoresh N, Ward LD, et al. (2011) Mapping and analysis of chromatin state dynamics in nine human cell types. Nature 473: 43-49.

11. Breiman L, Friedman JH, Olshen RA, Stone CJ (1984) Classification and Regression Trees: Chapman and Hall.

12. Bajic VB, Tan SL, Christoffels A, Schönbach C, Lipovich L, et al. (2006) Mice and men: their promoter properties. PLoS Genet 2: e54.

13. Davuluri RV, Grosse I, Zhang MQ (2001) Computational identification of promoters and first exons in the human genome. Nat Genet 29: 412-417.

14. Derrien T, Johnson R, Bussotti G, Tanzer A, Djebali S, et al. (2012) The GENCODE v7 catalog of human long noncoding RNAs: Analysis of their gene structure, evolution, and expression. Genome Res 22: 1775-1789.

15. Sati S, Ghosh S, Jain V, Scaria V, Sengupta S (2012) Genome-wide analysis reveals distinct patterns of epigenetic features in long non-coding RNA loci. Nucleic Acids Res 40: 10018-10031.
